# Bioinspired enantioselective synthesis of crinine-type alkaloids *via* iridium-catalyzed asymmetric hydrogenation of enones†
†Electronic supplementary information (ESI) available. See DOI: 10.1039/c7sc02112g



**DOI:** 10.1039/c7sc02112g

**Published:** 2017-07-03

**Authors:** Xiao-Dong Zuo, Shu-Min Guo, Rui Yang, Jian-Hua Xie, Qi-Lin Zhou

**Affiliations:** a State Key Laboratory and Institute of Elemento-Organic Chemistry , College of Chemistry , Nankai University , Tianjin 300071 , China . Email: jhxie@nankai.edu.cn ; Email: qlzhou@nankai.edu.cn; b Collaborative Innovation Center of Chemical Science and Engineering (Tianjin) , Nankai University , Tianjin 300071 , China

## Abstract

A bioinspired enantioselective synthesis of crinine-type alkaloids has been developed by iridium-catalyzed asymmetric hydrogenation of racemic cycloenones. The method features a biomimetic stereodivergent resolution of the substrates bearing a remote arylated quaternary stereocenter. Using this protocol, 24 crinine-type alkaloids and 8 analogues were synthesized in a concise and rapid way with high yield and high enantioselectivity.

## Abstract 2

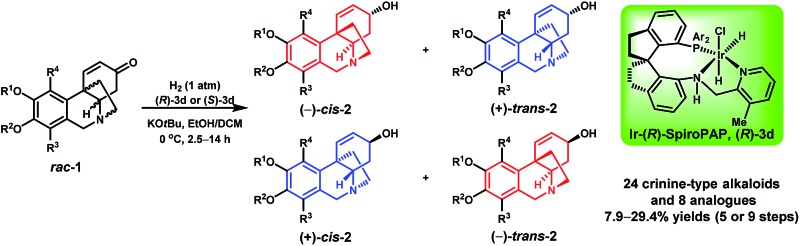
A bioinspired enantioselective synthesis of crinine-type alkaloids was developed by iridium-catalyzed asymmetric hydrogenation of enones, providing 24 crinine-type alkaloids and 8 analogues with high yield and high enantioselectivity.

## Introduction

Amaryllidaceae alkaloids have long attracted the attention of synthetic chemists due to their significant bioactivities such as antitumor, antiviral, and anti-acetylcholinesterase activities, and the fascinating diversity of their structures. To date, more than 500 Amaryllidaceae alkaloids have been isolated from Amaryllidaceae plants.^1^ However, only one member of them, galanthamine, has been approved as a prescription drug for the treatment of Alzheimer’s disease.^2^ Because these alkaloids are structurally intricate, general methods for their synthesis are lacking, which has hindered the study of their medicinal chemistry.

**Fig. 1:**
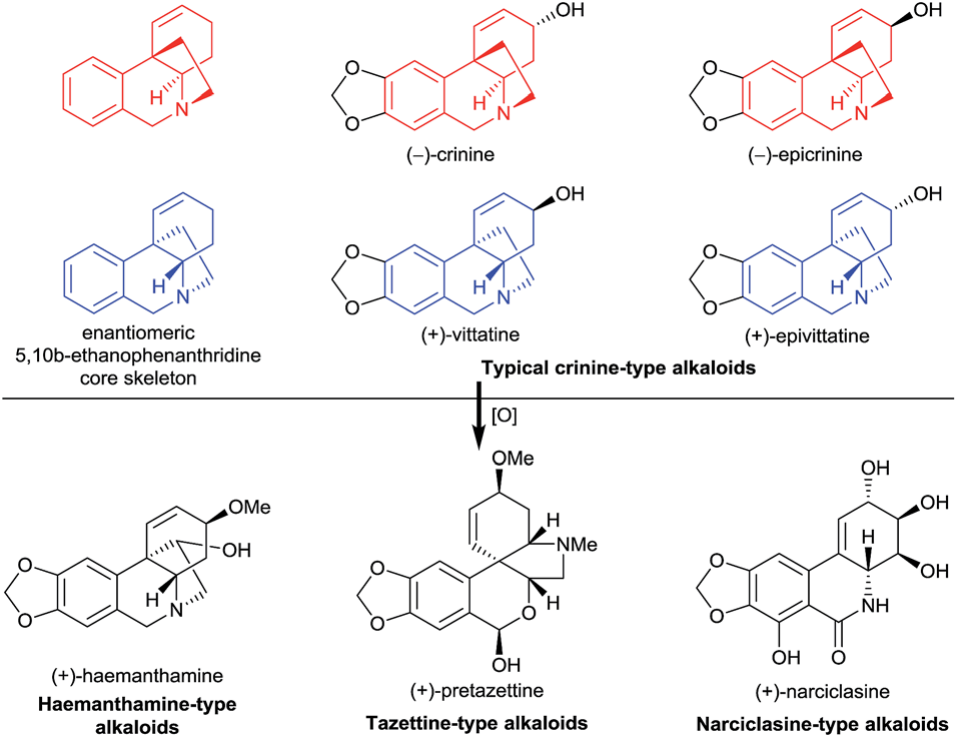
Representative crinine-type alkaloids and Amaryllidaceae alkaloids derived possibly from crinine-type alkaloids.

We recently became particularly interested in crinine-type alkaloids, a large subclass (more than 80 have been isolated) of the Amaryllidaceae alkaloid family, that possess an antipodal chiral 5,10*b*-ethanophenanthridine core skeleton with a benzylic all-carbon quaternary centre^1*c*^ (Fig. 1). For example, the parent alkaloid of the subclass, (–)-crinine, was first isolated in 1955 from the bulbs of two unidentified *Crinum* species from South Africa;^3^ (+)-vittatine, which has the opposite configuration to that of (–)-crinine, is another crinine-type alkaloid.^4^ Importantly, the crinine-type alkaloids are considered to be biogenic precursors of several types of Amaryllidaceae alkaloids including tazettine-, haemanthamine-, and narciclasine-type alkaloids^5^ (Fig. 1). Thus, exploring efficient methods for the rapid synthesis of diverse crinine-type alkaloids is particularly important and desirable. However, although great efforts have been devoted to the development of synthetic methods to obtain crinine-type alkaloids, most of the reported approaches provided racemic products.^6^

**Scheme 1:**
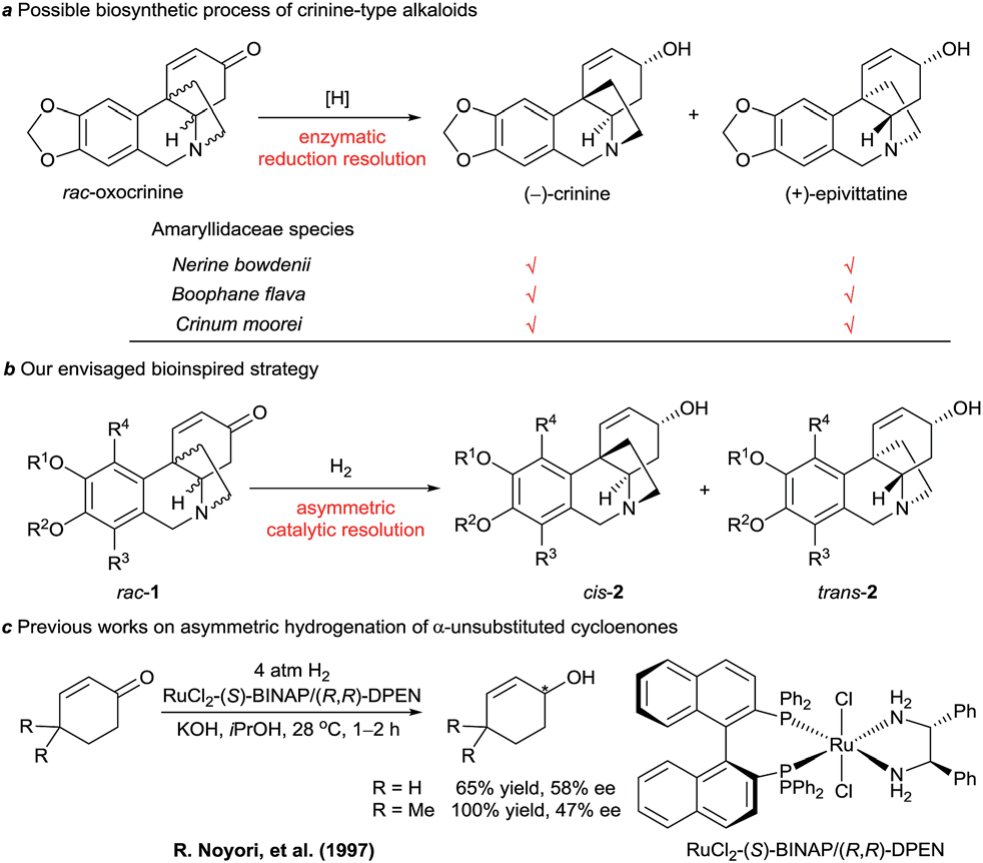
Possible biosynthetic process of crinine-type alkaloids and our envisaged bioinspired strategy.

We noticed that some Amaryllidaceae species can produce two similar alkaloids with enantiomeric skeletons. For example, both (–)-crinine and (+)-epivittatine have been isolated simultaneously from the bulbs of *Nerine bowdenii*, *Boophane flava*, and *Crinum moorei* (Scheme 1a).^4b,9^ Inspired by this natural phenomenon and the biosynthetic pathway of crinine-type alkaloids, we envisaged a new synthetic strategy that mimics the enzymatic reduction resolution of racemic oxocrinines **1** using synthetic chiral catalysts (Scheme 1b). Because racemic oxocrinines **1** can be prepared in only four steps in high yields by a biomimetic intramolecular phenolic oxidative coupling of *O*-methylnorbelladine derived from l-phenylalanine and l-tyrosine,^6*f*^ this asymmetric catalytic stereodivergent resolution strategy^10^ will provide a concise and rapid approach to diverse crinine-type alkaloids and their analogues.

## Results and discussion

Although asymmetric hydrogenation of the ketone group of α,β-unsaturated ketones is an efficient way for synthesising optically active allylic alcohols,^11^ the asymmetric hydrogenation of cyclohexenones with no substituent at the α-position is still a challenge (Scheme 1c).^12^ To find efficient chiral catalysts for the asymmetric hydrogenation of racemic oxocrinines **1**, we investigated chiral spiro iridium catalysts Ir-SpiroPAP (**3**)^13^ developed in our laboratory. The racemic oxocrinines **1** were prepared in four steps with 55–66% yield from commercially available starting materials using Node’s biomimetic procedure^6*f*^ (see the ESI†). Asymmetric hydrogenation of *rac*-**1a** (R^1^, R^2^ = –CH_2_–, R^3^, R^4^ = H) was firstly carried out using the catalyst (*R*)-**3a**. When the hydrogenation of *rac*-**1a** was performed under 1 atm of H_2_ pressure with KO*t*Bu as the base in EtOH at room temperature, the reaction was completed within 0.8 h and the desired products (–)-*cis*-**2a** and (+)-*trans*-**2a** were obtained in 91% yield with a (–)-*cis*-**2a**/(+)-*trans*-**2a** ratio of 11 : 88. However, the enantiomeric excess (ee) of the major product (+)-*trans*-**2a** was only 27% (Table 1, entry 1). A comparison of the various Ir-SpiroPAP catalysts showed that (*R*)-**3d**, containing P(3,5-di-*tert*-butylphenyl)_2_ groups and a 3-Me–pyridine moiety, is the best catalyst, which afforded the products (–)-*cis*-**2a** and (+)-*trans*-**2a** in 93% yield with 97% ee and 87% ee, respectively, in a ratio of 45 : 55 (entry 4). The effect of solvent was examined, and *n*PrOH gave comparable results to EtOH (entry 8). In addition to KO*t*Bu, other bases such as KOH, K_2_CO_3_ and Et_3_N can also be used, although the reaction with Et_3_N needs a longer time for completion and the yield is lower (entry 11). Further study of the reaction temperature, base concentration, hydrogen pressure, and co-solvent established the optimal reaction conditions to be as follows: 0.1 mol% (*R*)-**3d**, [*rac*-**1a**] = 0.17 M, [KO*t*Bu] = 0.008 M, 0 °C, 1 atm of H_2_, EtOH/DCM (5 : 2). Under these conditions, hydrogenation of *rac*-**1a** afforded two crinine-type alkaloids: (–)-*cis*-**2a** (crinine, 97% ee) and (+)-*trans*-**2a** (epivittatine, 93% ee) in 94% overall yield with a (–)-*cis*-**2a**/(+)-*trans*-**2a** ratio of 46 : 54 (entry 16). It is to be noted that we have tried the direct isolation of the mixture of (–)-*cis*-**2a** and (+)-*trans*-**2a** by chromatography on a Sephadex LH-20 column according to Codina’s protocol,^14^ but it was demonstrated to be difficult. Fortunately, we found that the mixture can be isolated by converting them into benzoyl esters, and after hydrolysis of the isolated benzoyl esters (–)-*cis*-**2a** and (+)-*trans*-**2a** can be obtained in pure form with high yield.

**Table 1:** Optimization of the reaction conditions for the asymmetric hydrogenation of *rac*-**1a** ^*a*^

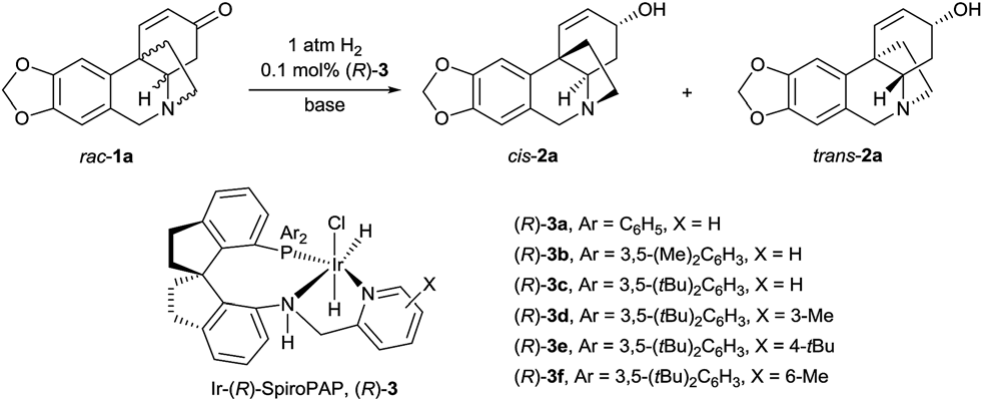								
Entry	(*R*)-**3**	Sol.	Base	Time (h)	Yield^*b*^ (%)	*cis*/*trans* ^*c*^	ee^*d*^ (%)	
							*cis*	*trans*
1	(*R*)-**3a**	EtOH	KO*t*Bu	0.8	91	11 : 88	90	27
2	(*R*)-**3b**	EtOH	KO*t*Bu	0.5	89	15 : 85	88	23
3	(*R*)-**3c**	EtOH	KO*t*Bu	0.8	95	46 : 54	90	86
4	(*R*)-**3d**	EtOH	KO*t*Bu	0.5	93	45 : 55	97	87
5	(*R*)-**3e**	EtOH	KO*t*Bu	1.0	90	42 : 58	91	77
6	(*R*)-**3f**	EtOH	KO*t*Bu	0.3	95	36 : 64	94	64
7	(*R*)-**3d**	MeOH	KO*t*Bu	0.7	92	40 : 60	92	76
8	(*R*)-**3d**	*n*PrOH	KO*t*Bu	0.8	91	43 : 57	95	90
9	(*R*)-**3d**	EtOH	KOH	0.3	90	46 : 54	95	87
10	(*R*)-**3a**	EtOH	K_2_CO_3_	0.5	91	46 : 54	95	87
11	(*R*)-**3d**	EtOH	Et_3_N	19	79	38 : 62	90	90
12^*e*^	(*R*)-**3d**	EtOH	KO*t*Bu	0.3	90	44 : 56	99	85
13^*f*^	(*R*)-**3d**	EtOH	KO*t*Bu	3	93	46 : 54	98	88
14^*g*^	(*R*)-**3d**	EtOH	KO*t*Bu	7.5	91	46 : 54	94	88
15^*h*^	(*R*)-**3d**	EtOH	KO*t*Bu	9	89	46 : 54	99	90
16^*i*^	(*R*)-**3d**	EtOH	KO*t*Bu	8.5	94	46 : 54	97	93

^*a*^Reaction conditions: 1 mmol scale, [**1a**] = 0.17 M, 0.1 mol% of (*R*)-**3**, [base] = 0.017 M, solvent (6.0 mL), 1 atm of H_2_, room temperature (22–27 °C), 100% conversion.

^*b*^Isolated yield of the mixture of (–)-*cis*-**2a** and (+)-*trans*-**2a**.

^*c*^The ratio of *cis* to *trans* was determined by ^1^H NMR.

^*d*^The ee values of (–)-*cis*-**2a** and (+)-*trans*-**2a** were determined by chiral HPLC (Chiralcel OD-3 column) after converting them into benzoyl esters.

^*e*^Under 5 atm of H_2_.

^*f*^At 0 °C.

^*g*^At 0 °C, and [KO*t*Bu] = 0.034 M.

^*h*^At 0 °C, and [KO*t*Bu] = 0.008 M.

^*i*^At 0 °C, [KO*t*Bu] = 0.008 M, and DCM as co-solvent (ethanol/DCM = 5 : 2).

Using this bioinspired asymmetric hydrogenation stereodivergent resolution method, a range of crinine-type alkaloids and analogues were synthesized from the corresponding racemic oxocrinines **1** (Table 2). In each reaction, two crinine-type alkaloids or analogues were obtained in high yield with high enantioselectivity. The absolute configurations of the products were determined by the configuration of the catalyst.

**Table 2:** Asymmetric syntheses of crinine-type alkaloids and analogues by hydrogenation of *rac*-**1** catalyzed by (*R*)-**3d** and (*S*)-**3d** ^*a*^

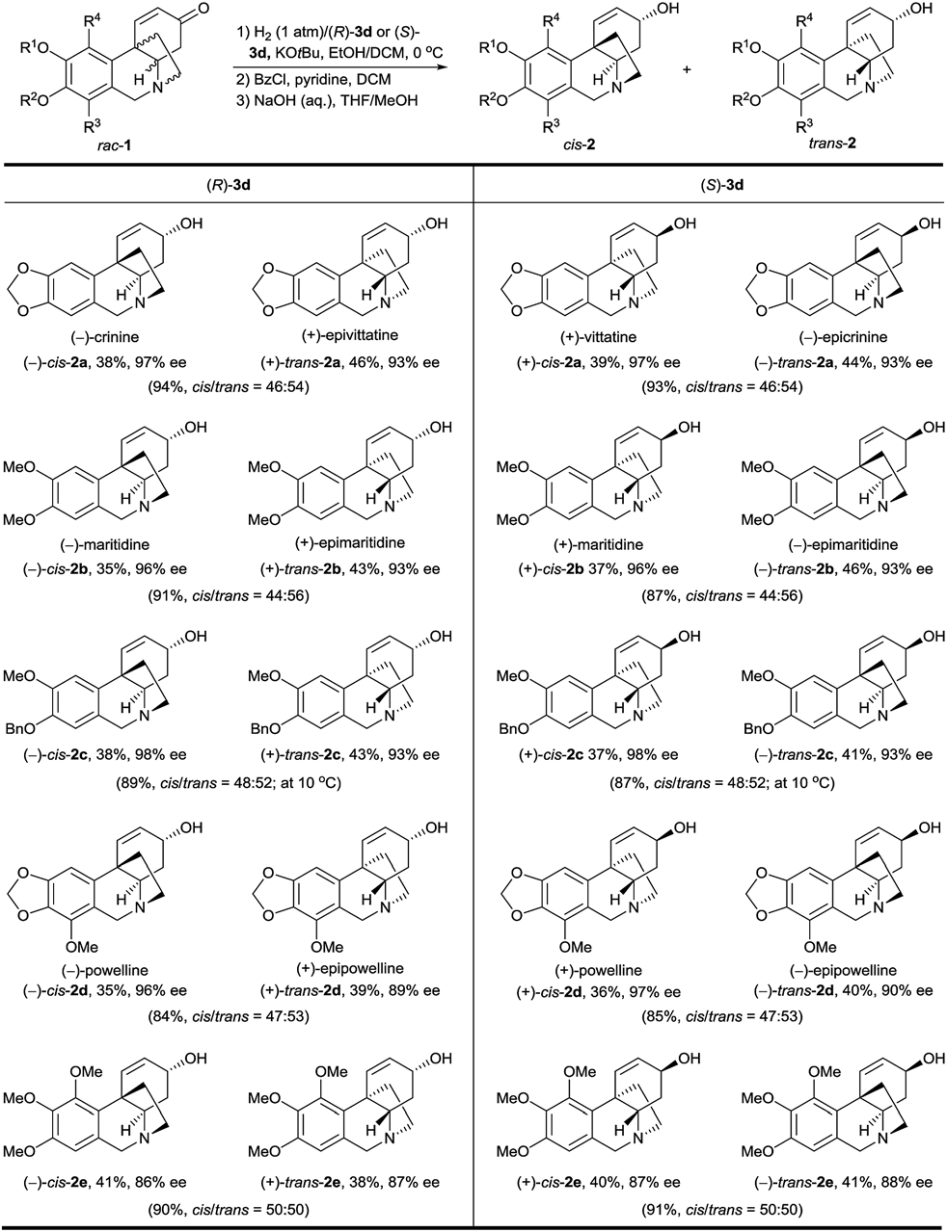

^*a*^Reaction conditions: 1 mmol scale, [**1**] = 0.17 M, 0.1 mol% of (*R*)-**3d** or (*S*)-**3d**, [KO*t*Bu] = 0.008 M, EtOH/DCM (5 : 2, 6.0 mL), 1 atm H_2_, 0 °C, 100% conversion. The *cis*/*trans* ratio was determined by ^1^H NMR. The products were obtained by converting them into benzoyl esters, followed by chromatography on silica gel and hydrolysis of the resulting isolated products with NaOH aqueous solution. The ee values of the products were determined by chiral HPLC analysis of the corresponding benzoyl esters. All the yields are isolated yields.

**Scheme 2:**
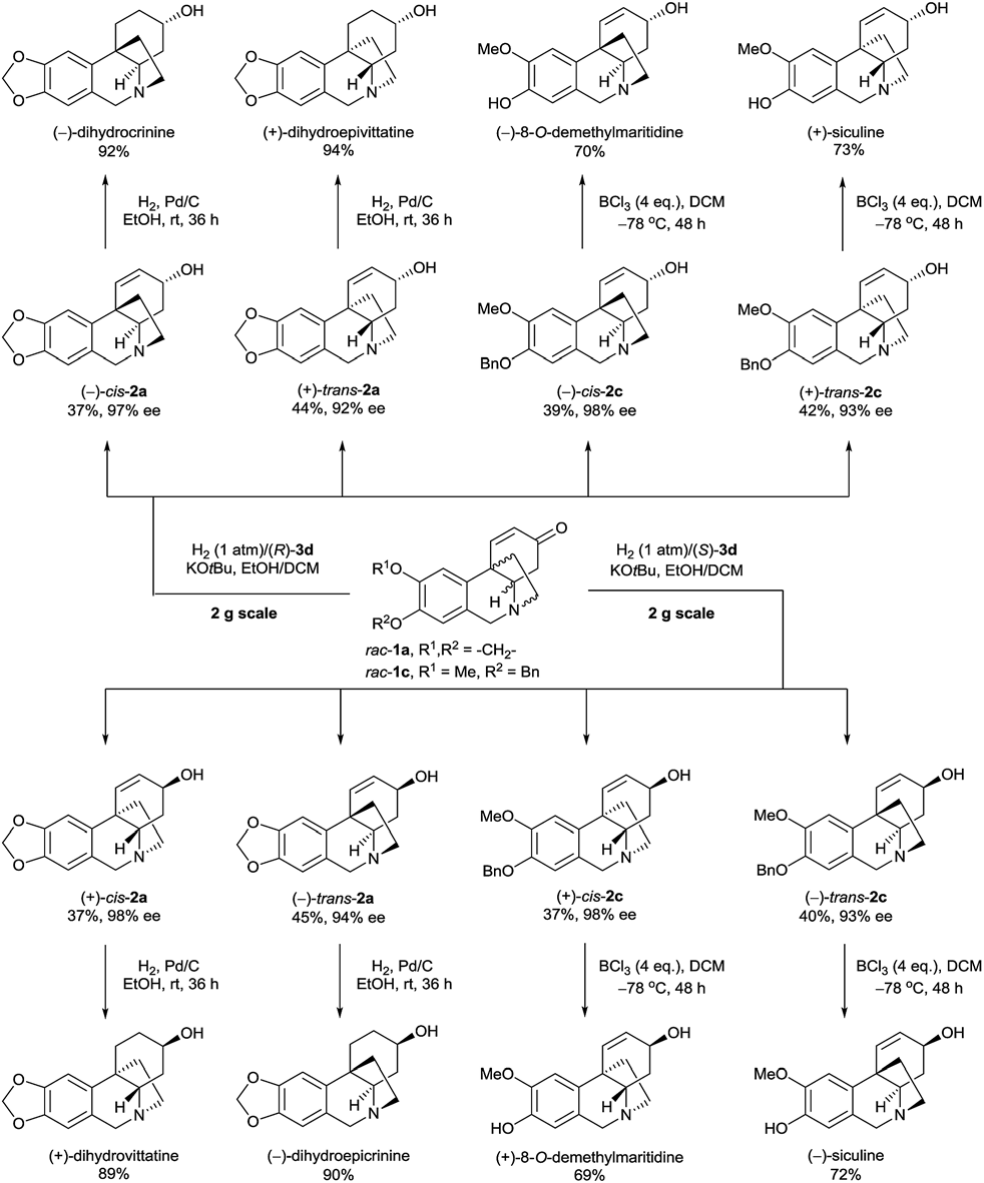
Asymmetric syntheses of 8-*O*-demethylmaritidines, siculines and dihydrocrinine-type alkaloids on the gram-scale.

The Ir-SpiroPAP-catalyzed asymmetric hydrogenation of oxocrinines **1** could be performed on a multigram scale (Scheme 2). For example, hydrogenation of substrates *rac*-**1a** and *rac*-**1c** on a 2 g scale produced (–)-*cis*-**2a** and (+)-*trans*-**2a**, and (–)-*cis*-**2c** and (+)-*trans*-**2c** in the presence of the catalyst (*R*)-**3d** and produced (+)-*cis*-**2a** and (–)-*trans*-**2a**, and (+)-*cis*-**2c** and (–)-*trans*-**2c** in the presence of the catalyst (*S*)-**3d**, with high yields and high enantioselectivities. After saturation of the C=C bonds of alkaloids **2a** over Pd/C, four dihydrocrinine-type alkaloids were obtained: (–)-dihydrocrinine,^15^ (+)-dihydrovittatine,^16^ (+)-dihydroepivittatine,^17^ and (–)-dihydroepicrinine.^17^ By debenzylation of alkaloids **2c** with boron trichloride (BCl_3_), four naturally occurring crinine-type alkaloids were obtained: (+)-8-*O*-demethylmaritidine,^18^ (–)-8-*O*-demethylmaritidine,^19^ (+)-siculine,^16^ and (–)-siculine.^20^

**Scheme 3:**
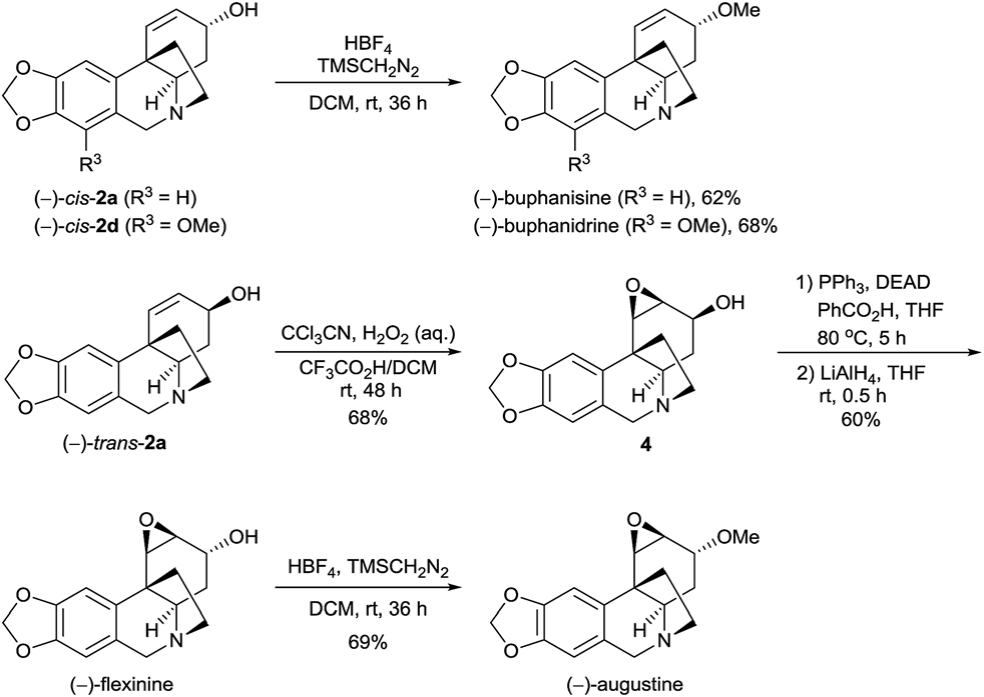
Asymmetric synthesis of *O*-methyl crinine-type alkaloids and analogues.

The methylation of the hydroxy group of the alkaloids **2** can offer *O*-methyl crinine-type alkaloids. When (–)-*cis*-**2a** and (–)-*cis*-**2d** were treated with TMSCH_2_N_2_ (trimethylsilyl diazomethane) in the presence of HBF_4_ in DCM, another two alkaloids, (–)-buphanisine and (–)-buphanidrine,^21^ were obtained in 62% and 68% yield, respectively (Scheme 3). According to Guillou’s procedure,^6j,22^ (–)-*trans*-**2a** was successfully oxidized with peroxymidic acid generated *in situ* from CCl_3_CN/H_2_O_2_ to the epoxide **4** in 68% yield in the presence of trifluoroacetic acid. A Mitsunobu reaction converted the epoxide **4** to the benzoate, followed by the removal of the benzoyl group with LiAlH_4_, yielding (–)-flexinine^23^ in 60% yield (2 steps). The (–)-flexinine was reacted with TMSCH_2_N_2_ in the presence of HBF_4_ in DCM to produce (–)-augustine in 69% yield. Thus, the enantioselective syntheses of (–)-buphanisine, (–)-buphanidrine, (–)-flexinine, and (–)-augustine were also achieved.

## Conclusions

In conclusion, we have developed a bioinspired strategy for the rapid enantioselective synthesis of crinine-type alkaloids. This strategy features an iridium-catalyzed asymmetric hydrogenation of racemic cycloenones with a remote arylated quaternary stereocenter *via* stereodivergent resolution. Using this new strategy, we synthesized a total of 24 crinine-type alkaloids and 8 analogues in 7.9–29.4% yields in only five or nine steps (seven or eleven steps including the esterification and hydrolysis in the separation of the hydrogenation products). Among these crinine-type alkaloids, seven have been previously synthesized with 8–20 steps in 0.6–13.2% yields (see also ESI†).^7,8^ Eleven were synthesized for the first time: (+)- and (–)-8-*O*-demethylmaritidine, (+)- and (–)-siculine, (–)-maritidine, (–)-epimaritidine, (+)-powelline, (+)-epipowelline, (–)-buphanidrine, (–)-flexinine and (–)-augustine. The concise, practical strategy reported here for the synthesis of crinine-type alkaloids and analogues is expected to be applicable for the rapid syntheses of other types of Amaryllidaceae alkaloids or even other types of natural products.
