# Supplementary material for: Bioinspired enantioselective synthesis of crinine-type alkaloids via iridium-catalyzed asymmetric hydrogenation of enones
Source: Chem Sci. 2017 Jul 3;8(9):6202–6. doi: 10.1039/c7sc02112g (PMC5628337; doi:10.1039/c7sc02112g)
Supplement: Supplementary file 1 [file SC-008-C7SC02112G-s001.pdf]

Electronic Supplementary Material (ESI) for Chemical Science.
This journal is © The Royal Society of Chemistry 2017

Supporting Information

Bioinspired Enantioselective Synthesis of Crinine-type Alkaloids via Iridium-Catalyzed Asymmetric Hydrogenation of Enones

Xiao-Dong Zuo,[a] Shu-Min Guo,[a] Rui Yang,[a] Jian-Hua Xie*[a] and Qi-Lin Zhou*[ab]

[a] State Key Laboratory and Institute of Elemento-organic Chemistry, College of Chemistry, Nankai University, Tianjin 300071, China.

[b] Collaborative Innovation Center of Chemical Science and Engineering (Tianjin), Nankai University, Tianjin 300071, China.

General 1

(A) Preparation of Oxocrinines *rac*-1 2

(B) Asymmetric Hydrogenation of Oxocrinines *rac*-1 4

(C) Asymmetric Hydrogenation on Multigram-scale 10

(D) Asymmetric Synthesis of 8-*O*-Demethylmartidines, Siculines and Dihydrocrinine-type Alkaloids 11

(E) The Comparison of Our Results with Those Reported in Literatures 15

(F) NMR Spectra of rac-1e and Synthesised Crinine-type Alkaloids and Analogues 17

(G) HPLC Charts for Asymmetric Hydrogenation Products 38

General: All reactions and manipulations which are sensitive to moisture or air were performed in an argon-filled glovebox (VAC DRI-LAB HE 493) or using standard Schlenk techniques. Hydrogen gas (99.999%) was purchased from Boc Gas Inc., Tianjin. Anhydrous THF was distilled from sodium benzophenone ketyl. Anhydrous *n*PrOH, DCM and Et_3N were freshly distilled from calcium hydride. Anhydrous MeOH and EtOH were freshly distilled from magnesium. The chiral Ir-SpiroPAP catalysts (*R*)-**3** and (*S*)-**3** were available in our lab or purchased from Zhejiang Jiuzhou Pharmceutical Co., Ltd. All chemicals were purchased from J&K, Acros and Aldrich, and were used as received. Melting points were measured on a RY-I apparatus and uncorrected. ^{1}H and ^{13}C NMR spectra were recorded on Bruker AMX-400 spectrometers. Chemical shifts were reported in ppm downfield from internal $Si(CH_3)_4$ and external 85% H_3PO_4, respectively. Optical rotations were determined using a Perkin Elmer 341 polarimeter. HPLC analysis were performed using Hewlett Packard Model HP1100 instruments. HRMS were recorded on APEXII and ZAB-HS spectrometer.

(A) Preparation of Oxocrinines *rac*-**1**

Oxocrinines *rac*-**1** were prepared according to Node's procedure via a biomimetic intramolecular phenolic oxidative coupling as the key step.[1] Typical procedure for the synthesis of oxocrinine *rac*-**1a** was outlined below:

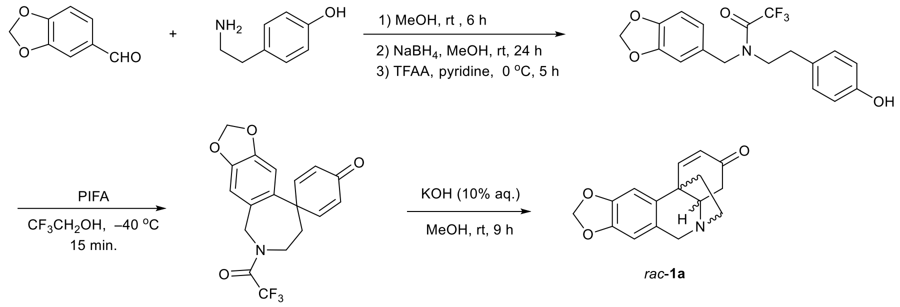

General procedure for the synthesis of oxocrinines: To a solution of piperonal (6.3 g, 42.0 mmol) in MeOH (50 mL) was added tyramine (6.0 g, 43.7 mmol), and the mixture was stirred at room temperature for 6 h. Sodium borohydride (1.6 g, 42.0 mmol) was added, and the mixture was stirred at room temperature for 24 h. The reaction mixture was then concentrated in vacuo. The residue was dissolved in $CHCl_3$ (100 mL), and washed with saturated brines, dried over anhydrous Na_2SO_4, and concentrated in vacuo to yield solid. The solid was dissolved in pyridine (115 mL) and trifluoroacetic anhydride (14.6 mL, 104.2 mmol) was added at 0 °C. The mixture was stirred at 0 °C for 5 h. The reaction was quenched with MeOH, concentrated in vacuo, and extracted with ethyl acetate (100 mL). The organic layer was washed with 1N HCl and brines, dried over anhydrous Na_2SO_4, and concentrated in vacuo to yield a brown viscous oil. The oil was purified by chromatography on silica gel column eluting with petroleum ether/ethyl acetate (2:1) to afford *N*-trifluoroacetyl protected amine (15.3 g, 100% yield) as a light yellow solid.

The solid (15.3 g, ~41.6 mmol) was dissolved with CF_3CH_2OH (150 mL) and the solution was cooled to −40 °C. The phenyliodine(III) bis(trifluoroacetate) (17.9 g, 41.6 mmol) in CF_3CH_2OH (100 mL) was slowly added to the solution at −40 °C. After addition, the mixture was stirred at −40 °C for 15 min, and concentrated in vacuo. The residue was purified by chromatography on silica gel column eluting with petroleum ether/ethyl acetate (1:1) to afford a dienone (11.2 g, 74% yield) as a white solid.

The white solid (11.2 g, 30.7 mmol) was dissolved with MeOH (150 mL), and 10% KOH solution (150 mL) was added at room temperature. The mixture was stirred at room temperature for 9 h, and concentrated in vacuo. The residue was redissolved with $CHCl_3$ (100 mL), washed with brines, dried over anhydrous Na_2SO_4, and concentrated in vacuo to yield a brown oil. The oil was purified by chromatography on silica gel column eluting with DCM/MeOH (20:1) to afford oxocrinine *rac*-**1a** (7.0 g, 86%) as a white solid. R_f = 0.5 (DCM/MeOH = 20:1), mp 170–172 °C, 64% overall yield (4 steps from piperonal). ^1H NMR (400 MHz, $CDCl_3$) δ 7.62 (d, J = 10.4 Hz, 1H), 6.91 (s, 1H), 6.52 (d, J = 10.0 Hz, 1H), 6.09 (d, J = 10.4 Hz, 1H), 5.93 (d, J = 4.0 Hz, 2H), 4.40 (d, J = 17.2 Hz, 1H), 3.80 (d, J = 17.2 Hz, 1H), 3.64 (dd, J = 13.2, 5.6 Hz, 1H), 3.57–3.50 (m, 1H), 3.05–2.97 (m, 1H), 2.71–2.66 (m, 1H), 2.51–2.34 (m, 2H), 2.20–2.13 (m, 1H).

Oxocrinine *rac*-1b[2]

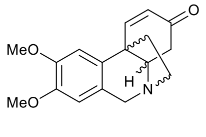

The racemic oxocrinine *rac*-**1b** was prepared by the same procedure as that for *rac*-**1a**. White solid, R_f = 0.5 (DCM/MeOH = 20:1), mp 144–146 °C, 4.2 g, 59% overall yield (4 steps from 3,4-dimethoxybenzaldehyde). ^1H NMR (400 MHz, $CDCl_3$) δ

7.70 (d, J = 10.4 Hz, 1H), 6.91 (s, 1H), 6.56 (s, 1H), 6.11 (d, J = 10.4 Hz, 1H), 4.44 (d, J = 16.8 Hz, 1H), 3.91 (s, 3H), 3.84 (d, J = 16.8 Hz, 1H), 3.84(s, 3H), 3.67 (dd, J = 12.8, 5.6 Hz, 1H), 3.60–3.53 (m, 1H), 3.07–3.30 (m, 1H), 2.71 (dd, J = 16.8, 5.6 Hz, 1H), 2.53–2.38 (m, 2H), 2.23–2.16 (m, 1H).

Oxocrinine *rac*-1c[3]

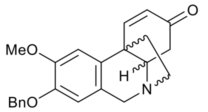

The racemic oxocrinine *rac*-**1c** was prepared by the same procedure as that for *rac*-**1a**. White solid, R_f = 0.4 (DCM/MeOH = 20:1), mp 147–149 °C, 3.6 g, 55% overall yield (4 steps from 4-(benzyloxy)-3-methoxybenzaldehyde). ^{1}H NMR (400 MHz, $CDCl_3$) δ 7.73 (d, J = 10.4 Hz, 1H), 7.44–7.29 (m, 5H), 6.94 (s, 1H), 6.59 (s, 1H), 6.12 (d, J = 10.4 Hz, 1H), 5.10 (s, 2H), 4.38 (d, J = 16.8 Hz, 1H), 3.92 (s, 3H), 3.78 (d, J = 16.8 Hz, 1H), 3.65 (dd, J = 12.8, 6.0 Hz, 1H), 3.58–3.51 (m, 1H), 3.05–2.98 (m, 1H), 2.72–2.66 (m, 1H), 2.52–2.37 (m, 2H), 2.22–2.15 (m, 1H).

Oxocrinine *rac*-1d[4]

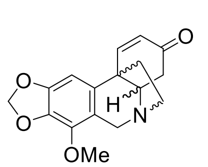

The racemic oxocrinine *rac*-**1d** was prepared by the same procedure as that for *rac*-**1a**. White solid, R_f = 0.5 (DCM/MeOH = 20:1), mp 179–181 °C, 1.9 g, 56% overall yield (4 steps from 7-methoxybenzo[*d*][1,3]dioxole-5-carbaldehyde). ^{1}H NMR (400 MHz, $CDCl_3$) δ 7.59 (d, J = 10.0 Hz, 1H), 6.63 (s, 1H), 6.07 (d, J = 10.0 Hz, 1H), 5.89 (d, J = 3.2 Hz, 2H), 4.20 (d, J = 17.6 Hz, 1H), 4.00 (s, 3H), 3.82 (d, J = 17.6 Hz, 1H), 3.61–3.49 (m, 2H), 3.02–2.95 (m, 1H), 2.69 (dd, J = 16.8, 5.6 Hz, 1H), 2.51–2.33 (m, 2H), 2.18–2.11 (m, 1H).

Oxocrinine *rac*-1e

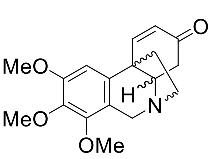

The racemic oxocrinine *rac*-**1e** was prepared by the same procedure as that for *rac*-**1a**. White solid, R_f = 0.6 (DCM/MeOH = 20:1), mp 144–146 °C, 2.6 g, 66% overall yield (4 steps from 3,4,5-trimethoxybenzaldehyde). ^{1}H NMR (400 MHz, $CDCl_3$) δ 8.52 (d, J = 10.4 Hz, 1H), 6.32 (s, 1H), 6.00 (d, J = 10.4 Hz, 1H), 4.40 (d, J = 16.8, 1H), 3.89 (s, 3H), 3.82 (s, 3H), 3.78 (s, 3H), 3.73 (d, J = 16.8 Hz, 1H), 3.62 (dd, J = 12.8, 5.6 Hz, 1H), 3.56–3.49 (m, 1H), 2.98–2.91 (m, 1H), 2.66–2.60 (m, 1H), 2.47–2.40 (m, 2H), 2.24–2.14 (m, 1H). ^{13}C NMR (100 MHz, $CDCl_3$) δ 198.2, 152.7, 152.4, 151.3, 141.2, 129.8, 128.3, 105.8, 69.8, 62.1, 60.9, 55.9, 54.2, 45.8, 44.4, 40.7. HRMS (ESI) Calcd for $C_{18}H_{22}NO_4$ ($[M + H]^+$): 316.1543, Found: 316.1540.

(B) Asymmetric Hydrogenation of Oxocrinines *rac*-1

Optimization of the reaction conditions

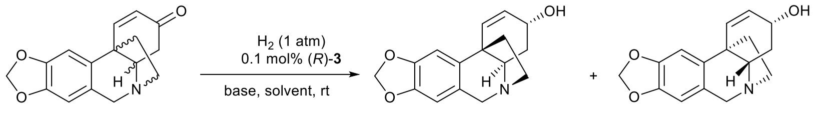

rac-**1a** *cis*-**2a** *trans*-**2a**

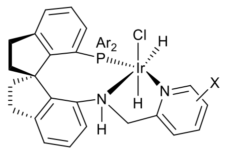

(*R*)-**3a**, Ar = C_6H_5, X = H
(*R*)-**3b**, Ar = 3,5-$(Me)_2C_6H_3$, X = H
(*R*)-**3c**, Ar = 3,5-$(tBu)_2C_6H_3$, X = H
(*R*)-**3d**, Ar = 3,5-$(tBu)_2C_6H_3$, X = 3-Me
(*R*)-**3e**, Ar = 3,5-$(tBu)_2C_6H_3$, X = 4-*t*Bu
(*R*)-**3f**, Ar = 3,5-$(tBu)_2C_6H_3$, X = 6-Me

(*R*)-**3** (Ir-SpiroPAP)

General procedure: To a Schlenk tube was added *rac*-**1a** (269 mg, 1 mmol), a solution of chiral iridium catalyst (*R*)-**3** in anhydrous EtOH (0.001 mmol/mL, 1.0 mL, 0.001 mmol), a solution of base in EtOH (0.05 mmol/mL, 1.0 mL, 0.05 mmol) and anhydrous solvent (4.0 mL) under nitrogen atmosphere. The flask was purged with hydrogen by using a balloon filled with hydrogen. The reaction mixture was stirred at 0 °C or room temperature under 1 atm of H_2 pressure until TLC showed that the *rac*-**1a** was entirely consumed. The reaction mixture was concentrated and passed through a short pad of silical gel eluting with DCM/MeOH (10:1) to give the yield of the mixture of *cis*-**2a** and *trans*-**2a**. The *cis*-**2a**/*trans*-**2a** ratio was analysed by ^{1}H NMR. The ee values of *cis*-**2a** and *trans*-**2a** were determined by chiral HPLC after converting into the benzoyl esters. A mixture of *cis*-**2a** and *trans*-**2a** (54 mg, 0.2 mmol) reacted with benzoyl chloride (141 mg, 1 mmol) in the presence of pyridine (111 mg, 1.4 mmol) in DCM (5 mL) by stirring at room temperature for 6 h. The reaction mixture was quenched with saturated NH_4Cl (5 mL) and extracted with DCM (3 × 5 mL). The combined organic extract was washed with brines, dried over anhydrous $MgSO_4$ and concentrated in vacuo. The residue was chromatographied on silica gel eluting with petroleum ether/ethyl acetate (1:4) to provide the desired benzoyl esters in nearly quantitative yields.

The catalyst, solvent, base and base concentration, reaction temperature, and hydrogen pressure were systematically investigated. The results were summarized in Table S1. The optimal reaction conditions are: 0.1 mol% (*R*)-**3d**, [*rac*-**1a**] = 0.17 M, [KO*t*Bu] = 0.008 M, 0 °C, 1 atm of H_2, EtOH/DCM (5:2) (Table S1, entry 16).

Table S1. Optimization of the reaction conditions for the asymmetric hydrogenation of *rac*-1a.[a]

Entry	(*R*)-3	Solvent	Base	Time (h)	Yield (%)[b]	*cis*/*trans*[c]	Ee (%) of **2a**[d]	
							cis	*trans*
1	(*R*)-**3a**	EtOH	KO*t*Bu	0.8	91	11:88	90	27
2	(*R*)-**3b**	EtOH	KO*t*Bu	0.5	89	15:85	88	23
3	(*R*)-**3c**	EtOH	KO*t*Bu	0.8	95	46:54	90	86
4	(*R*)-**3d**	EtOH	KO*t*Bu	0.5	93	45:55	97	87
5	(*R*)-**3e**	EtOH	KO*t*Bu	1.0	90	42:58	91	77
6	(*R*)-**3f**	EtOH	KO*t*Bu	0.3	95	36:64	94	64
7	(*R*)-**3d**	MeOH	KO*t*Bu	0.7	92	40:60	92	76
8	(*R*)-**3d**	*n*PrOH	KO*t*Bu	0.8	91	43:57	95	90
9	(*R*)-**3d**	EtOH	KOH	0.3	90	46:54	95	87
10	(*R*)-**3a**	EtOH	K_2CO_3	0.5	91	46:54	95	87
11	(*R*)-**3d**	EtOH	Et_3N	19	79	38:62	90	90

12[e]	(*R*)-**1d**	EtOH	KO*t*Bu	0.3	90	44:56	99	85
13[f]	(*R*)-**3d**	EtOH	KO*t*Bu	3	93	46:54	98	88
14[g]	(*R*)-**3d**	EtOH	KO*t*Bu	7.5	91	46:54	94	88
15[h]	(*R*)-**3d**	EtOH	KO*t*Bu	9	89	46:54	99	90
16[i]	(*R*)-**3d**	EtOH	KO*t*Bu	8.5	94	46:54	97	93

[a] Reaction conditions: 1 mmol scale, [**1a**] = 0.17 M, 0.1 mol % of catalyst, [base] = 0.017 M, solvent (6.0 mL), room temperature (22–27 °C), 100% conversions. [b] Isolated yield of the mixture of *cis*-**2a** and *trans*-**2a**. [c] The ratios of *cis*-**2a**/*trans*-**2a** were determined by ^{1}H NMR. [d] The ee values of *cis*-**2a** and *trans*-**2a** were determined by chiral HPLC (Chiralcel OD-3 column) after transforming into the benzoyl esters. [e] Under 5 atm of H_2. [f] At 0 °C. [g] At 0 °C, and [KO*t*Bu] = 0.034 M. [h] At 0 °C, and [KO*t*Bu] = 0.008 M. [i] At 0 °C, [KO*t*Bu] = 0.008 M, and DCM as co-solvent (ethanol/DCM = 5:2).

Asymmetric hydrogenation of oxocrinines *rac*-1

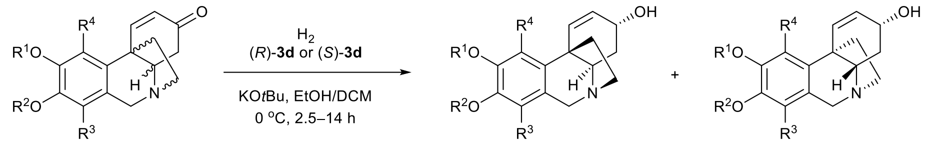

rac-**1** *cis*-**2** *trans*-**2**

General procedure: To a Schlenk tube was added *rac*-**1a** (269 mg, 1 mmol), a solution of chiral iridium catalyst (*R*)-**3d** in anhydrous EtOH (0.001 mmol/mL, 1.0 mL, 0.001 mmol), a solution of KO*t*Bu in EtOH (0.05 mmol/mL, 1.0 mL, 0.05 mmol), anhydrous EtOH (2.3 mL), and DCM (1.7 mL). The Schlenk tube was purged with hydrogen by using a balloon filled with hydrogen, and the mixture was stirred at 0 °C until TLC showed that the *rac*-**1a** was completely consumed. The reaction mixture was concentrated and passed through a short pad of silical gel eluting with DCM/MeOH (10:1) to give a mixture of *cis*-**2a** and *trans*-**2a**. The *cis*-**2a**/*trans*-**2a** ratio was analysed by ^{1}H NMR. The ee values of *cis*-**2a** and *trans*-**2a** were determined by chiral HPLC after converting into the benzoyl esters. The mixture of *cis*-**2a** and *trans*-**2a** was added DCM (25 mL), benzoyl chloride (702 mg, 5 mmol), pyridine (553 mg, 7 mmol), and stirred at room temperature for 6 h. The reaction was quenched with saturated NH_4Cl (15 mL), and extracted with DCM (3 × 20 mL). The combined organic extract was washed with brines, dried over anhydrous $MgSO_4$ and concentrated in vacuo. The residue was chromatographied on silica gel eluting with petroleum ether/ethyl acetate (1:4) to give benzoyl esters as a white solid. The benzoyl esters were separated and measured ee values by chiral HPLC (Chiralcel OD-3 column): 97% ee for the benzoyl ester of ***cis*-2a** and 93% ee for the benzoyl ester of *trans*-**2a**. The benzoyl ester of ***cis*-2a** was hydrolysed with KOH (10%, 10 mL) in mixed solvent of MeOH (10 mL) and THF (10 mL). The mixture was heated at 50 °C with vigorous stirring for 15 h, and concentrated in vacuo. The residue was extracted with $CHCl_3$ (3 × 15 mL), washed with brines, dried over $MgSO_4$, and chromatographied on silica gel column eluting with DCM/MeOH (10:1) to afford *cis*-**2a** (103 mg, 38% yield) as white solid. Using the same procedure, the hydrolysis of the benzoyl ester of *trans*-**2a** gave *trans*-**2a** (124 mg, 46% yield) as white solid.

(–)-Crinine ((–)-*cis*-2a)[5]

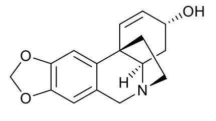

(*R*)-**3d**, 8.5 h, 103 mg, 38% yield, 97% ee, R_f = 0.4 (DCM/MeOH = 5:1), white solid, mp 208–210 °C, $[\alpha]_D^{20}$ –21.0 (*c* 0.5, $CHCl_3$) [natural (–)-crinine:[6] $[\alpha]_D^{20}$ –23 (*c* 0.5, $CHCl_3$)]. ^{1}H NMR (400 MHz, $CDCl_3$) δ 6.88 (s, 1H), 6.55 (s, 1H), 6.44 (d, *J* = 10.0 Hz, 1H), 6.07 (dd, *J* = 10.0, 5.1 Hz, 1H), 5.96 (d, *J* = 2.9 Hz, 2H), 4.75 (d, *J* = 16.0 Hz, 1H), 4.48 (brs, 1H), 4.10 (d, *J* = 16.0 Hz, 1H), 3.95–3.91 (m, 2H), 3.20–3.17 (m, 1H), 2.69–2.65 (m, 1H),

2.37–2.31 (m, 1H), 2.21–2.13 (m, 1H), 1.91–1.83 (m, 1H). ^{13}C NMR (100 MHz, $CDCl_3$) δ 147.5, 146.9, 135.5, 129.6, 127.8, 120.0, 107.3, 103.4, 101.5, 64.4, 61.9, 59.8, 52.8, 44.8, 41.5, 30.8. HPLC conditions: Chiralcel OD-3 column (25 cm × 0.46 cm ID); *n*-hexane/2-propanol = 85:15; temp, rt; flow rate = 1.0 mL/min; 210 nm UV detector; t_R (major) = 9.58 min and t_R (minor) = 13.18 min.

(+)-Epivittatine ((+)-*trans*-2a)[7]

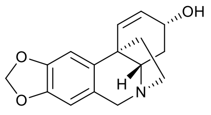

(*R*)-**3d**, 8.5 h, 124 mg, 46% yield, 93% ee, R_f = 0.4 (DCM/MeOH = 5:1), white solid, mp 233–234 °C, $[\alpha]^{22}_{D}$ +96.4 (*c* 0.25, EtOH) [natural (+)-epivittatine:[7] $[\alpha]^{22}_{D}$ +102 (*c* 0.25, EtOH)]. ^{1}H NMR (400 MHz, $CDCl_3$) δ 6.82 (s, 1H), 6.50 (s, 1H), 6.41 (dd, *J* = 10.2, 2.4 Hz, 1H), 5.90 (d, *J* = 1.2 Hz, 2H), 5.80 (d, *J* = 10.2 Hz, 1H), 4.48–4.44 (m, 1H), 4.40 (d, *J* = 17.2 Hz, 1H), 3.78 (d, *J* = 17.2 Hz, 1H), 3.48–3.41 (m, 1H), 3.26 (dd, *J* = 13.2, 3.6 Hz, 1H), 2.96–2.89 (m, 1H), 2.23–2.07 (m, 3H), 1.69–1.60 (m, 1H). ^{13}C NMR (100 MHz, $CDCl_3$) δ 146.3, 145.9, 138.7, 131.9, 128.6, 125.8, 107.0, 102.9, 100.9, 67.4, 66.7, 62.1, 53.3, 45.1, 44.5, 35.1. HPLC conditions: Chiralcel OD-3 column (25 cm × 0.46 cm ID); *n*-hexane/2-propanol = 85:15; temp, rt; flow rate = 1.0 mL/min; 210 nm UV detector; t_R (major) = 12.18 min and t_R (minor) = 19.26 min.

(+)-Vittatine ((+)-*cis*-2a)[8]

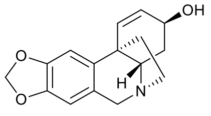

(*S*)-**3d**, 8.5 h, 106 mg, 39% yield, 97% ee, R_f = 0.4 (DCM/MeOH = 5:1), white solid, mp 209–211 °C, $[\alpha]^{25}_{D}$ +23.0 (*c* 0.5, $CHCl_3$) [natural (+)-vittatine:[9] $[\alpha]^{25}_{D}$ +26 (*c* 0.5, $CHCl_3$)]. HPLC conditions: Chiralcel OD-3 column (25 cm × 0.46 cm ID); *n*-hexane/2-propanol = 85:15; temp, rt; flow rate = 1.0 mL/min; 210 nm UV detector; t_R (minor) = 10.04 min and t_R (major) = 13.41 min. The NMR spectra of (+)-*cis*-**2a** are same as those of (−)-*cis*-**2a**.

(−)-Epicrinine ((−)-*trans*-2a)[7]

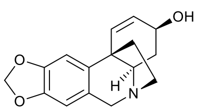

(*S*)-**3d**, 8.5 h, 119 mg, 44% yield, 93% ee, R_f = 0.4 (DCM/MeOH = 5:1), white solid, mp 237–239 °C, $[\alpha]^{27}_{D}$ −139 (*c* 1.0, $CHCl_3$) [lit.:[10] $[\alpha]^{27}_{D}$ −142 (*c* 1.0, $CHCl_3$)]. HPLC conditions: Chiralcel OD-3 column (25 cm × 0.46 cm ID); *n*-hexane/2-propanol = 85:15; temp, rt; flow rate = 1.0 mL/min; 210 nm UV detector; t_R (minor) = 12.25 min and t_R (major) = 18.93 min. The NMR spectra of (−)-*trans*-**2a** are same as those of (+)-*trans*-**2a**.

(−)-Maritidine ((−)-*cis*-2b)[11]

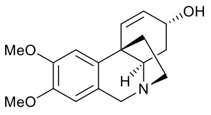

(*R*)-**3d**, 13 h, 100 mg, 35% yield, 96% ee, R_f = 0.4 (DCM/MeOH = 5:1), white solid, mp 254–255 °C, $[\alpha]^{22}_{D}$ −21.4 (*c* 0.78, $CHCl_3$). ^{1}H NMR (400 MHz, $CDCl_3$) δ 6.85 (s, 1H), 6.64 (d, *J* = 10.0 Hz, 1H), 6.52 (s, 1H), 6.00 (dd, *J* = 9.8, 5.0 Hz, 1H), 4.48 (d, *J* = 16.8 Hz, 1H), 4.38–4.37 (m, 1H), 3.88 (s, 3H), 3.86 (d, *J* = 16.8 Hz, 1H), 3.82 (s, 3H), 3.52–3.43 (m, 2H), 2.99–2.92 (m, 1H), 2.25–2.19 (m, 1H), 2.11–2.08 (m, 1H), 2.01–1.93 (m, 1H), 1.81–1.73 (m, 1H). ^{13}C NMR (100 MHz, $CDCl_3$) δ 147.8, 147.6, 136.8, 131.8, 127.9, 124.3, 110.0, 106.0, 64.0, 63.3, 61.9, 56.2, 56.0, 53.6, 44.2, 43.9, 32.6. HPLC conditions: Chiralcel OD-3 column (25 cm × 0.46 cm ID); *n*-hexane/2-propanol = 75:25; temp, rt; flow rate = 1.0 mL/min; 210 nm UV detector; t_R (major) = 7.83 min and t_R (minor) = 9.28 min.

(+)-Epimaritidine ((+)-*trans*-2b)[11]

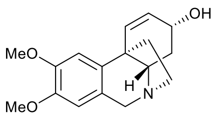

(R)-**3d**, 13 h, 123 mg, 43% yield, 93% ee, R_f = 0.4 (DCM/MeOH = 5:1), white solid, mp 210–211 ℃, $[\alpha]_D^{22}$ +80.0 (c 0.47, MeOH) [natural (+)-epimaritidine:[11] $[\alpha]_D^{22}$ +83.2 (c 0.47, MeOH)]. ^{1}H NMR (400 MHz, $CDCl_3$) δ 6.78 (s, 1H), 6.50 (s, 1H), 6.46 (dd, J = 10.4, 1.6 Hz, 1H), 5.79 (d, J = 10.0 Hz, 1H), 4.43–4.39 (m, 2H), 3.90 (brs, 1H), 3.86 (s, 3H), 3.81–3.77 (m, 4H), 3.47–3.40 (m, 1H), 3.25 (dd, J = 13.2, 3.2 Hz, 1H), 2.95–2.88 (m, 1H), 2.22–2.05 (m, 3H), 1.67–1.59 (m, 1H). ^{13}C NMR (100 MHz, $CDCl_3$) δ 147.5, 147.5, 137.6, 131.9, 128.8, 124.8, 110.0, 105.7, 67.6, 66.9, 61.9, 56.1, 56.0, 53.4, 45.2, 44.2, 35.2. HPLC conditions: Chiralcel OD-3 column (25 cm × 0.46 cm ID); n-hexane/2-propanol = 75:25; temp, rt; flow rate = 1.0 mL/min; 210 nm UV detector; t_R (major) = 10.90 min and t_R (minor) = 16.78 min.

(+)-Maritidine ((+)-*cis*-2b)[11]

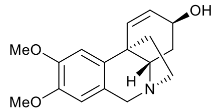

(S)-**3d**, 11 h, 106 mg, 37% yield, 96% ee, R_f = 0.4 (DCM/MeOH = 5:1), white solid, mp 247–249 ℃, $[\alpha]_D^{22}$ +20.3 (c 0.78, $CHCl_3$). [natural (+)-maritidine:[11] $[\alpha]_D^{22}$ +22.4 (c 0.78, $CHCl_3$)]. HPLC conditions: Chiralcel OD-3 column (25 cm × 0.46 cm ID); n-hexane/2-propanol = 75:25; temp, rt; flow rate =1.0 mL/min; 210 nm UV detector; t_R (minor) = 8.02 min and t_R (major) = 9.23 min. The NMR spectra of (+)-*cis*-**2b** are same as those of (–)-*cis*-**2b**.

(–)-Epimaritidine ((–)-*trans*-2b)[11]

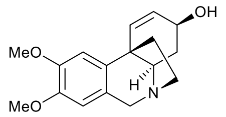

(S)-**3d**, 11 h, 132 mg, 46% yield, 93% ee, R_f = 0.4 (DCM/MeOH = 5:1), white solid, mp 212–214 °C, $[\alpha]_D^{22}$ –79.6 (c 0.47, MeOH). HPLC conditions: Chiralcel OD-3 column (25 cm × 0.46 cm ID); n-hexane/2-propanol = 75:25; temp, rt; flow rate = 1.0 mL/min; 210 nm UV detector; t_R (minor) = 10.95 min and t_R (major) = 16.43 min. The NMR spectra of (–)-*trans*-**2b** are same as those of (+)-*trans*-**2b**.

(–)-*cis*-2c

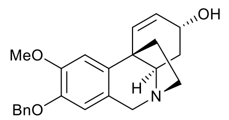

(R)-**3d**, 2.5 h, 138 mg, 38% yield, 98% ee, R_f = 0.3 (DCM/MeOH = 5:1), semi-oil, $[\alpha]_D^{27}$ –11.8 (c 0.5, $CHCl_3$). ^{1}H NMR (400 MHz, $CDCl_3$) δ 7.43–7.27 (m, 5H), 6.83 (s, 1H), 6.54 (s, 1H), 6.49–6.46 (m, 1H), 5.80 (d, J = 10.0 Hz, 1H), 5.07 (s, 2H), 4.44–4.41 (m, 1H), 4.36 (d, J = 16.8 Hz, 1H), 3.88 (s, 3H), 3.73 (d, J = 16.8 Hz, 1H), 3.46–3.39 (m, 1H), 3.27–3.23 (m, 1H), 2.93–2.87 (m, 1H), 2.23–2.06 (m, 3H), 1.68–1.59 (m, 1H). ^{13}C NMR (100 MHz, $CDCl_3$) δ 148.2, 146.8, 138.2, 137.2, 132.0, 128.6, 128.5, 127.9, 127.3, 124.6, 112.7, 106.4, 71.2, 67.3, 66.8, 61.7, 56.4, 53.2, 45.0, 44.3, 35.0. HRMS (ESI) Calcd for $C_{23}H_{26}NO_3$ ($[M + H]^+$): 364.1907, Found: 364.1912. HPLC conditions: Chiralcel OD-3 column (25 cm × 0.46 cm ID); n-hexane/2-propanol = 75:25; temp, rt; flow rate = 1.0 mL/min; 210 nm UV detector; t_R (major) = 13.93 min and t_R (minor) = 16.22 min.

(+)-*trans*-2c

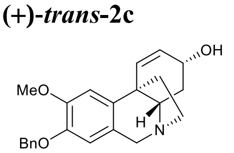

(R)-**3d**, 2.5 h, 156 mg, 43% yield, 93% ee, R_f = 0.3 (DCM/MeOH = 5:1), semi-oil, $[\alpha]_D^{24}$ +86.6 (c 0.25, $CHCl_3$). ^{1}H NMR (400 MHz, $CDCl_3$) δ 7.43–7.28 (m, 5H), 6.83 (s, 1H), 6.55 (s, 1H), 6.47 (d, J = 9.6 Hz, 1H), 5.81 (d, J = 10.4 Hz, 1H), 5.08 (s, 2H), 4.48–4.38 (m, 2H), 3.89 (s, 3H), 3.78 (d, J = 16.8 Hz, 1H), 3.51–3.46 (m, 1H), 3.30–3.27 (m, 1H), 2.97–2.90 (m, 1H), 2.22–2.12 (m, 3H), 1.70–1.61 (m, 1H). ^{13}C NMR (101 MHz, $CDCl_3$) δ 148.3, 146.9, 137.9, 137.2, 132.0, 128.7, 128.5, 128.0, 127.4, 124.2, 112.7, 106.5, 71.3, 67.5, 66.9, 61.6, 56.4, 53.3, 44.8, 44.3, 34.9. HRMS (ESI) Calcd for $C_{23}H_{26}NO_3$ ($[M + H]^+$): 364.1907, Found: 364.1902.

HPLC conditions: Chiralcel OD-3 column (25 cm × 0.46 cm ID); *n*-hexane/2-propanol = 75:25; temp, rt; flow rate = 1.0 mL/min; 230 nm UV detector; t_R (major) = 14.00 min and t_R (minor) = 24.25 min.

(+)-*cis*-2c

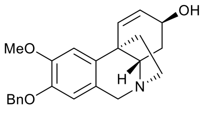

(*S*)-**3d**, 2.5 h, 134 mg, 37% yield, 98% ee, R_f = 0.3 (DCM/MeOH = 5:1), semi-oil, $[\alpha]_D^{27}$ +12.1 (*c* 0.5, $CHCl_3$). HPLC conditions: Chiralcel OD-3 column (25 cm × 0.46 cm ID); *n*-hexane/2-propanol = 75:25; temp, rt; flow rate =1.0 mL/min; 210 nm UV detector; t_R (minor) = 14.08 min and t_R (major) = 16.06 min. The NMR spectra of (+)-*cis*-**2c** are same as those of (–)-*cis*-**2c**.

(–)-*trans*-2c

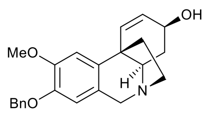

(*S*)-**3d**, 2.5 h, 148 mg, 41% yield, 93% ee, R_f = 0.3 (DCM/MeOH = 5:1), semi-oil, $[\alpha]_D^{24}$ –82.5 (*c* 0.25, $CHCl_3$). HPLC conditions: Chiralcel OD-3 column (25 cm × 0.46 cm ID); *n*-hexane/2-propanol = 75:25; temp, rt; flow rate = 1.0 mL/min; 230 nm UV detector; t_R (minor) = 14.21 min and t_R (major) = 23.99 min. The NMR spectra of (–)-*trans*-**2c** are same as those of (+)-*trans*-**2c**.

(–)-Powelline ((–)-*cis*-2d)[4]

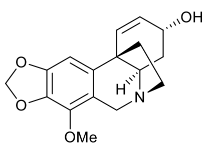

(*R*)-**3d**, 13 h, 105 mg, 35% yield, 96% ee, R_f = 0.4 (DCM/MeOH = 5:1), white solid, mp 197–199 °C, $[\alpha]_D^{22}$ –1.8 (*c* 0.5, $CHCl_3$). [natural (–)-powelline[10]: $[\alpha]_D^{25}$ 0 (*c* 0.5, $CHCl_3$)]. ^{1}H NMR (400 MHz, $CDCl_3$) δ 6.56 (s, 1H), 6.52 (d, *J* = 10.0 Hz, 1H), 5.93 (dd, *J* = 9.6, 5.2 Hz, 1H), 5.84 (d, *J* = 7.2 Hz, 2H), 4.34–4.31 (m, 1H), 4.23 (d, *J* = 17.6 Hz, 1H), 3.95 (s, 3H), 3.79 (d, *J* = 17.6 Hz, 1H), 3.32–3.38 (m, 2H), 2.89–2.82 (m, 1H), 2.18–2.12 (m, 2H), 2.03–2.00 (m, 1H), 1.92–1.85 (m, 1H). 1.76–1.68 (m, 1H). ^{13}C NMR (100 MHz, $CDCl_3$) δ 148.2, 141.1, 139.3, 133.5, 132.1, 127.7, 117.2, 100.7, 96.9, 64.0, 62.6, 59.3, 58.7, 53.8, 44.3, 44.1, 32.8. HPLC conditions: Chiralcel OD-3 column (25 cm × 0.46 cm ID); *n*-hexane/2-propanol = 85:15; temp, rt; flow rate = 1.0 mL/min; 210 nm UV detector; t_R (major) = 9.14 min and t_R (minor) = 10.10 min.

(+)-Epipowelline ((+)-*trans*-2d)[4]

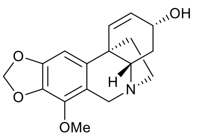

(*R*)-**3d**, 13 h, 117 mg, 39% yield, 89% ee, R_f = 0.4 (DCM/MeOH = 5:1), white solid, mp 170–171 ℃, $[\alpha]_D^{26}$ +106 (*c* 1.2, $CHCl_3$). ^{1}H NMR (400 MHz, $CDCl_3$) δ 6.51 (s, 1H), 6.32 (d, *J* = 10.0 Hz, 1H), 5.84 (d, *J* = 6.0 Hz, 2H), 5.78 (d, *J* = 10.4 Hz, 1H), 4.61 (brs, 1H), 4.42–4.39 (m, 1H), 4.24 (d, *J* = 17.6 Hz, 1H), 3.96 (s, 3H), 3.80 (d, *J* = 17.6 Hz, 1H), 3.50–3.43 (m, 1H), 3.24–3.20 (m, 1H), 2.94–2.87 (m, 1H), 2.26–2.06 (m, 3H), 1.68–1.59 (m, 1H). ^{13}C NMR (100 MHz, $CDCl_3$): δ 148.4, 140.9, 139.6, 133.5, 132.2, 128.1, 116.0, 100.7, 96.8, 67.1, 66.4, 59.2, 58.2, 53.3, 44.5, 44.5, 34.6. HPLC conditions: Chiralcel OD-3 column (25 cm × 0.46 cm ID); *n*-hexane/2-propanol = 85:15; temp, rt; flow rate = 1.0 mL/min; 230 nm UV detector; t_R (major) = 13.57 min and t_R (minor) = 16.80 min.

(+)-Powelline ((+)-*cis*-2d)[4]

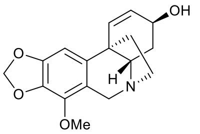

(*S*)-**3d**, 12 h, 108 mg, 36% yield, 97% ee, R_f = 0.4 (DCM/MeOH = 5:1), white solid, mp 196–198 ℃ decomposition, $[\alpha]_D^{22}$ +1.6 (*c* 0.5, $CHCl_3$). HPLC conditions: Chiralcel OD-3 column (25 cm × 0.46 cm ID); *n*-hexane/2-propanol = 85:15; temp,

rt; flow rate =1.0 mL/min; 210 nm UV detector; t_R (minor) = 9.31 min and t_R (major) =10.11 min. The NMR spectra of (+)-*cis*-**2d** are same as those of (–)-*cis*-**2d**.

(–)-Epipowelline ((–)-*trans*-2d)[4]

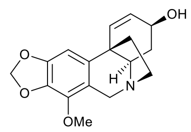

(*S*)-**3d**, 12 h, 120 mg, 40% yield, 90% ee, R_f = 0.4 (DCM/MeOH = 5:1), white solid, mp 171–172 °C, $[\alpha]_D^{26}$ –96.2 (*c* 1.2, $CHCl_3$). [lit.[10]: $[\alpha]_D^{26}$ –103 (*c* 1.2 $CHCl_3$)]. HPLC conditions: Chiralcel OD-3 column (25 cm × 0.46 cm ID); n-hexane/2-propanol = 85:15; temp, rt; flow rate = 1.0 mL/min; 230 nm UV detector; t_R (minor) = 13.70 min and t_R (major) = 16.48 min. The NMR spectra of (–)-*trans*-**2d** are same as those of (+)-*trans*-**2d**.

(–)-*cis*-2e

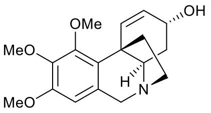

(*R*)-**3d**, 13 h, 130 mg, 41% yield, 86% ee, R_f = 0.5 (DCM/MeOH = 5:1), white solid, mp 160–162 °C, $[\alpha]_D^{27}$ –40.7 (*c* 0.5, $CHCl_3$). ^{1}H NMR (400 MHz, $CDCl_3$) δ 7.29 (d, *J* = 10.0 Hz, 1H), 6.29 (s, 1H), 5.90 (dd, *J* = 10.0, 5.2 Hz, 1H), 4.50 (d, *J* = 16.4. Hz, 1H), 4.30 (brs, 1H), 3.86–3.78 (m, 10H), 3.60–3.55 (m, 1H), 3.51–3.46 (m, 1H), 2.94–2.87 (m, 1H), 2.35–2.30 (m, 1H), 2.16–2.13 (m, 1H), 2.07–1.99 (m, 1H), 1.78–1.71 (m, 1H). ^{13}C NMR (100 MHz, $CDCl_3$) δ 152.2, 151.4, 141.5, 133.6, 130.0, 128.2, 127.0, 105.8, 63.8, 63.3, 62.1, 61.0, 60.9, 56.0, 53.5, 45.7, 43.4, 32.8. HRMS (ESI) Calcd for $C_{18}H_{24}NO_4$ ($[M + H]^+$): 318.1700, Found: 318.1706. HPLC conditions: Chiralcel OD-3 column (25 cm × 0.46 cm ID); *n*-hexane/2-propanol = 90:10; temp, rt; flow rate = 1.0 mL/min; 230 nm UV detector; t_R (major) = 13.72 min and t_R (minor) = 15.26 min.

(+)-*trans*-2e

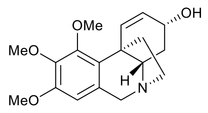

(*R*)-**3d**, 13 h, 120 mg, 38% yield, 87% ee, R_f = 0.5 (DCM/MeOH = 5:1), white foam, $[\alpha]_D^{27}$ +78.4 (*c* 0.5, $CHCl_3$). ^{1}H NMR (400 MHz, $CDCl_3$) δ 7.07 (dd, *J* = 10.4, 2.0 Hz, 1H), 6.30 (s, 1H), 5.68 (d, *J* = 10.4 Hz, 1H), 4.42 (d, *J* = 17.2 Hz, 1H), 4.42–4.38 (m, 1H), 3.82–3.78 (m, 10H), 3.73 (d, *J* = 17.2 Hz, 1H), 3.45–3.38 (m, 1H), 3.26 (dd, *J* = 13.2, 3.6 Hz, 1H), 2.89–2.82 (m, 1H), 2.32–2.26 (m, 1H), 2.16–2.09 (m, 1H), 1.62–1.53 (m, 1H). ^{13}C NMR (100 MHz, $CDCl_3$) δ 151.9, 151.0, 141.2, 131.1, 131.1, 130.9, 129.4, 105.8, 67.5, 67.0, 62.5, 60.9, 60.9, 56.0, 53.3, 45.5, 45.1, 35.7. HRMS (ESI) Calcd for $C_{18}H_{24}NO_4$ ($[M + H]^+$): 318.1700, Found: 318.1703. HPLC conditions: Chiralcel OD-3 column (25 cm × 0.46 cm ID); *n*-hexane/2-propanol = 85:15; temp, rt; flow rate = 1.0 mL/min; 230 nm UV detector; t_R (major) = 9.18 min and t_R (minor) = 15.15 min.

(+)-*cis*-2e

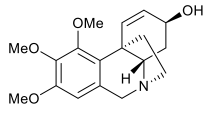

(*S*)-**3d**, 14 h, 126 mg, 40% yield, 87% ee, R_f = 0.5 (DCM/MeOH = 5:1), white solid, mp 159–160 ℃, $[\alpha]_D^{27}$ +41.3 (*c* 0.5, $CHCl_3$). HPLC conditions: Chiralcel OD-3 column (25 cm × 0.46 cm ID); *n*-hexane/2-propanol = 90:10; temp, rt; flow rate =1.0 mL/min; 230 nm UV detector; t_R (minor) = 13.65 min and t_R (major) =15.93 min. The NMR spectra of (+)-*cis*-**2e** are same as those of (–)-*cis*-**2e**.

(–)-*trans*-2e

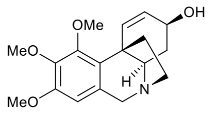

(*S*)-**3d**, 14 h, 130 mg, 41% yield, 88% ee, R_f = 0.5 (DCM/MeOH = 5:1), white foam, $[\alpha]_D^{27}$ –79.7 (*c* 0.5, $CHCl_3$). HPLC conditions: Chiralcel OD-3 column (25 cm × 0.46 cm ID); *n*-hexane/2-propanol = 85:15; temp, rt; flow rate = 1.0 mL/min; 230 nm UV detector; t_R (minor) = 9.24 min and t_R (major) = 14.88 min. The NMR spectra of (–)-*trans*-

2e are same as those of (+)-*trans*-**2e**.

(C) Asymmetric Hydrogenation on Multigram-scale.

Asymmetric hydrogenation of *rac*-1a on multigram-scale.

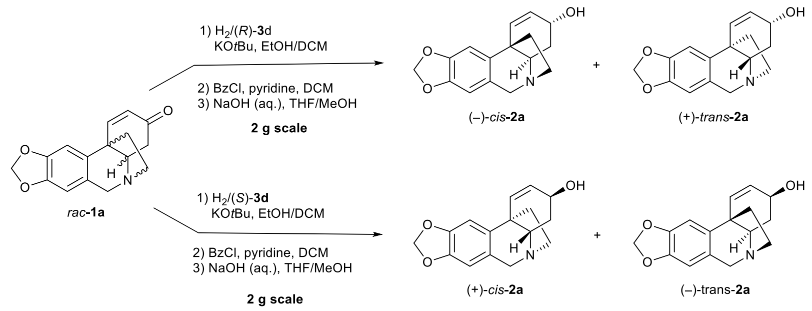

According to the general procedure for the asymmetric hydrogenation of *rac*-**1**, the asymmetric hydrogenations of *rac*-**1a** on a 2.0 g-scale with (*R*)-**3d** or (*S*)-**3d** provided the (–)-*cis*-**2a** and (+)-*trans*-**2a** or their enantiomers (+)-*cis*-**2a** and (–)-*trans*-**2a** in high yields and enantioselectivities. (Reaction conditions: *rac*-**1a** (2.0 g, 7.4 momol), (*R*)-**3d** or (*S*)-**3d** (7.4 mg, 0.0074 mmol), KO*t*Bu (31.4 mg, 0.37 mmol), DCM (9.5 mL), EtOH (23.5 mL), under ambient hydrogen pressure (balloon conditions) and 8–10 °C for 9 h).

(–)-*cis*-**2a:** 740 mg, 37% yield, 97% ee.
(+)-*trans*-**2a:** 879 mg, 44% yield, 92% ee.
(+)-*cis*-**2a:** 739 mg, 37% yield, 98% ee.
(–)-*trans*-**2a:** 899 mg, 45% yield, 94% ee.

Asymmetric hydrogenation of *rac*-1c on multigram-scale.

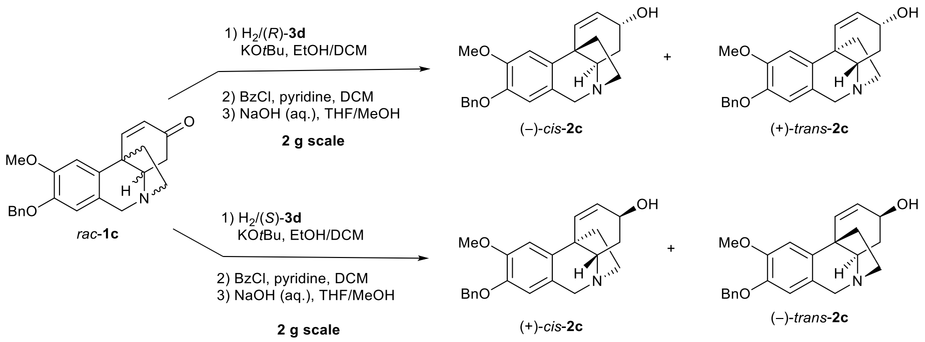

According to the general procedure for the asymmetric hydrogenation of *rac*-**1**, the asymmetric hydrogenations of *rac*-**1c** on a 2.0 g-scale with (*R*)-**3d** or (*S*)-**3d** provided the (–)-*cis*-**2c** and (+)-*trans*-**2c** or their enantiomers (+)-*cis*-**2c** and (–)-*trans*-**2c** in high yields and enantioselectivities. (Reaction conditions: *rac*-**1c** (2.0 g, 5.5 momol), (*R*)-**3d** or (*S*)-**3d** (5.4 mg, 0.0055 mmol), KO*t*Bu (30.8 mg, 0.28 mmol), DCM (9.5 mL), EtOH (23.5 mL), under ambient hydrogen pressure (balloon conditions) and 8–10 °C for 3 h).

(–)-*cis*-**2c:** 780 mg, 39% yield, 98% ee.
(+)-*trans*-**2c:** 839 mg, 42% yield, 93% ee.

(+)-*cis*-**2c:** 739 mg, 37% yield, 98% ee.
(−)-*trans*-**2c:** 799 mg, 40% yield, 93% ee.

(D) Asymmetric Synthesis of 8-*O*-Demethylmaritidines, Siculines and Dihydrocrinine-type Alkaloids.

Asymmetric synthesis of dihydrocrinine-type alkaloids

(−)-Dihydrocrinine[12]

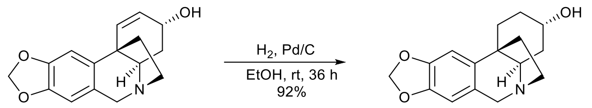

(−)-*cis*-**2a** (−)-dihydrocrinine

General procedure: To a solution of (−)-*cis*-**2a** (27mg, 0.1 mmol) in anhydrous EtOH (5 mL) was added palladium on charcoal (10%, 11 mg). The mixture was stirred under ambient hydrogen pressure (balloon condition) at room temperature for 36 h. The catalyst was removed by filtration, and the filtrate was concentrated in vacuo. The residue was purified by chromatography on silica gel column eluting with DCM/MeOH (8:1) to afford (−)–dihydrocrinine as a white solid. 25 mg, 92% yield, R_f = 0.3 (DCM/MeOH = 5:1), white solid, mp 211–212 °C, $[\alpha]_D^{26}$ −30.0 (*c* 0.7, $CHCl_3$) [lit:[13] $[\alpha]_D$ −21.0 (*c* 0.7, $CHCl_3$)]. ^1H NMR (400 MHz, $CDCl_3$) δ 6.71 (s, 1H), 6.41 (s, 1H), 5.87 (s, 2H), 4.35 (d, *J* = 16.4 Hz, 1H), 4.20 (brs, 1H), 3.81–3.71 (m, 2H), 3.36–3.30 (m, 2H), 2.85–2.81 (m, 1H), 2.22–1.95 (m, 4H), 1.84–1.66 (m, 3H), 1.43–1.36 (m, 1H). ^{13}C NMR (100 MHz, $CDCl_3$) δ 146.4, 145.8, 141.1, 124.8, 106.2, 103.4, 100.8, 65.7, 62.9, 61.6, 51.9, 42.5, 37.2, 33.2, 27.8, 22.5.

(+)-Dihydrovittatine[12]

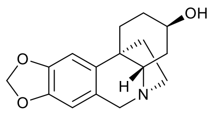

Using (+)-*cis*-**2a** (27 mg, 0.1 mmol), the (+)-dihydrovittatine was prepared (23 mg, 89% yield), R_f = 0.3 (DCM/MeOH = 5:1), white solid, mp 215–216 °C, $[\alpha]_D^{26}$ +22.0 (*c* 0.7, $CHCl_3$) [lit:[13] $[\alpha]_D$ +24.0 (*c* 0.7, $CHCl_3$)]. The NMR spectra of (+)-dihydrovittatine are same as (−)-dihydrocrinine.

(+)-Dihydroepivittatine[14]

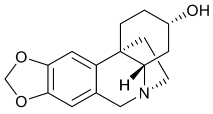

Using (+)-*trans*-**2a** (27 mg, 0.1 mmol), the (+)-dihydroepivittatine was prepared (26 mg, 94% yield), R_f = 0.3 (DCM/MeOH = 5:1), white solid, mp 101–102 °C, $[\alpha]_D^{26}$ + 10.5 (*c* 0.7, $CHCl_3$) [lit:[13] $[\alpha]_D$ +12.3 (*c* 0.7, $CHCl_3$)]. ^1H NMR (400 MHz, $CDCl_3$) δ 6.69 (s, 1H), 6.47 (s, 1H), 5.89 (s, 2H), 4.56 (brs, 1H), 4.45 (d, *J* = 16.4 Hz, 1H), 3.85 (d, *J* = 16.4 Hz, 1H), 3.65–3.51 (m, 2H), 3.10 (dd, *J* = 12.4, 5.2 Hz, 1H), 2.96–2.89 (m, 1H), 2.39–2.21 (m, 3H), 2.04–1.98 (m, 1H), 1.82–1.53 (m, 3H), 1.44–1.35 (m, 1H). ^{13}C NMR (100 MHz, $CDCl_3$) δ 146.8, 146.2, 140.5, 124.3, 106.4, 103.6, 101.0, 68.5, 66.8, 61.6, 52.0, 42.5, 37.4, 36.3, 30.7, 26.6.

(−)-Dihydroepicrinine[14]

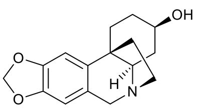

Using (−)-*trans*-**2a** (27 mg, 0.1 mmol), the (−)-dihydroepicrinine was prepared (24 mg, 90% yield), R_f = 0.3 (DCM/MeOH = 5:1), white solid, mp 99–100 °C, $[\alpha]_D^{26}$ −11.2 (*c* 0.7, $CHCl_3$) [lit:[13] $[\alpha]_D$ −10.6 (*c* 0.7, $CHCl_3$)]. The NMR spectra of (−)-dihydroepicrinine are same as those of (+)-dihydroepivittatine.

Asymmetric synthesis of 8-*O*-demethylmaritidines and siculines

(−)-8-*O*-Demethylmaritidine[15]

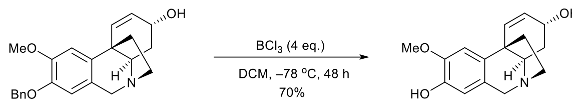

(−)-*cis*-**2c** (−)-8-*O*-demethylmaritidine

General procedure: Boron trichloride (0.2 mL, 1.0 M solution in DCM, 0.2 mmol) was added to a solution of (−)-*cis*-**2c** (36 mg, 0.1 mmol) in DCM (3 mL) at −78 °C. The mixture was stirred for 1d at −78 °C, and another 2 equivalents of boron tricholride were added. The reaction mixture was stirred for another day at −78 °C and saturated $NaHCO_3$ (5 mL) was added to quench the reaction. The mixture was extracted with $CHCl_3$ (3 × 5 mL). The organic layer was dried over anhydrous Na_2SO_4 and concentrated in vacuo. The residue was purified by chromatography on silica gel column eluting with DCM/MeOH (8:1) to afford (−)-8-*O*-demethylmaritidine as a white solid. 19.1 mg, 70% yield, R_f = 0.2 (DCM/MeOH = 5:1), mp 114–115 °C, $[\alpha]_D^{20}$ −30.4 (*c* 0.24, $CHCl_3$) [natural (−)-8-*O*-demethylmaritidine:[15] $[\alpha]_D^{20}$ −34.0 (*c* 0.24, $CHCl_3$)]. ^{1}H NMR (400 MHz, $CDCl_3$) δ 6.79 (s, 1H), 6.58 (d, *J* = 10.0 Hz, 1H), 6.49 (s, 1H), 5.97 (dd, *J* = 10.0, 5.0 Hz, 1H), 4.36 (d, *J* = 16.8 Hz, 1H), 4.38–4.32 (m, 1H), 3.85 (s, 3H), 3.76 (d, *J* = 16.8 Hz, 1H), 3.53–3.41 (m, 2H), 2.96–2.89 (m, 1H), 2.22–2.16 (m, 1H), 2.11–2.08 (m, 1H). 1.99–1.91 (m, 1H), 1.73–1.66 (m, 1H). ^{13}C NMR (100 MHz, $CDCl_3$) δ 145.9, 144.9, 135.6, 131.2, 128.1, 124.2, 113.6, 105.4, 63.5, 63.3, 61.2, 56.1, 53.2, 44.3, 43.6, 32.3.

(+)-8-*O*-Demethylmaritidine[16]

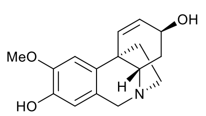

Using (+)-*cis*-**2c** (36 mg, 0.1 mmol), the (+)-8-O-demethylmaritidine was prepared (18.3 mg, 69% yield), R_f = 0.2 (DCM/MeOH = 5:1), white solid, mp 136–137 °C, $[\alpha]_D^{26}$ +25.0 (*c* 0.31, MeOH) [natural (+)-8-*O*-demethylmartidine:[16] $[\alpha]_D^{23}$ +22.9 (*c* 0.31, MeOH)]. The NMR spectra of (+)-8-*O*-demethylmaritidine are same as those of (−)-8-*O*-demethylmaritidine.

(+)-Siculine[17]

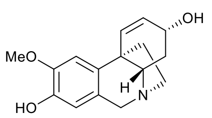

Using (+)-*trans*-**2c** (36 mg, 0.1 mmol), the (+)-siculine was prepared (20.0 mg, 73% yield), R_f = 0.2 (DCM/MeOH = 5:1), white solid, mp 213–215 °C, (+)-siculine·TFA $[\alpha]_D^{23}$ +48.2 (*c* 0.51, MeOH) [lit:[17] (+)-siculine·TFA, $[\alpha]_D^{23}$ +56.9 (*c* 0.51, MeOH)]. ^{1}H NMR (400 MHz, MeOD) δ 6.86 (s, 1H), 6.49 (s, 1H), 6.44 (d, *J* = 10.4 Hz, 1H), 5.74 (d, *J* = 10.4 Hz, 1H), 4.55 (d, *J* = 16.0 Hz, 1H), 4.33–4.29 (m, 1H), 4.07 (d, *J* = 16.0 Hz, 1H), 3.79–3.62 (m, 3H), 3.76 (s, 3H), 3.28–3.24 (m, 1H), 2.24–3.21 (m, 3H). 1.70–1.61 (m, 1H). ^{13}C NMR (100 MHz, MeOD): δ 148.6, 147.0, 134.9, 133.3, 127.4, 120.8, 114.8, 107.4, 68.3, 67.1, 60.4, 56.6, 53.5, 45.9, 43.7, 33.3.

(−)-Siculine[18]

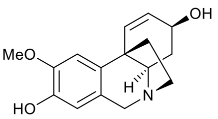

Using (−)-*trans*-**2c** (36 mg, 0.1 mmol), the (−)-siculine was prepared (19.5 mg, 72% yield), R_f = 0.2 (DCM/MeOH = 5:1), white solid, mp 208–209 °C, $[\alpha]_D^{23}$ −82.4 (*c* 1.3, $CHCl_3$) [natural (−)-siculine[18]: $[\alpha]_D$ −80.0 (*c* 1.3, $CHCl_3$)]. The NMR of (−)-siculine are same as those of (+)-siculine.

(E) Asymmetric Synthesis of *O*-Methyl Crinine-type Alkaloids and Analogues

(–)-Buphanisine

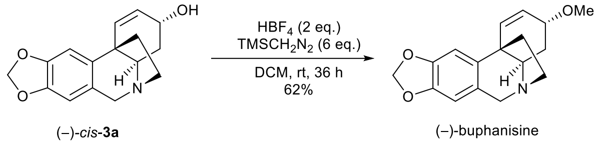

To a vigorously stirred mixture of (–)-*cis*-**3a** (30 mg, 0.11 mmol) and fluoroboric acid (50% w/v in Et_2O, 39 μL, 0.22 mmol) in DCM (4 mL) was added dropwise of $TMSCHN_2$ (2.0 M in hexane, 110 μL, 0.22mmol) at 0 °C. The yellow color of $TMSCHN_2$ disappeared immediately accompany with the evolution of nitrogen. The stirring was continued at 0°C, and a further portions of $TMSCHN_2$ (110 μL, 0.22mmol) was added dropwise. The mixture was continued stirred at 0 °C for 18 h, and a third portion of $TMSCHN_2$ (110 μL, 0.22 mmol) was added dropwise. After continued stirred at 0 ℃ for another 18 h, the reaction mixture was then poured into saturated $NaHCO_3$ (5 mL), extracted with DCM (5 mL × 3), washed with saturated brines, dried over amhydrous $MgSO_4$, and concentrated in vacuo to yield a brown oil. The brown oil was purified by chromatography on silica gel column (DCM/MeOH = 15:1) to afford (–)-buphanisine as a white solid. 19 mg, 62% yield, R_f = 0.4 (DCM/MeOH = 10:1), mp 123–125 °C, $[\alpha]_D^{23}$ –35.4 (*c* 1.1 $CHCl_3$) [lit.:[19] $[\alpha]_D^{23}$ –31 (*c* 1.1 $CHCl_3$)]. ^1H NMR (400 MHz, $CDCl_3$) δ 6.86 (s, 1H), 6.57 (d, *J* = 10.0 Hz, 1H), 6.52 (s, 1H), 6.02 (dd, *J* = 10.0, 5.2 Hz, 1H), 5.93 (dd, *J* = 5.2, 1.2 Hz, 2H), 4.56 (d, *J* = 16.4 Hz, 1H), 3.93 (d, *J* = 16.4 Hz, 1H), 3.87–3.84 (m, 1H), 3.65–3.58 (m, 1H), 3.56–3.52 (m, 1H), 3.38 (s, 3H). 3.07–3.00 (m, 1H), 2.41–2.38 (m, 1H), 2.29–2.22 (m, 1H), 2.07–2.00 (m, 1H), 1.70–1.62 (m, 1H). ^{13}C NMR (100 MHz, $CDCl_3$): δ 146.2, 145.8, 138.5, 133.0, 126.3, 125.5, 107.0, 103.1, 100.9. 72.7, 63.3, 62.5, 56.6, 53.7, 44.5, 44.3, 28.9.

(–)-Buphanidrine

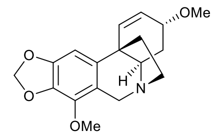

Using (–)-*cis*-**3d** (34 mg, 0.11 mmol) as the starting material, (–)-buphanidrine was prepared in the same way. 24 mg, 68% yield, R_f = 0.4 (DCM/MeOH = 10:1), white solid, mp 89–90°C, $[\alpha]_D^{23}$ –4.2 (*c* 1.0 $CHCl_3$). [lit.:[10] $[\alpha]_D^{23}$ –6.93 (*c* 1.0 $CHCl_3$)]. ^1H NMR (400 MHz, $CDCl_3$) δ 6.57 (s, 1H), 6.55 (d, *J* = 10.0 Hz, 1H), 5.97 (dd, *J* = 10.0, 1.6 Hz, 1H), 5.87–5.85 (m, 2H), 4.29 (d, *J* = 17.2 Hz, 1H), 3.97 (s, 3H), 3.87 (d, *J* = 17.2 Hz, 1H), 3.83–3.82 (m, 1H), 3.49–3.39 (m, 2H), 3.36 (s, 3H), 2.96–2.89 (m, 1H), 2.23–2.16 (m, 2H), 1.98–1.91 (m, 1H),1.65–1.57 (m, 1H). ^{13}C NMR (100 MHz, $CDCl_3$) δ 148.2, 140.9, 138.9, 133.5, 132.4, 125.5, 116.3, 100.6, 96.9. 72.3, 62.9, 59.2, 58.4, 56.5, 53.6, 44.4, 43.7, 28.3.

Compound 4

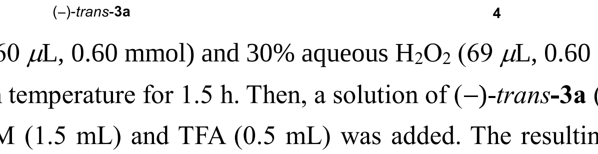

A solution of CCl_3CN (60 μL, 0.60 mmol) and 30% aqueous H_2O_2 (69 μL, 0.60 mmol) in dry DCM (1.5 mL) was stirred at room temperature for 1.5 h. Then, a solution of (–)-*trans*-**3a** (40 mg, 0.15 mmol) in a mixture solvent of DCM (1.5 mL) and TFA (0.5 mL) was added. The resulting reaction mixture was

stirred at room temperature for 48 h. Then, the reaction mixture was treated with 33% aqueous NH_3 solution and adjusted the pH to approximately10. The mixture was extracted with $CHCl_3$ (10 mL × 3). The $CHCl_3$ solvent was washed with saturated brines, dried over anhydrous Na_2SO_4, and concentrated in vacuo to yield a brown oil. The brown oil was purified by chromatography on silica gel column (DCM/MeOH = 15:1) to afford epoxide **4** as a semi oil 29 mg, 68% yield, R_f = 0.3 (DCM/MeOH = 10:1), $[\alpha]_D^{23}$ −35.4 (*c* 1.1 $CHCl_3$), ^{1}H NMR (400 MHz, $CDCl_3$) δ 6.91 (s, 1H), 6.51 (s, 1H), 5.93 (d, *J* = 1.6 Hz, 2H), 4.43 (d, *J* = 17.6 Hz, 1H), 4.18–4.14 (m, 1H), 3.86 (d, 3.6 Hz, 1H), 3.77 (d, *J* = 17.6 Hz, 1H), 3.49–3.48 (m, 1H), 3.39–3.32 (m, 1H). 2.96–2.86 (m, 2H), 2.55–2.48 (m, 1H), 2.10–1.89 (m, 3H), 1.46–1.37 (m, 1H). ^{13}C NMR (100 MHz, $CDCl_3$) δ 147.1, 146.7, 136.3, 124.1, 107.4, 103.0, 101.4, 67.6, 67.5, 61.4, 58.5, 54.5, 52.5, 41.9, 38.2, 29.8.

(–)-Flexinine

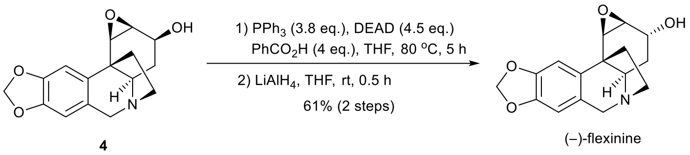

4 (–)-flexinine

To a stirred solution of epoxide **4** (29 mg, 0.10 mmol), triphenylphosphine (99 mg, 0.38 mmol) and benzoic acid (49 mg, 0.40 mmol) in dry THF (3 mL) was added dropwise diethyl azocarboxylate (78 mg, 0.45 mmol) at 0 °C (ice-water bath). After the addition the resulting reaction mixture was stirred at 80 °C for 5 h. The solvent was then removed in vacuo, and the residue was purified by chromatography on silica gel column (DCM/MeOH = 30:1) to afford the benzoate as a white foam. Then, the white foam was redissolved in dry THF (3 mL), and to the resulting solution was added $LiAlH_4$ (1.0 M in THF, 210 μL, 0.21 mmol) dropwise at room temperature. After continued stirring at room temperature for 30 min, the reaction mixture was quenched with water, extracted with $CHCl_3$ (5 mL × 3), washed with saturated brines, dried over anhydrous Na_2SO_4, and concentrated in vacuo to yield a colorless oil. The oil was purified by chromatography on silica gel column (DCM/MeOH = 15:1) to afford (–)-flexinine as a white solid. 18 mg, 61% yield, R_f = 0.3 (DCM/MeOH = 10:1), mp 229 °C. $[\alpha]_D^{25}$ −9.8 (*c* 0.11 MeOH) [natural (–)-flexinine:[20] $[\alpha]_D^{25}$ −12.7 (*c* 0.11 MeOH)]. ^{1}H NMR (400 MHz, $CDCl_3$) δ 6.92 (s, 1H), 6.48 (s, 1H), 5.93 (s, 2H), 5.57 (brs, 1H), 4.50–4.49 (m, 1H), 4.41 (d, *J* = 16.4 Hz, 1H), 3.79–3.75 (m, 2H), 3.38 (d, *J* = 11.2 Hz, 1H), 3.31–3.28 (m, 1H), 3.26–3.24 (m, 1H), 2.91–2.84 (m, 1H), 2.49–2.41 (m, 1H), 2.09–2.03 (m, 1H), 1.76–1.73 (m, 1H), 1.60–1.53 (m, 1H). ^{13}C NMR (100 MHz, $CDCl_3$) δ 146.7, 146.2, 136.7, 124.4, 107.2, 102.8, 101.1, 64.6, 61.7, 61.4, 56.3, 53.2, 52.0, 42.0, 38.5, 29.0.

(–)-Augustine

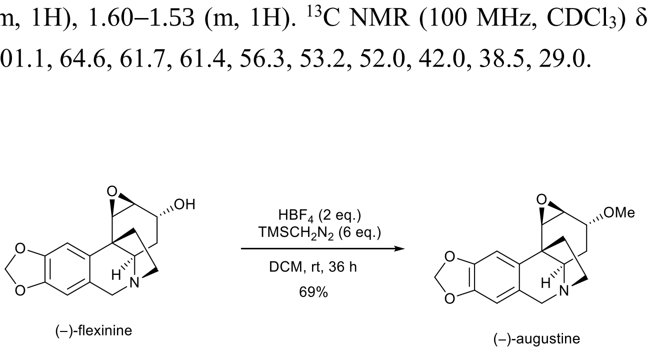

(–)-flexinine (–)-augustine

According to the procedure for the synthesis of (–)-buphanisine, (–)-augustine was synthesized from ((–)-flexinine,18 mg, 0.06 mmol) as a white solid in 69% yield (13 mg). R_f = 0.5 (DCM/MeOH = 10:1), mp 171–173 °C, $[\alpha]_D^{24}$ −40.2 (*c* 0.105 MeOH) [natural (–)-augustine:[20] $[\alpha]_D^{24}$ −44.8 (*c* 0.105 MeOH)], ^{1}H NMR (400 MHz, $CDCl_3$) δ 6.91 (s, 1H), 6.48 (s, 1H), 5.90 (d, *J* = 2 Hz, 2H), 4.39 (d, *J* = 16.8 Hz, 1H), 3.99–3.97 (m, 1H), 3.78 (d, *J* = 3.2 Hz, 1H), 3.72 (d, *J* = 16.8 Hz, 1H), 3.43 (s, 3H),

3.34–3.32 (m, 1H), 3.25–3.18 (m, 1H), 3.15–3.11 (m, 1H), 2.86–2.79 (m, 1H), 2.44–2.37 (m, 1H), 2.05–1.99 (m, 1H). 1.79–1.76 (m, 1H), 1.44–1.37 (m, 1H). ^{13}C NMR (100 MHz, $CDCl_3$) δ 146.3, 145.9, 137.8, 126.6, 107.2, 102.7, 101.0, 75.0, 62.4, 61.8, 57.8, 55.2, 53.9, 52.5, 41.7, 39.3, 25.4.

(F) The Comparison of Our Results with Those Reported in Literatures

entry	Crinine-type alkaloids	Chiral starting materials or catalysts used in lit.	Overall yields in lit. (steps)	Our result
1	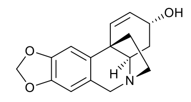 (–)-crinine	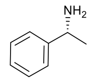	6.4% (10 steps) [21]	24.3% (5 steps)
2	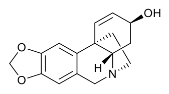 (+)-vittatine	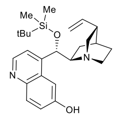	9.5 % (15 steps)[22]	25.0% (5 steps)
		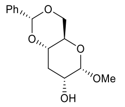	3.3% (15 steps)[8]	
		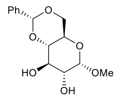	2.9% (20 steps)[23]	
3	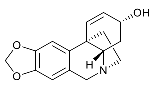 (+)-epivittatine	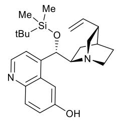	13.2% (15 steps)[22]	29.4% (5 steps)
4	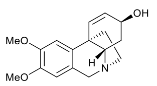 (+)-maritidine	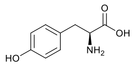	0.6% (9 steps)[24]	21.2% (5 steps)
5	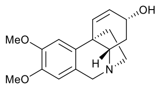 (+)-epimaritidine	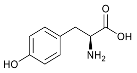	3.7% (8 steps)[24]	25.4% (5 steps)
6	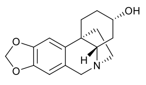 (+)-dihydroepivittatine	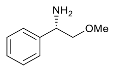	4.1% (17 steps)[14]	26.2% (6 steps)
7	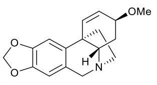 (+)-buphanisine	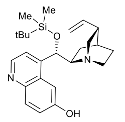	11.9% (16 steps)[20]	15.1% (6 steps)

Reference：

1 S. Kodama, H. Takita, T. Kajimoto, K. Nishide and M. Node, *Tetrahedron*, 2004, **60**, 4901.

2 G. Pandey, N. R. Gupta and Gadre, S. R, *Eur. J. Org. Chem.* 2011, 740.

3 S. Henry, R. Kidner, M. R. Reisenauer, I. V. Magedov, R. Kiss, V. Mathieu, F. Lefranc, R. Dasari, A. Evidente, X. Yu, X. Ma, A. Pertsmlidis, R. Cencic, J. Pelletier, D. A. Cavazos, A. J. Brenner, A. V. Aksenov, S. Rogelj, A. Kornienko, and L. V. Frolova, *Eur. J. Med. Chem.* 2016, **120**, 313.

4 H. F. Anwar and T. V. Hansen, *Synlett*, 2008, 2681.

5 F. Viladomat, C. Codina, J. Bastida, and S. Mathee, Campbell, W. E. *Phytochemistry* 1995, **40**, 961.
6 J. Renz, D. Stauffacher, and E. Seebeck, *Helv. Chim. Acta.* 1955, **38**, 1209.
7 F. Viladomat, J. Bastida, C. Codina, W. E. Campbell and S. Mathee, *Phytochemistry*, 1995, **40**, 307.
8 M. Bohno, H. Imase and N. Chida, *Chem. Commun.*, 2004, 1086.
9 H.-G. Boit, and H. Ehmke, *Chem. Ber.* 1957, **90**, 369.
10 W. C. Wildman, *J. Am. Chem. Soc.* 1958, **80**, 2567.
11 S. Ghosal, S. Ashutosh & Razdan, *Phytochemistry* 1985, **24**, 635.
12 O. Hoshino, S. Sawaki, N. Shimamura, and A. Onodera, *Chem. Pharm. Bull.* 1987, **35**, 2734.
13 H. Irie, S. Uyeo and A. Yoshitake, *J. Chem. Soc. C*, 1968, 1802.
14 G. Rousseau, R. Lebeuf, K. Schenk, F. Castet, F. Robert and Y. Landais, *Chem. Eur. J.*, 2014, **20**, 14771.
15 J. J. Nair, A. K. Machocho, W. E. Campbell, R. Brun, F. Viladomat, C. Codina, and J. Bastida, *Phytochemistry* 2000, **54**, 945.
16 M. Kihara, T. Koike, Y. Imakura, K. Kida, T. Shingu and S. Kobayashi, *Chem. Pharm. Bull.*, 1987, **35**, 1070.
17 C.-K. Chen, F.-H. Lin, L.-H. Tseng, C.-L. Jiang and S.-S. Lee, *J. Nat. Prod.*, 2011, **74**, 411.
18 V. Pabuççuoğlu, P. Richomme, T. Gözler, B. Kivçak, A. J. Freyer and M. Shamma, *J. Nat. Prod.*, 1989, **52**, 785.
19 H. M. Fales and W. C. Wildman, *J. Am. Chem. Soc.*, 1958, **80**, 4395.
20 L. H. Pham, W. Döpke, J. Wagner and C. Mügge, *Phytochemistry,* 1998, **48**, 371.
21 L. E. Overman, and S. Sugai, *Helv. Chem. Acta.* 1985, **68**, 745.
22 M.-X. Wei, C.-T. Wang, J.-Y. Du, H. Qu, P.-R. Yin, X. Bao, X.-Y. Ma, X.-H. Zhao, G.-B. Zhang and C.-A. Fan, *Chem. Asian J.*, 2013, **8**, 1966.
23 M. Bohno, K. Sugie, H. Imase, Y. N. Yusof, T. Oishib and N. Chida, *Tetrahedron*, 2007, **63**, 6977.
24 T. Ohkuma, T. Hattori, H. Ooka, T. Inoue and R. Noyori, *Org. Lett.*, 2004, **6**, 2681.

(G) NMR Spectra of *rac*-1e and Synthesized Crinine-type Alkaliods and Analogues

***rac*-1e**

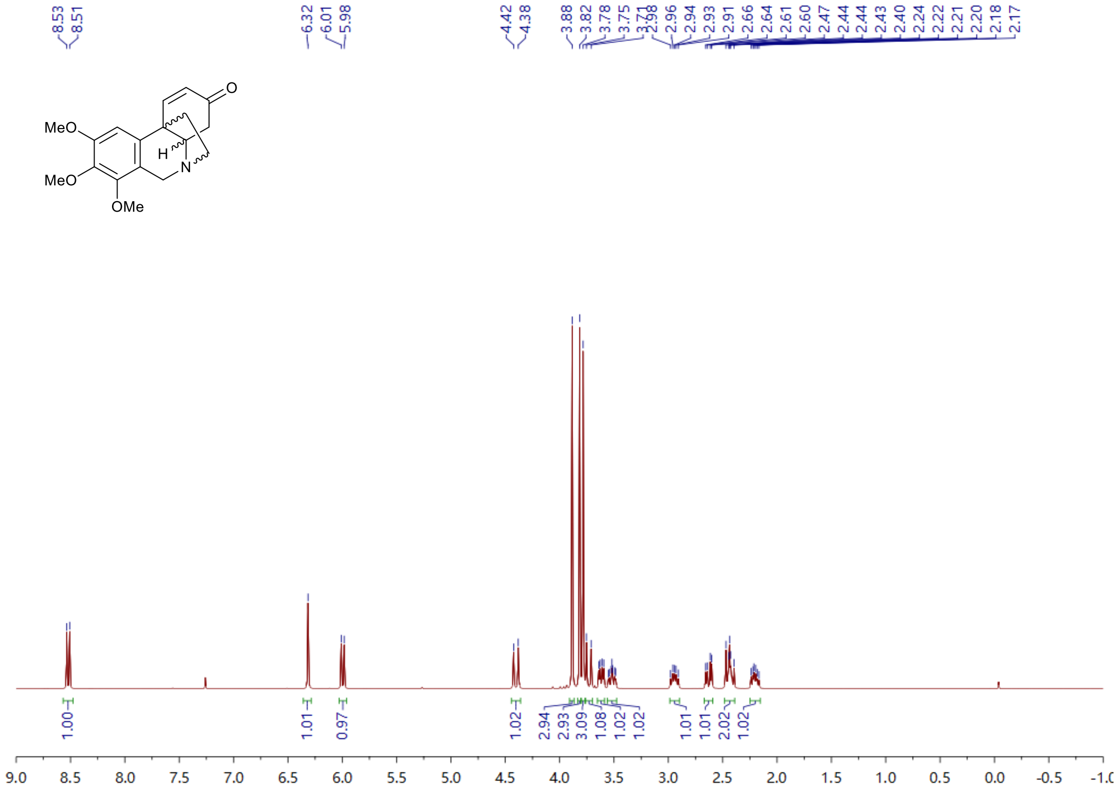

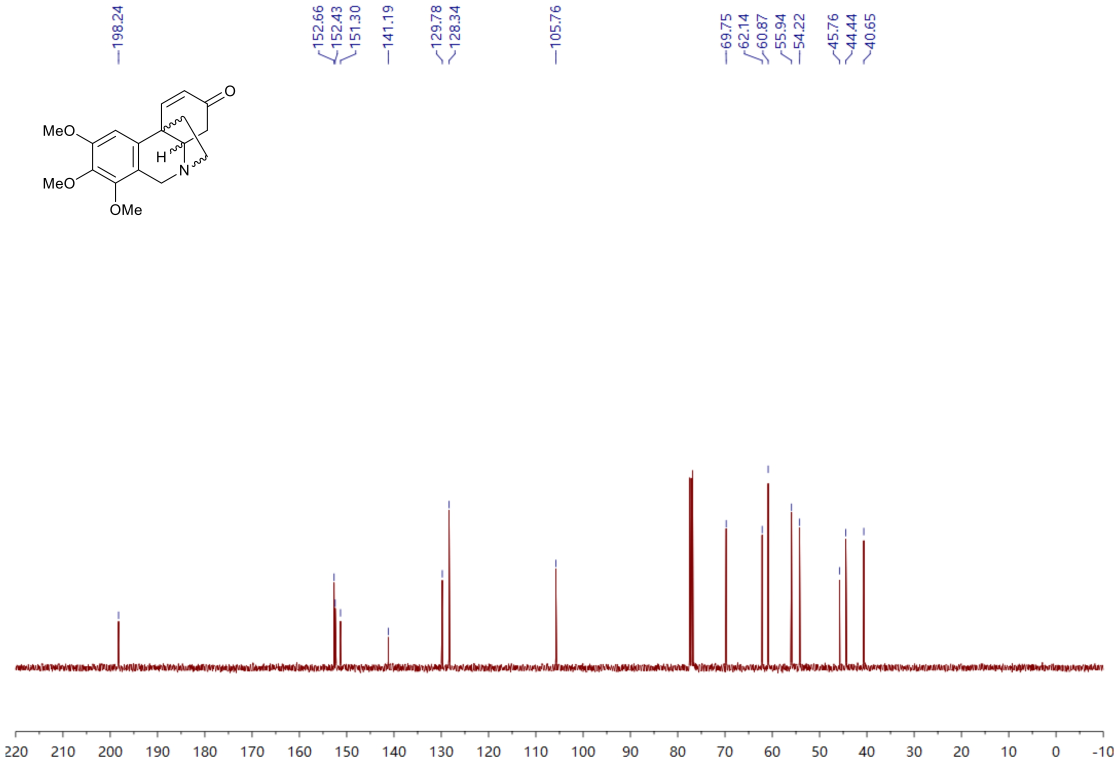

(–)-Crinine ((–)-*cis*-2a)

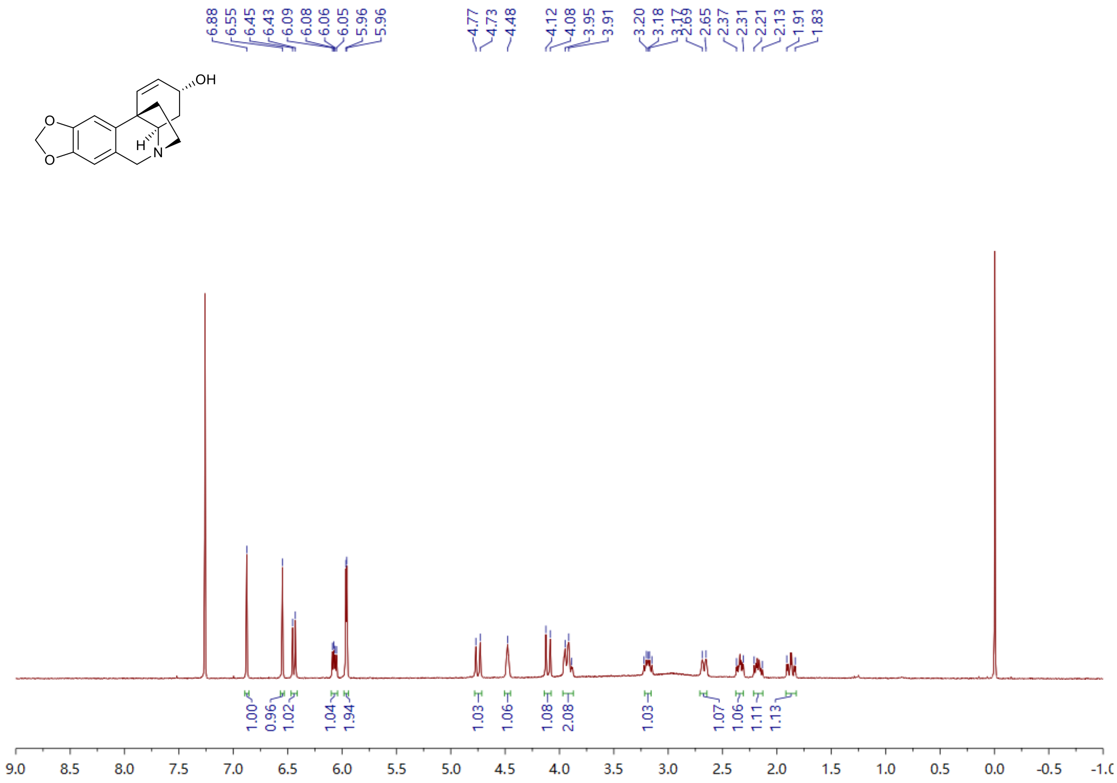

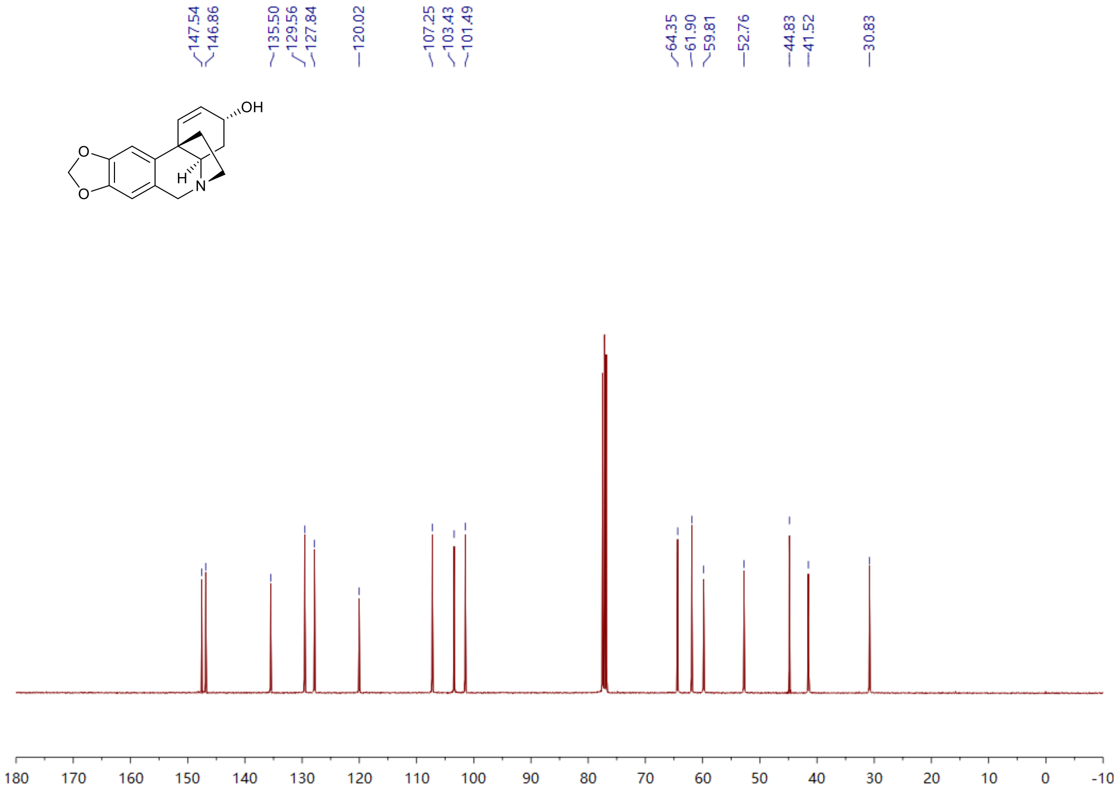

(+)-Epivittatine ((+)-*trans*-2a)

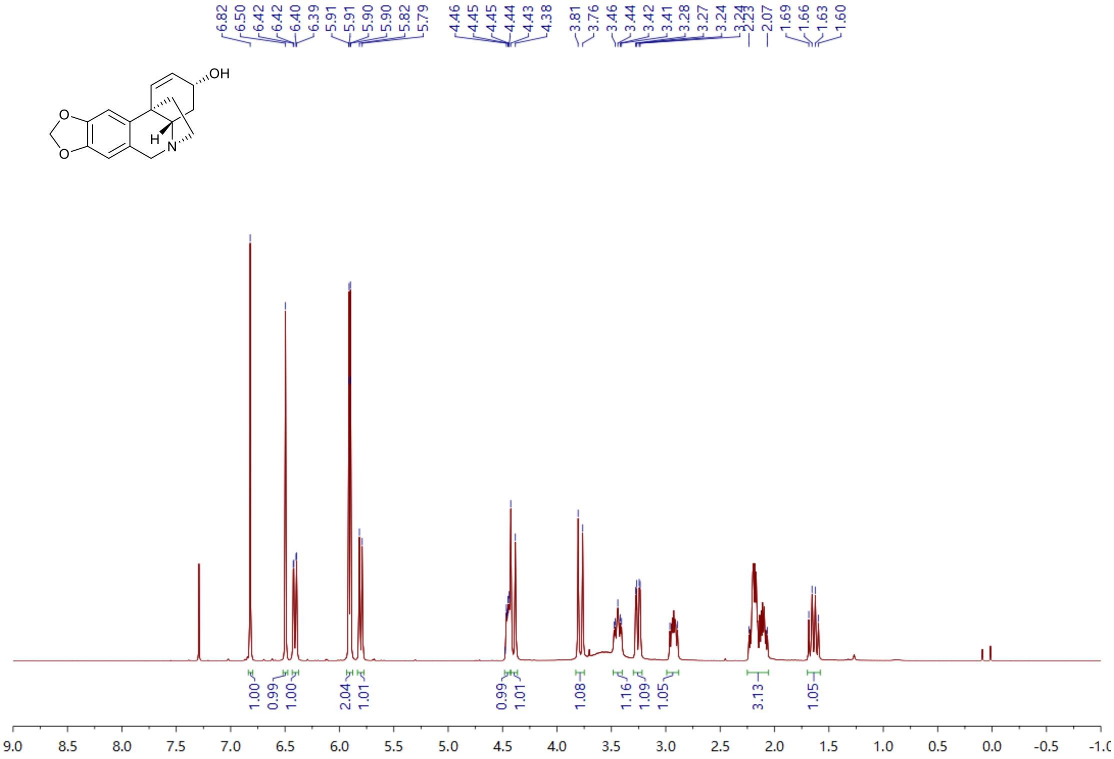

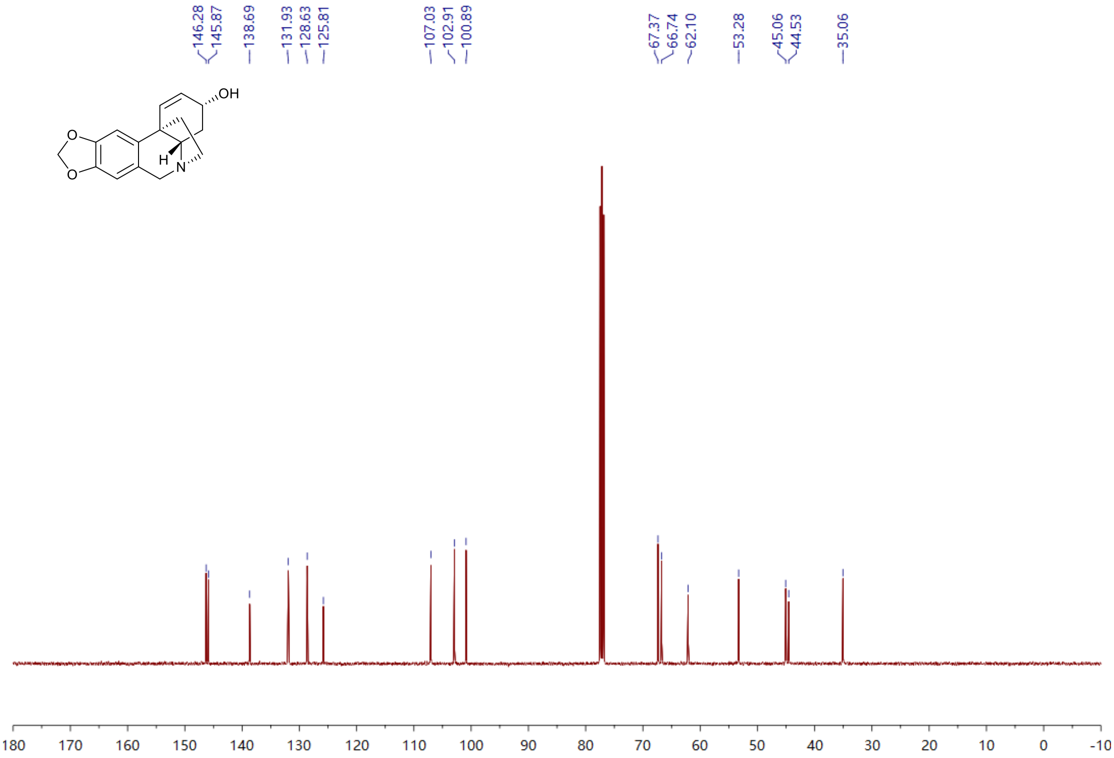

(–)-Maritidine ((–)-*cis*-2b)

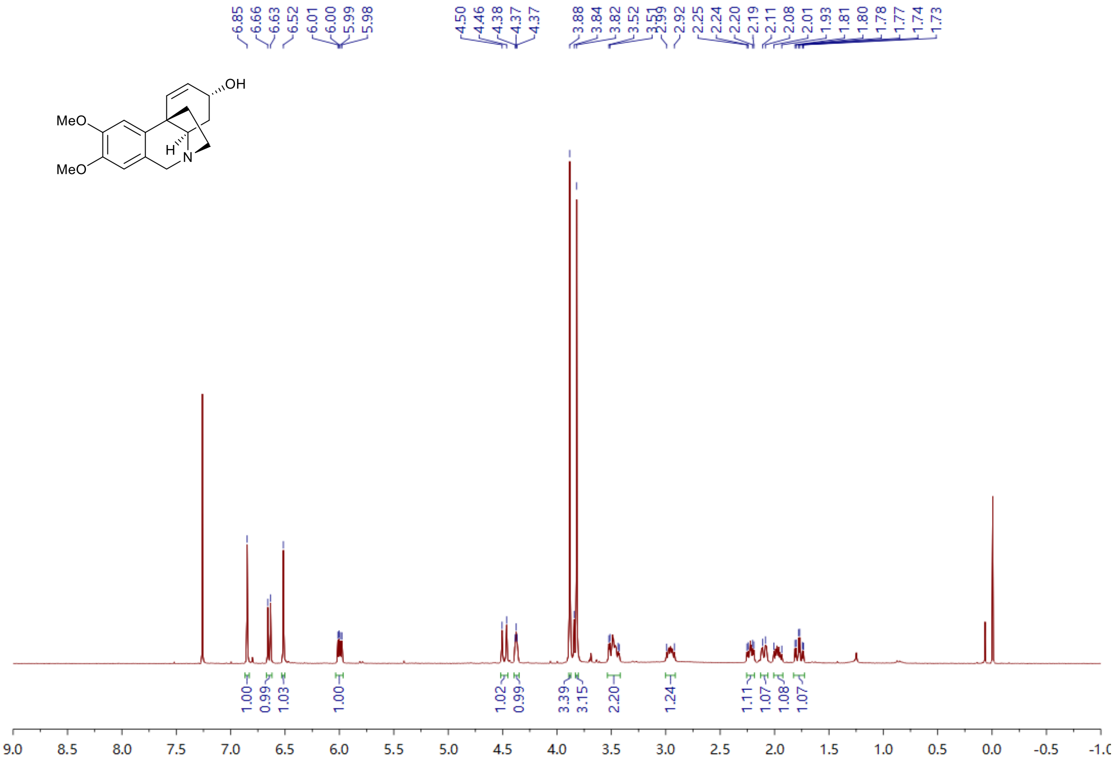

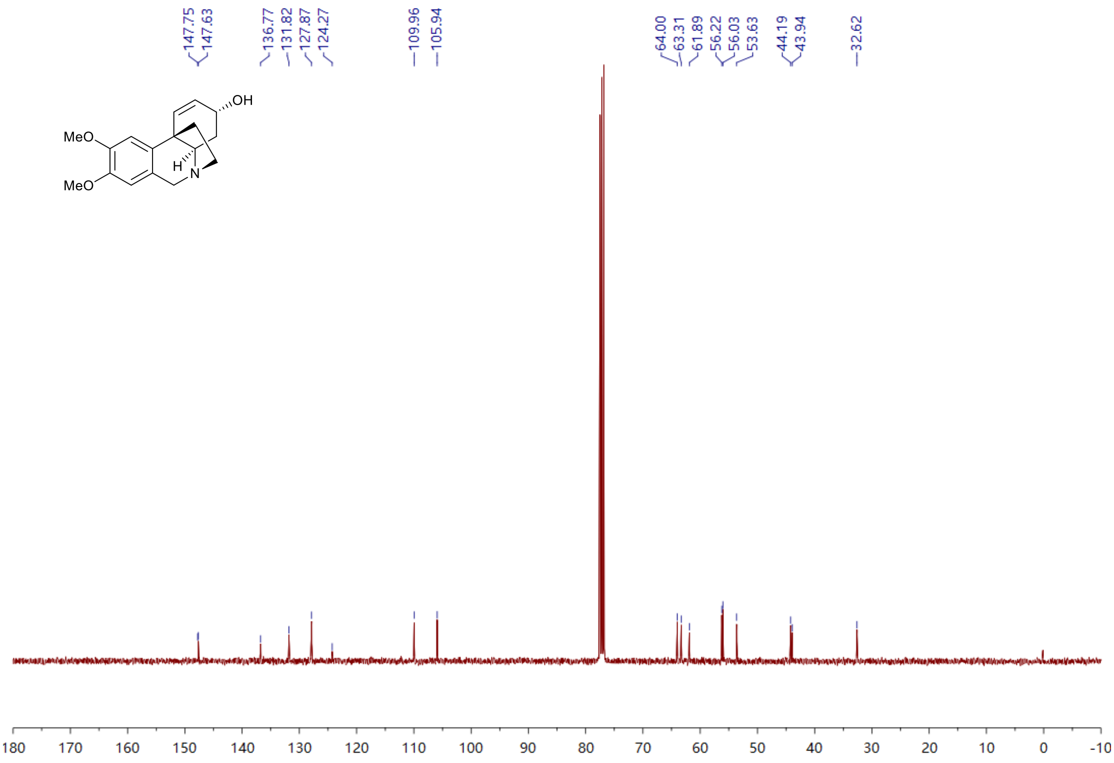

(+)-Epimaritidine ((+)-*trans*-2b)

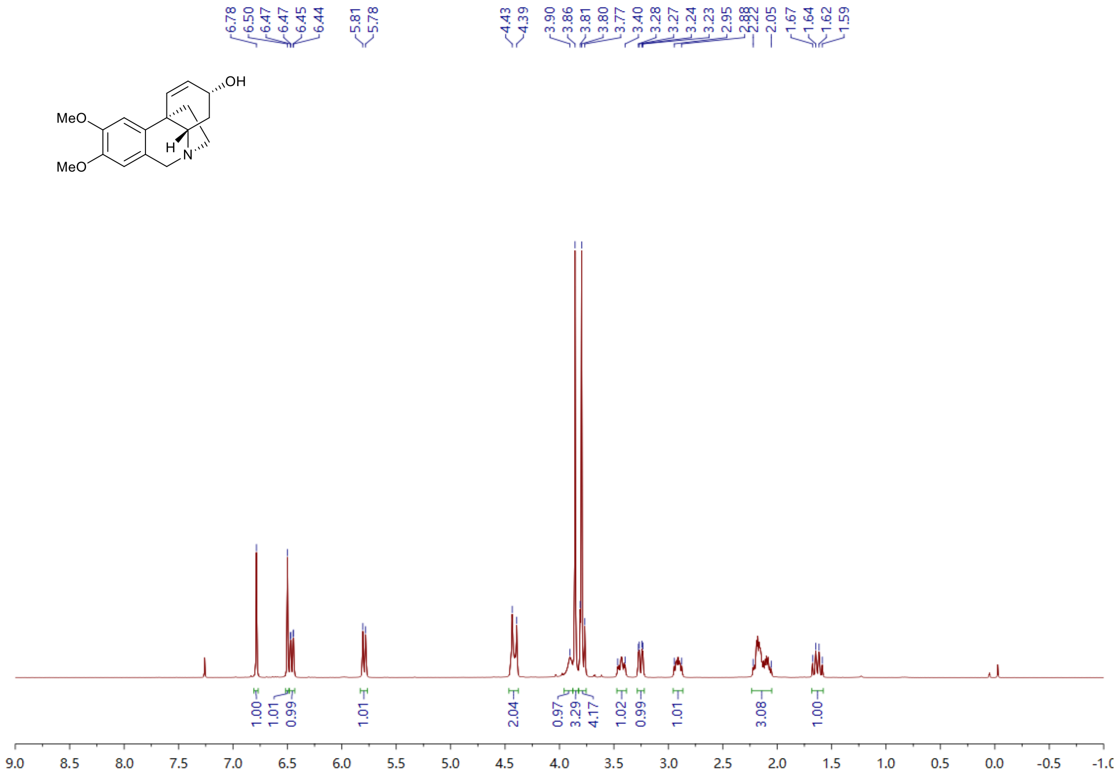

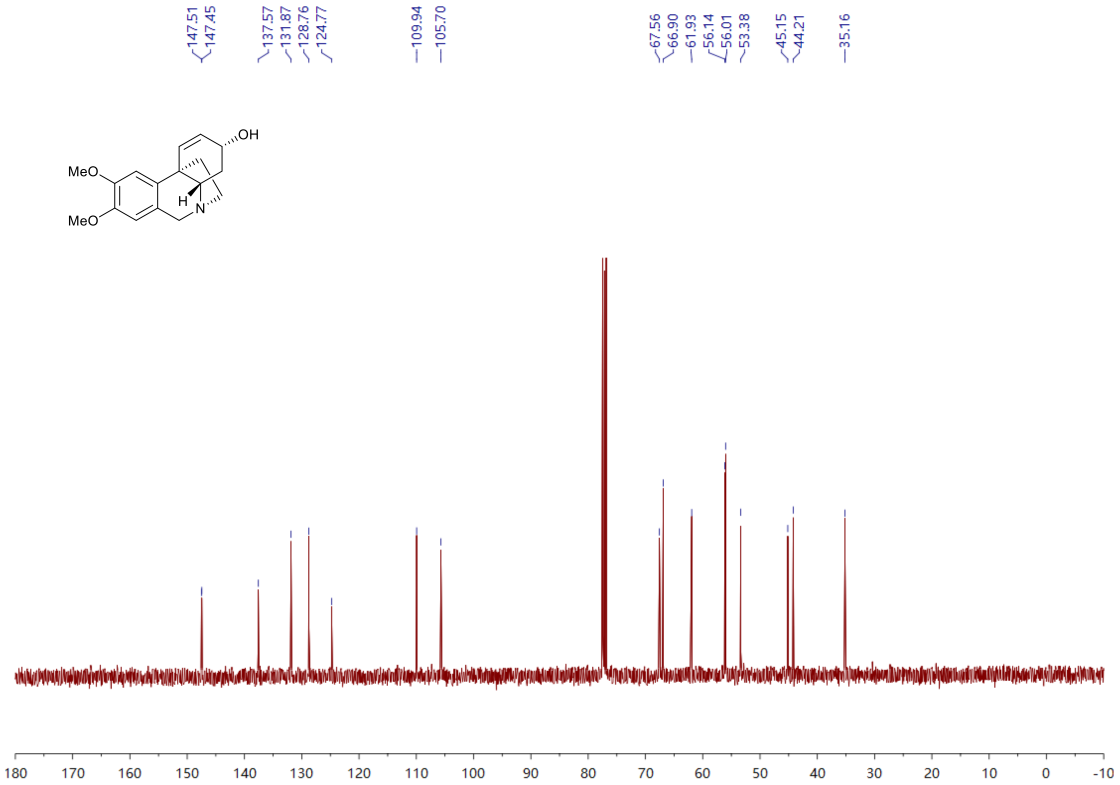

(−)-*cis*-2c

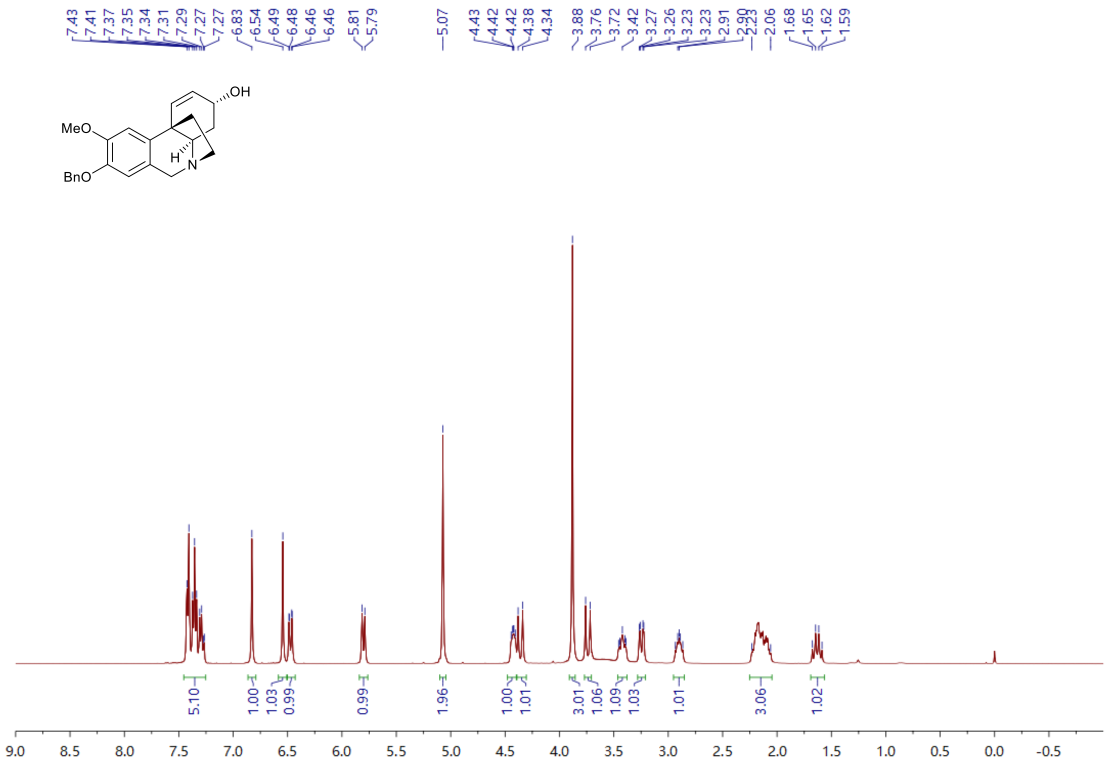

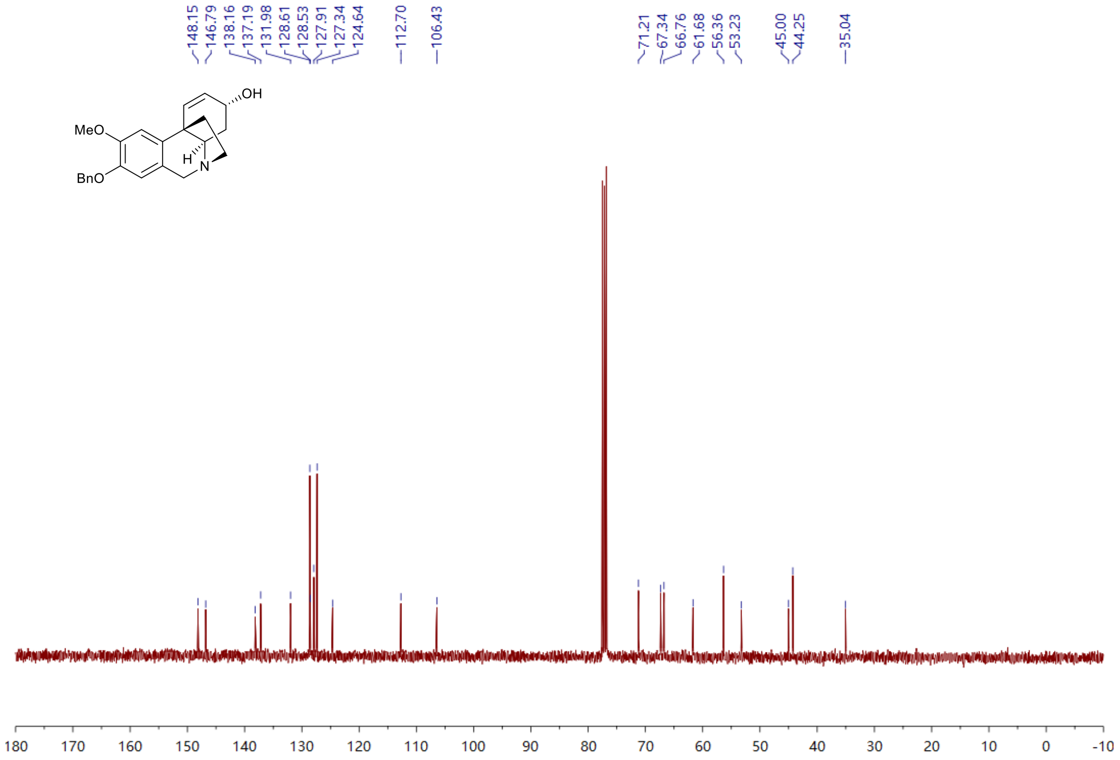

(+)-*trans*-2c

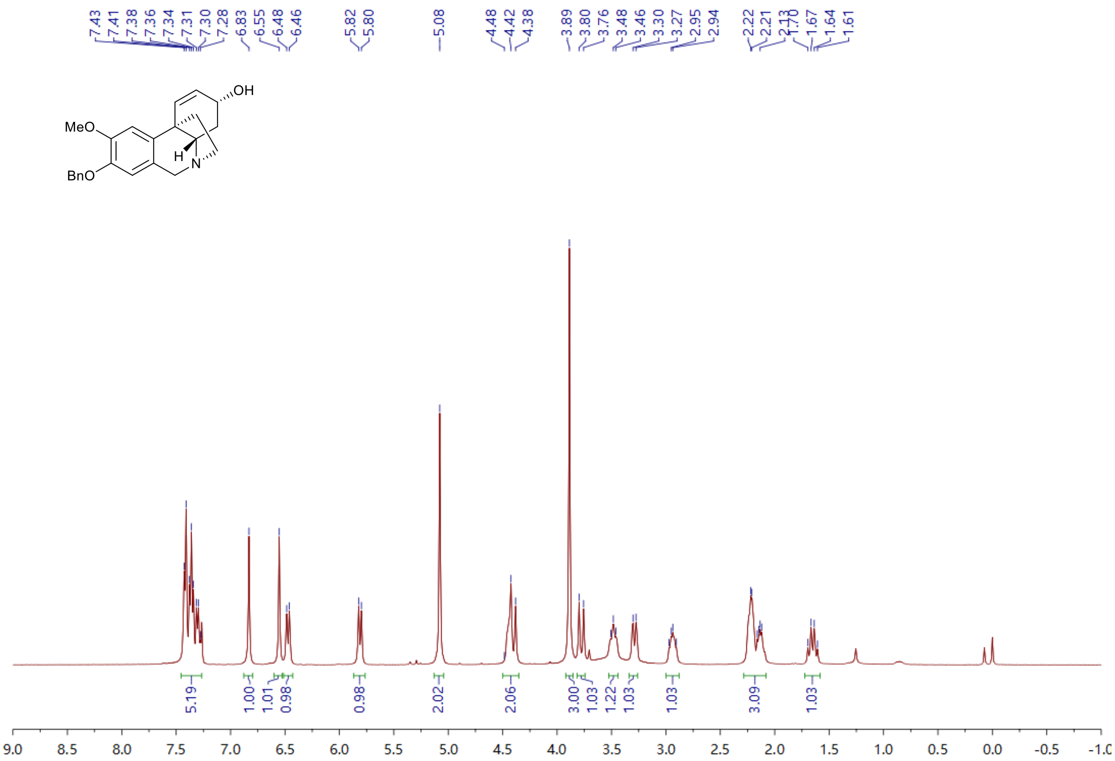
7.43
7.41
7.38
7.36
7.34
7.31
7.30
7.28
6.83
6.55
6.48
6.46
5.82
5.80
5.08
4.48
4.42
4.38
3.89
3.80
3.76
3.48
3.46
3.30
3.27
2.95
2.94
2.22
2.21
2.13
1.70
1.67
1.64
1.61
OH
MeO
BnO
H
N
5.19
1.00
1.01
0.98
0.98
2.02
2.06
3.00
1.03
1.22
1.03
1.03
3.09
1.03
9.0 8.5 8.0 7.5 7.0 6.5 6.0 5.5 5.0 4.5 4.0 3.5 3.0 2.5 2.0 1.5 1.0 0.5 0.0 -0.5 -1.0

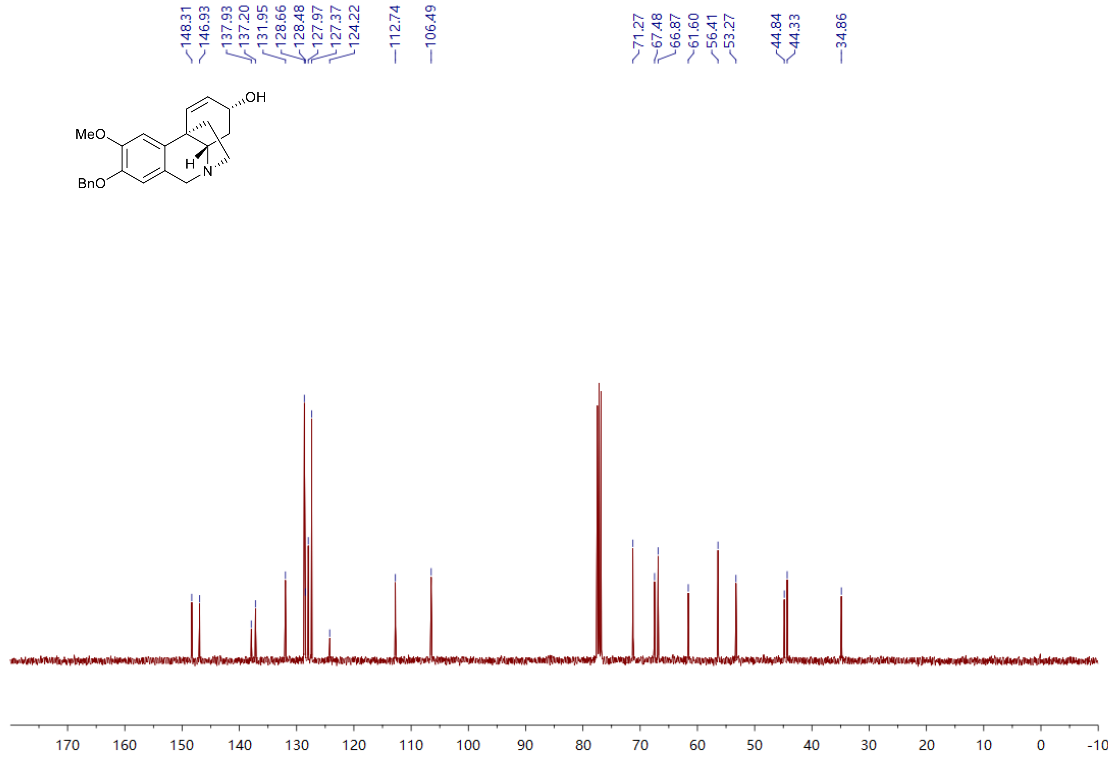
148.31
146.93
137.93
137.20
131.95
128.66
128.48
127.97
127.37
124.22
112.74
106.49
71.27
67.48
66.87
61.60
56.41
53.27
44.84
44.33
34.86
OH
MeO
BnO
H
N
170 160 150 140 130 120 110 100 90 80 70 60 50 40 30 20 10 0 -10

(–)-Powelline ((–)-*cis*-2d)

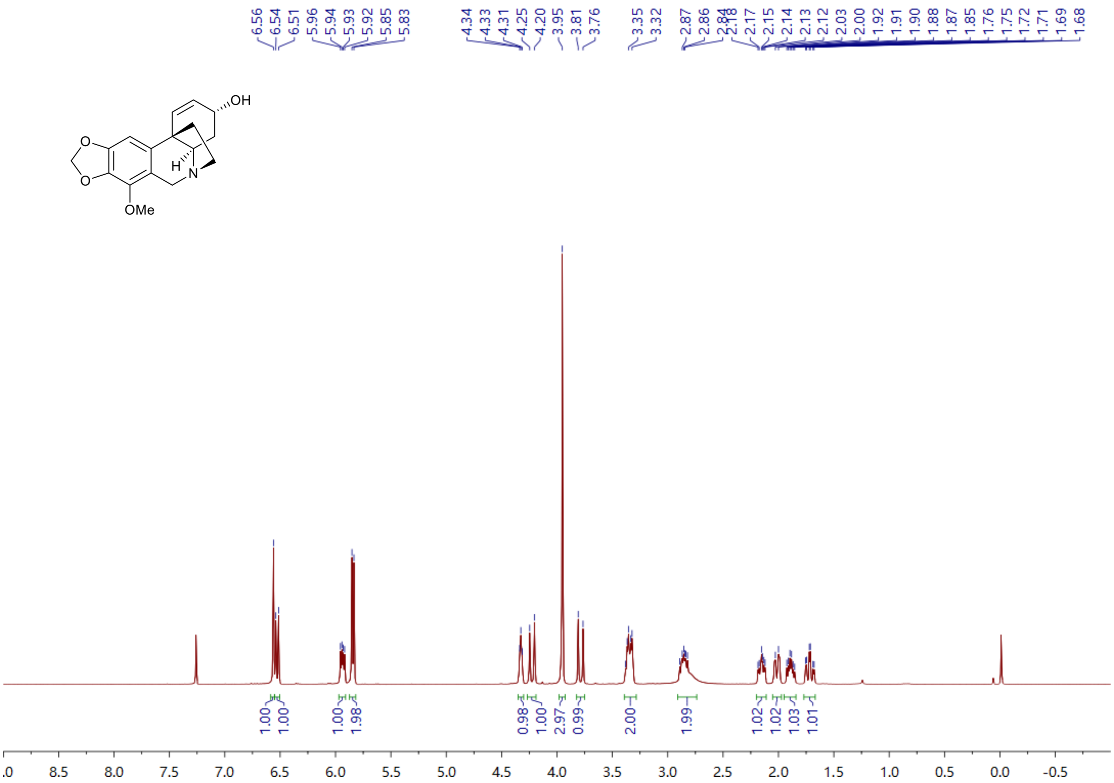

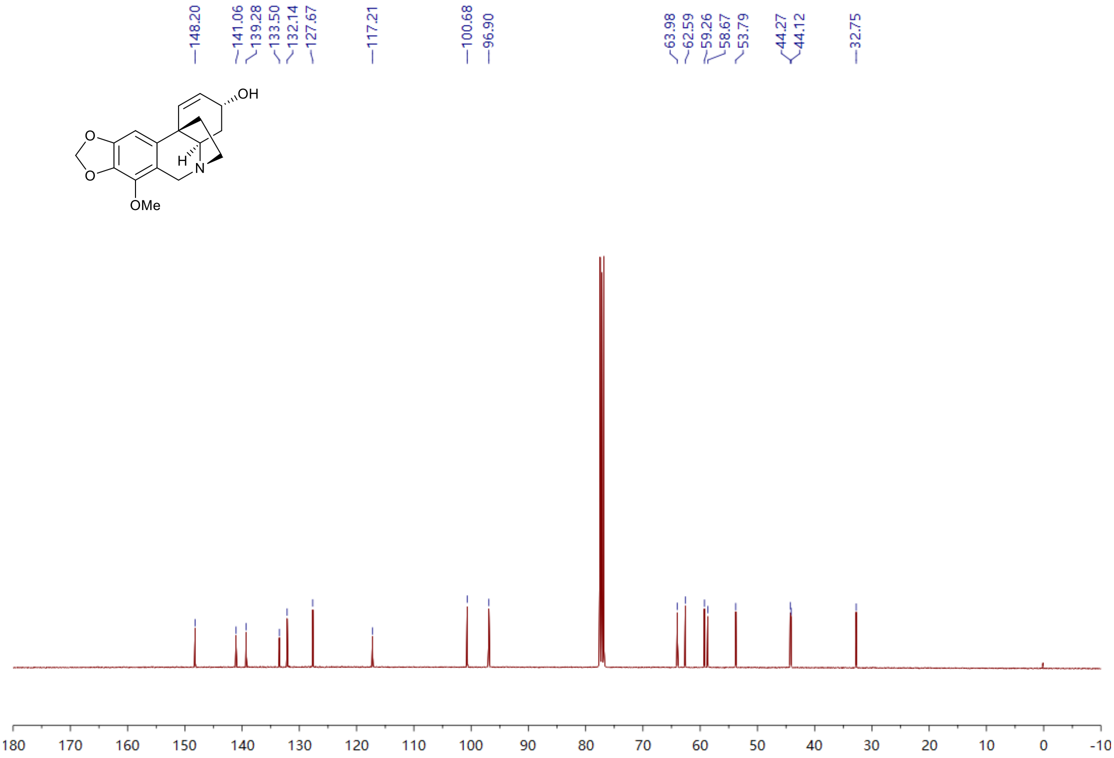

(+)-Epipowelline ((+)-*trans*-2d)

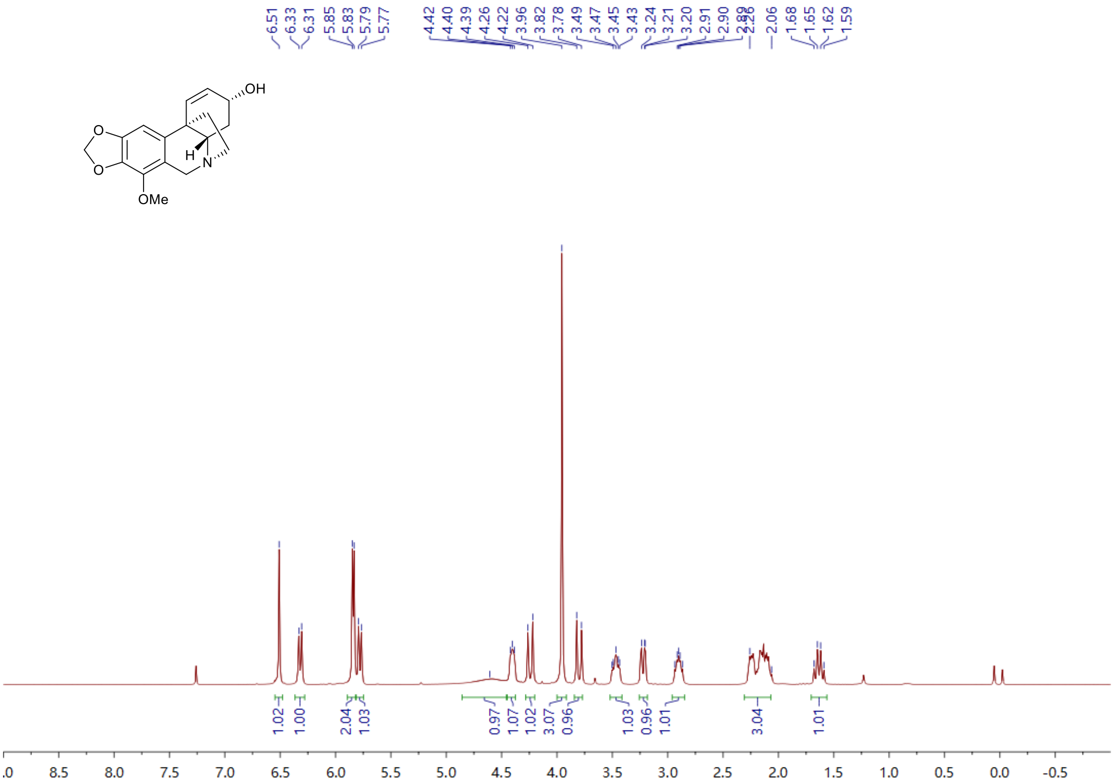

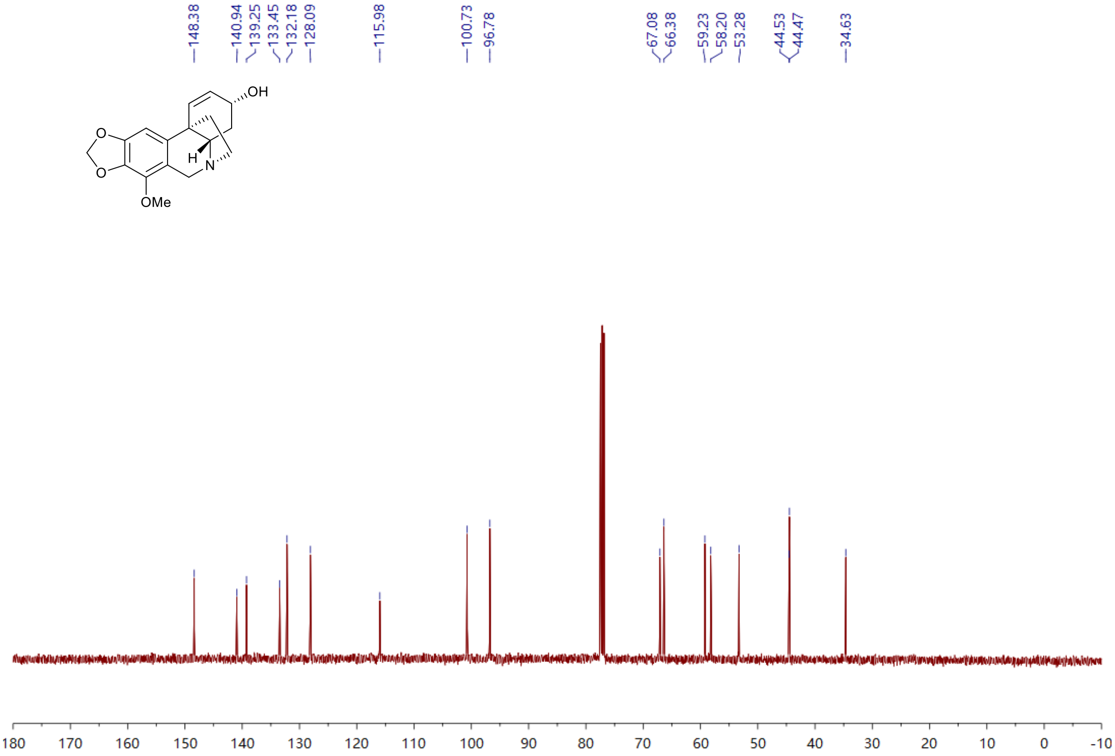

(−)-*cis*-2e

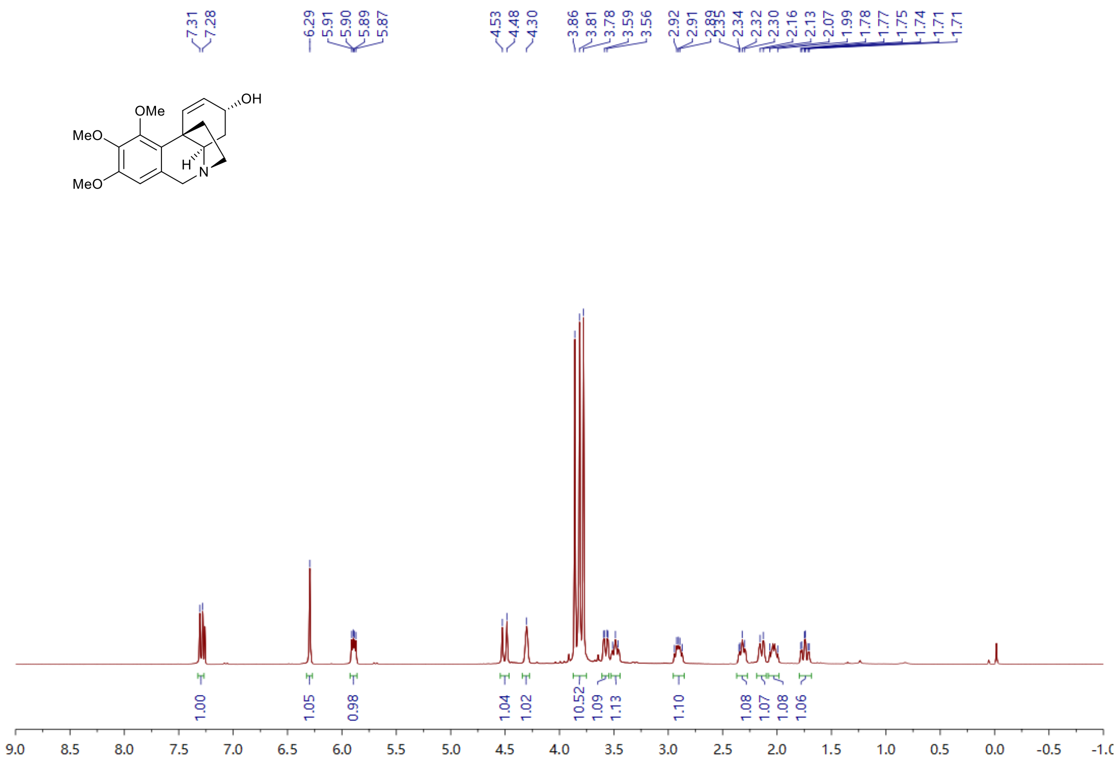

7.31
7.28
6.29
5.91
5.90
5.89
5.87
4.53
4.48
4.30
3.86
3.81
3.78
3.59
3.56
2.92
2.91
2.34
2.32
2.30
2.16
2.13
2.07
1.99
1.78
1.77
1.75
1.74
1.71
1.71
1.00
1.05
0.98
1.04
1.02
10.52
1.09
1.13
1.10
1.08
1.07
1.08
1.06
9.0 8.5 8.0 7.5 7.0 6.5 6.0 5.5 5.0 4.5 4.0 3.5 3.0 2.5 2.0 1.5 1.0 0.5 0.0 -0.5

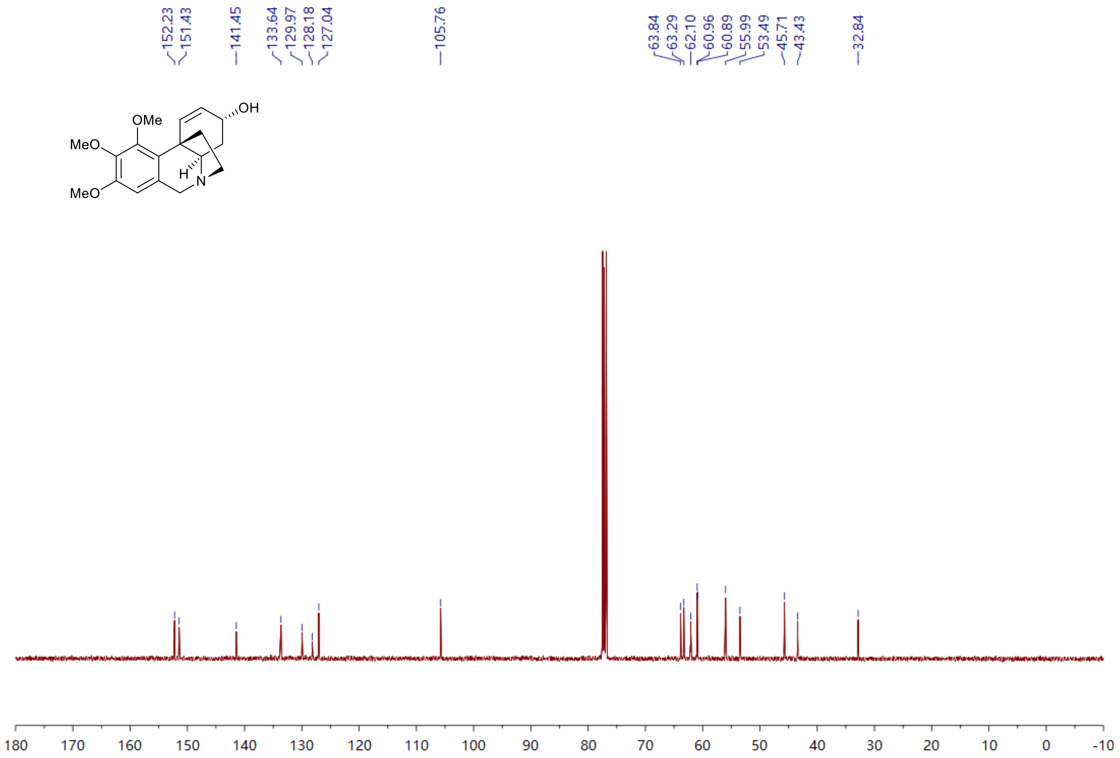

152.23
151.43
141.45
133.64
129.97
128.18
127.04
105.76
63.84
63.29
62.10
60.96
60.89
55.99
53.49
45.71
43.43
32.84
180 170 160 150 140 130 120 110 100 90 80 70 60 50 40 30 20 10 0 -10

(+)-*trans*-2e

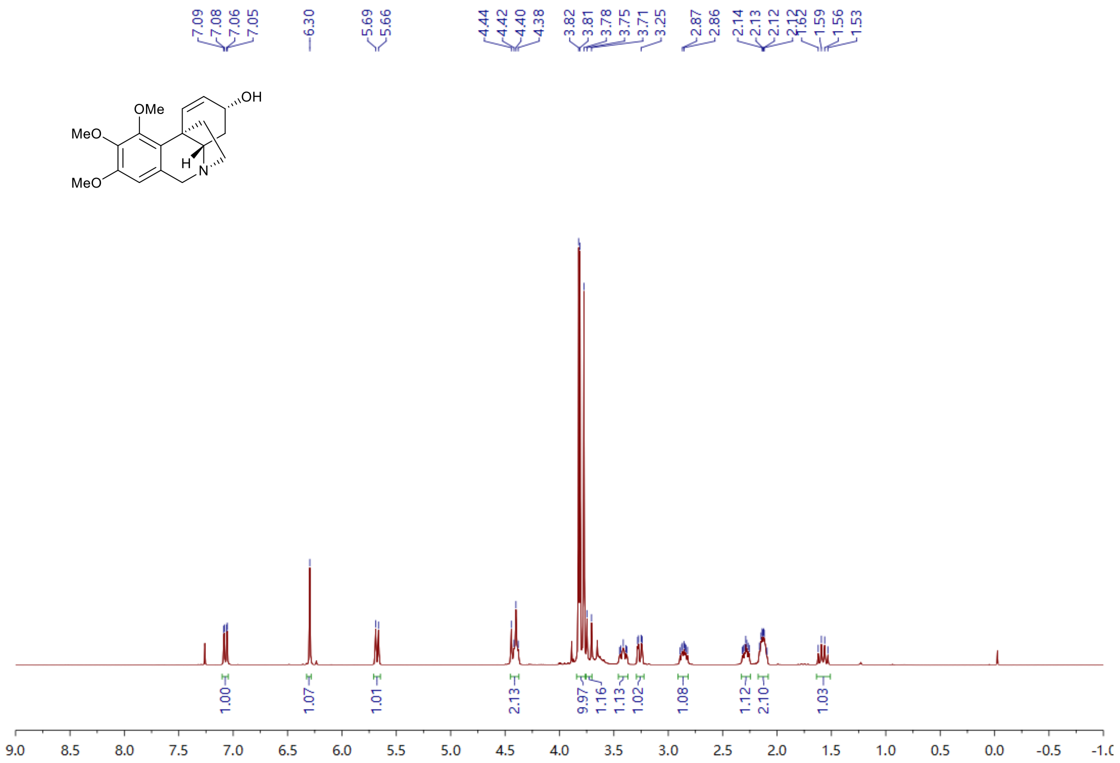
7.09
7.08
7.06
7.05
6.30
5.69
5.66
4.44
4.42
4.40
4.38
3.82
3.81
3.78
3.75
3.71
3.25
2.87
2.86
2.14
2.13
2.12
2.12
1.62
1.59
1.56
1.53
OMe
MeO
MeO
OH
H
N
1.00
1.07
1.01
2.13
9.97
1.16
1.13
1.02
1.08
1.12
2.10
1.03
9.0 8.5 8.0 7.5 7.0 6.5 6.0 5.5 5.0 4.5 4.0 3.5 3.0 2.5 2.0 1.5 1.0 0.5 0.0 -0.5

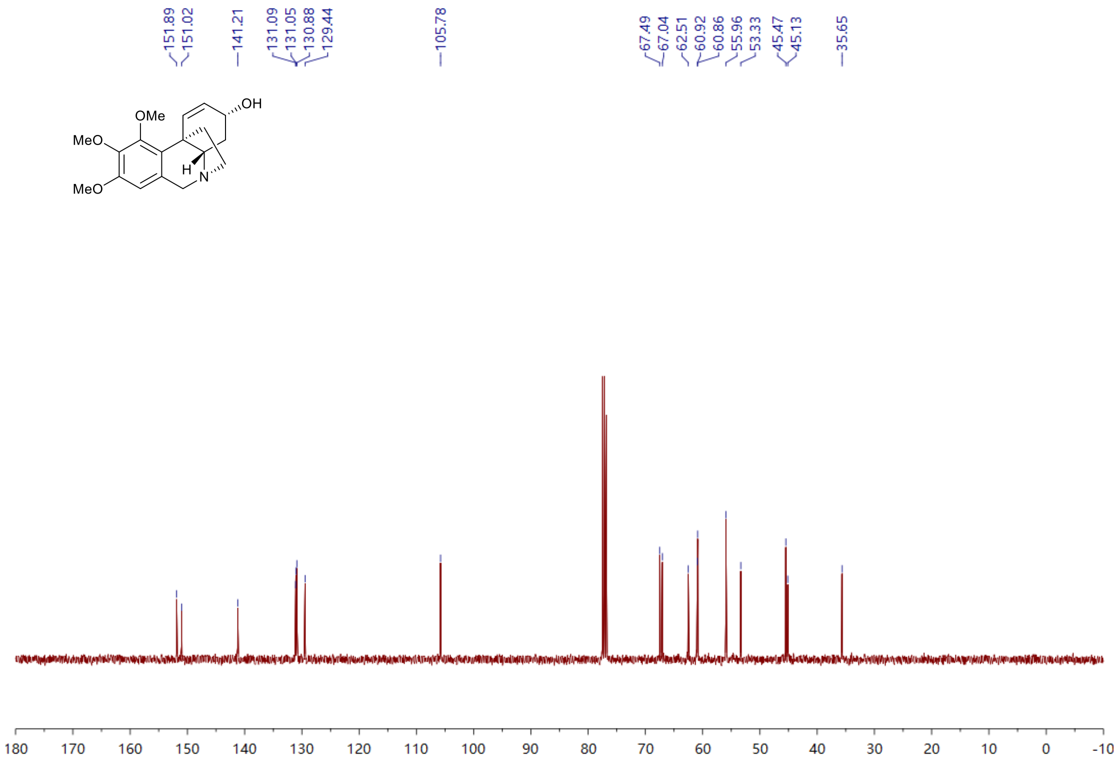
151.89
151.02
141.21
131.09
131.05
130.88
129.44
105.78
67.49
67.04
62.51
60.92
60.86
55.96
53.33
45.47
45.13
35.65
OMe
MeO
MeO
OH
H
N
180 170 160 150 140 130 120 110 100 90 80 70 60 50 40 30 20 10 0 -10

(–)-Dihydrocrinine

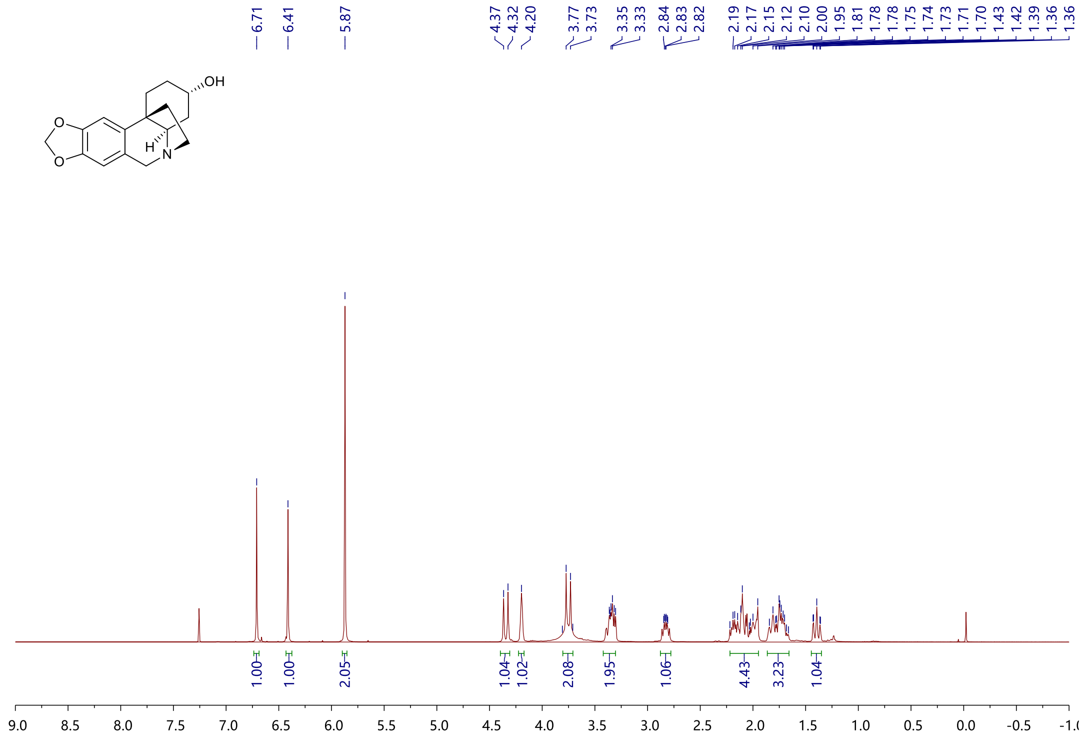

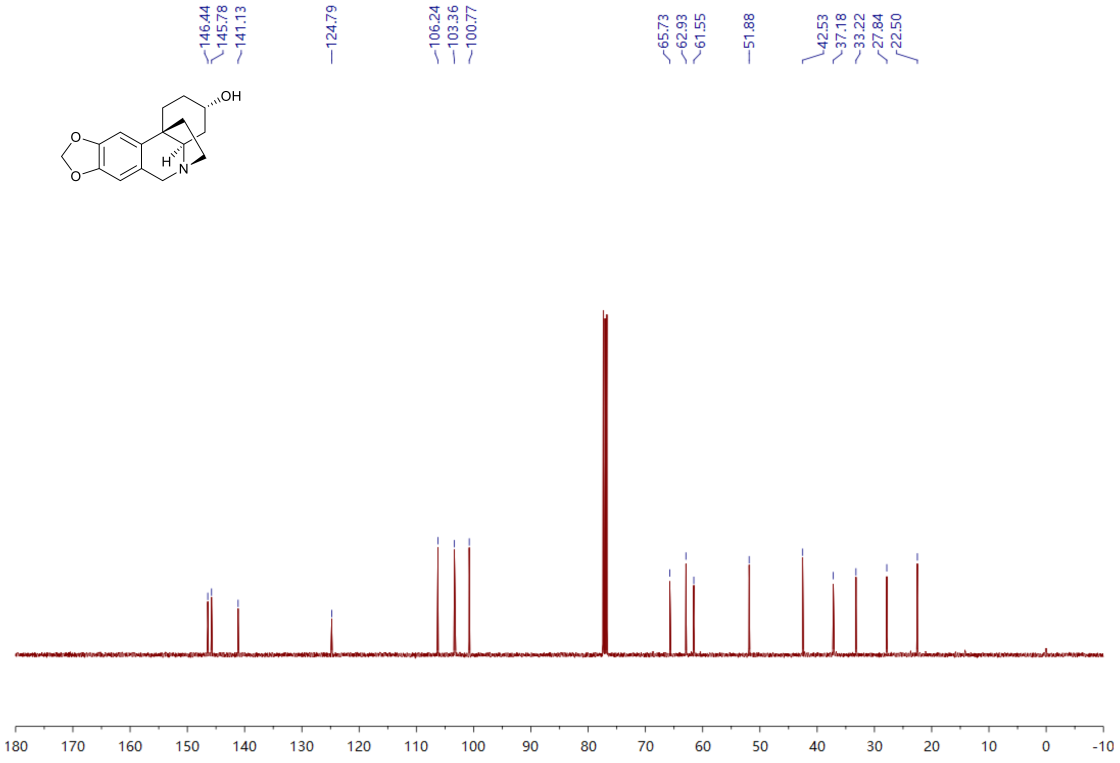

(+)-Dihydroepivittatine

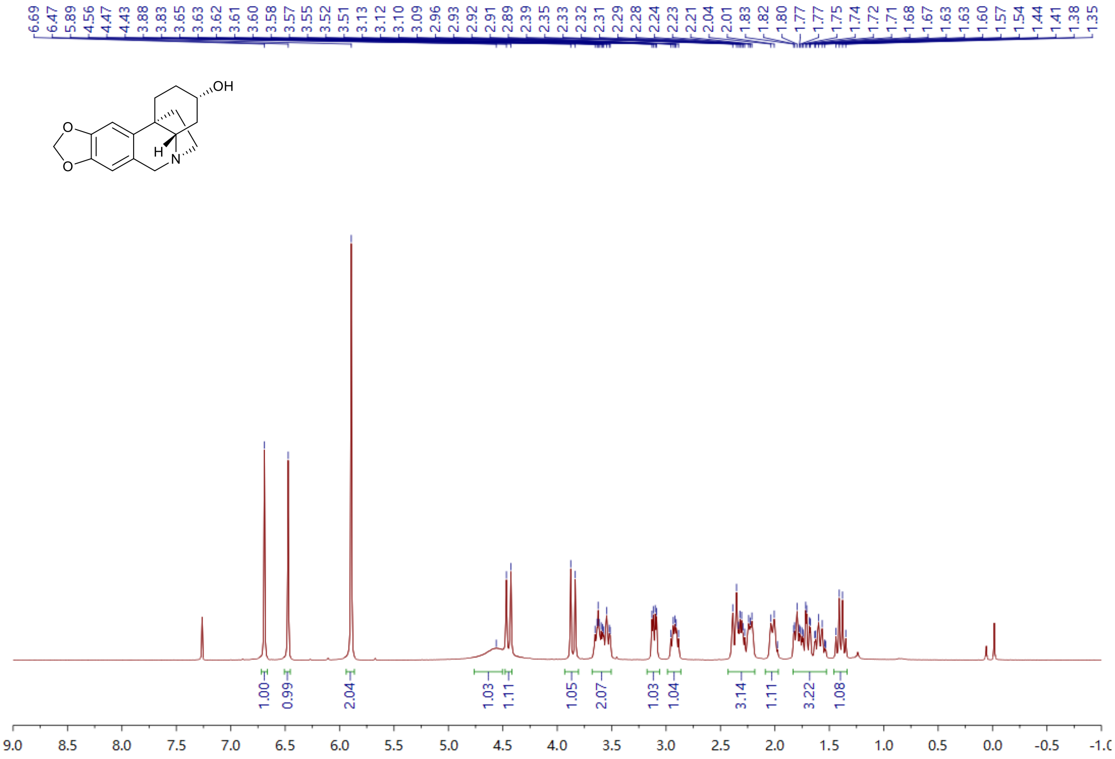

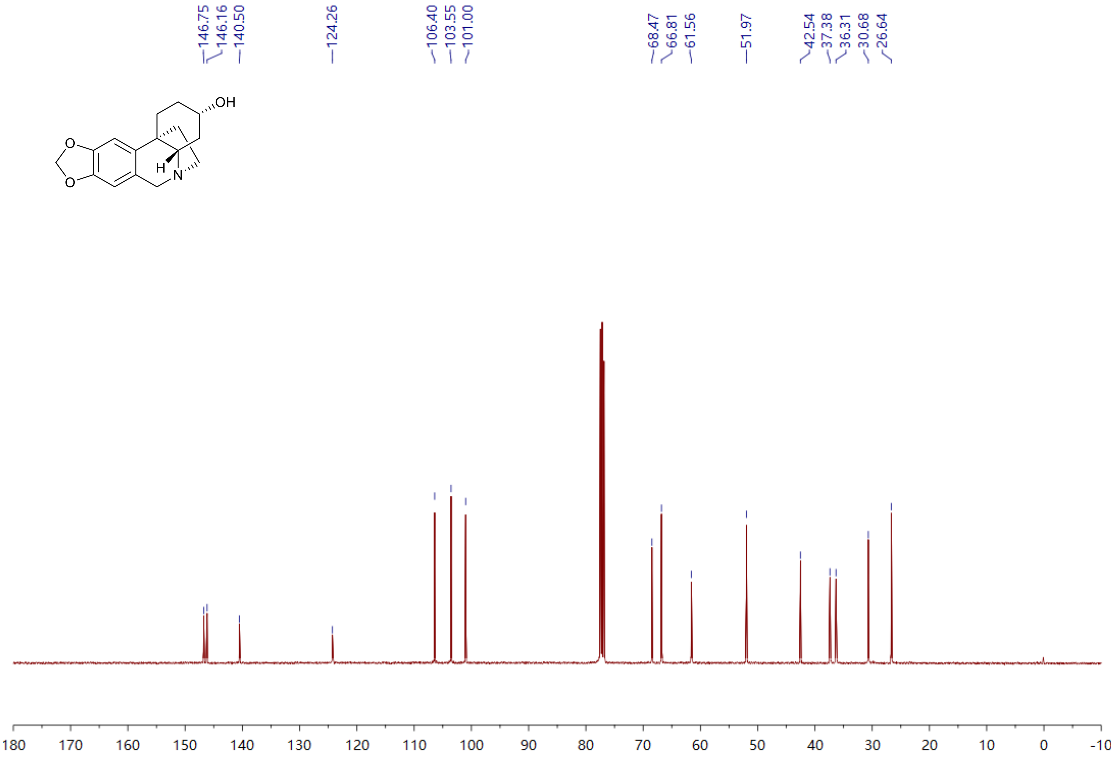

(–)-8-*O*-Demethylmartidine

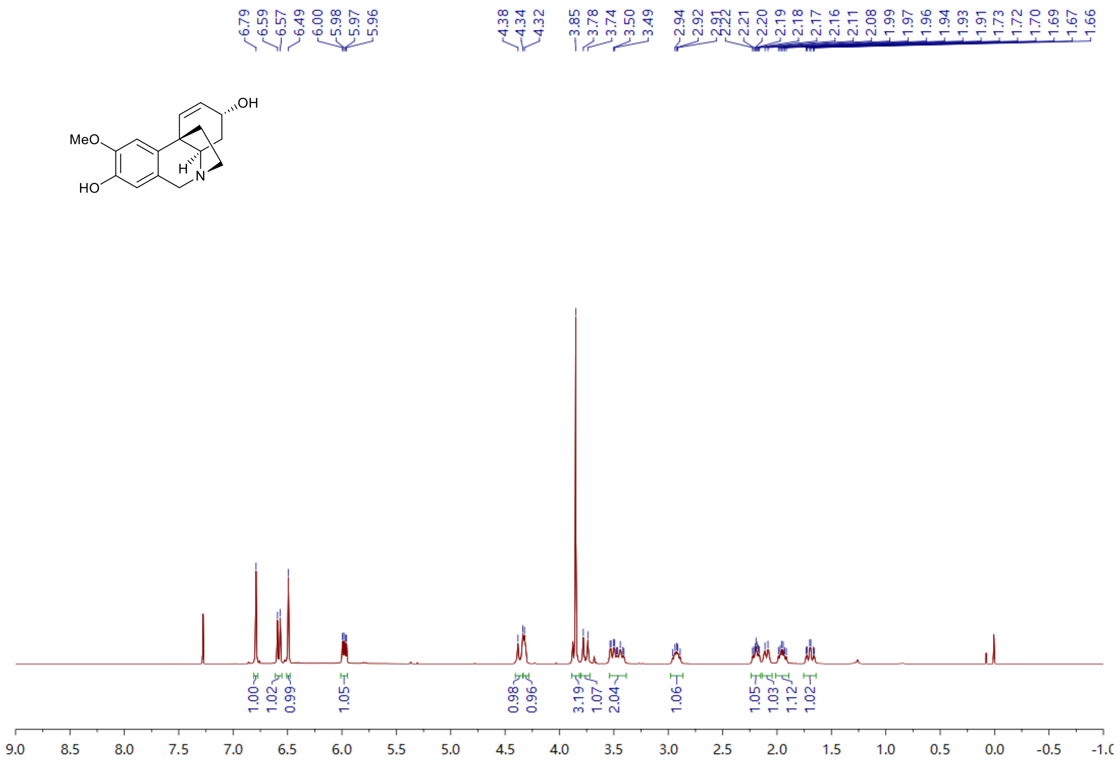

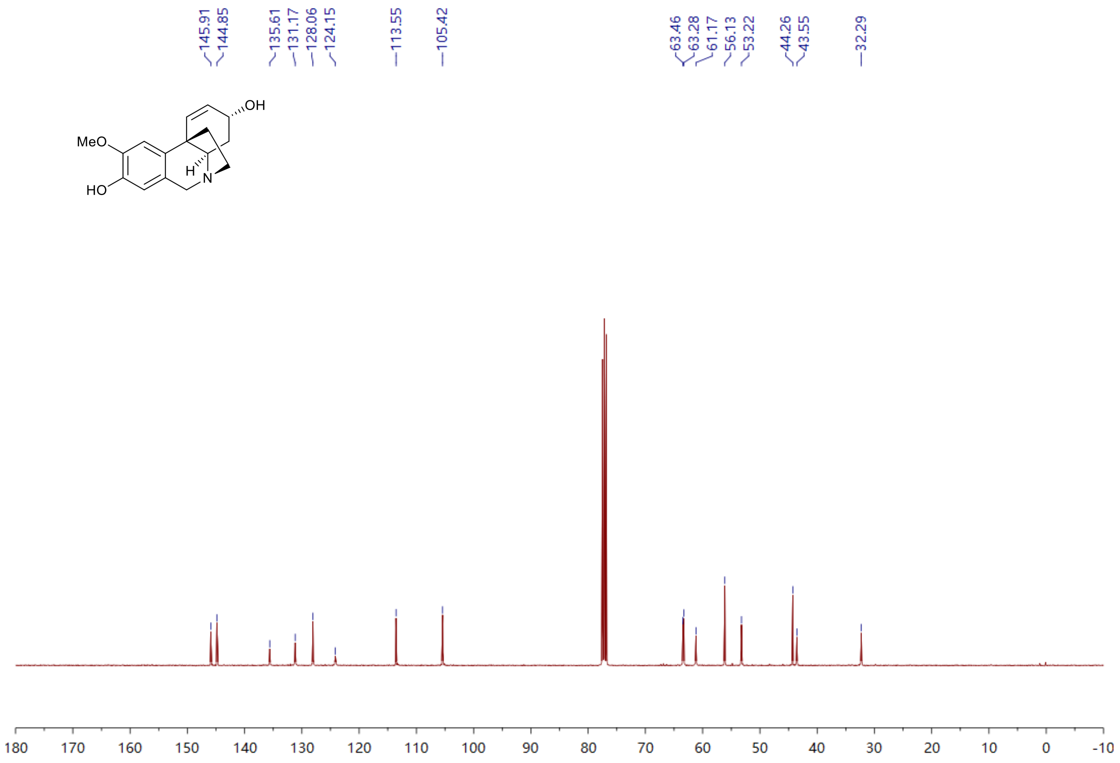

(+)-Siculine

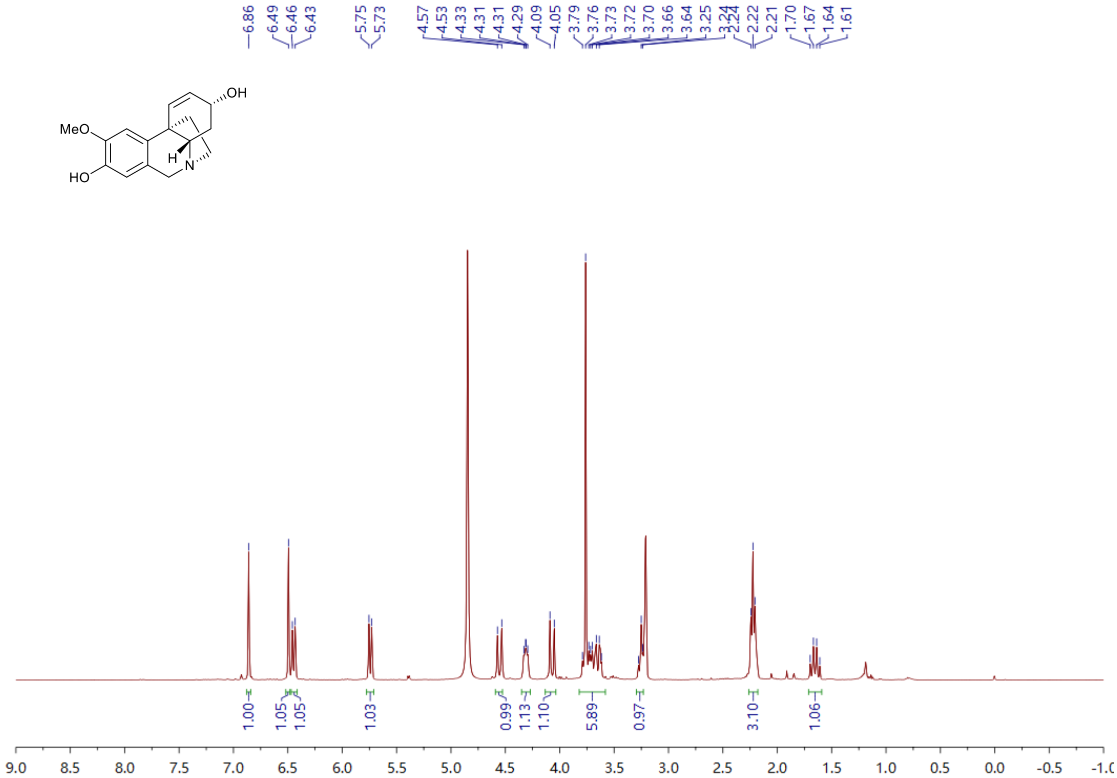

6.86
6.49
6.46
6.43
5.75
5.73
4.57
4.53
4.33
4.31
4.31
4.29
4.09
4.05
3.79
3.76
3.73
3.72
3.70
3.66
3.64
3.25
3.24
2.24
2.22
2.21
1.70
1.67
1.64
1.61
MeO
HO
OH
H
N
1.00
1.05
1.05
1.03
0.99
1.13
1.10
5.89
0.97
3.10
1.06
9.0
8.5
8.0
7.5
7.0
6.5
6.0
5.5
5.0
4.5
4.0
3.5
3.0
2.5
2.0
1.5
1.0
0.5
0.0
-0.5

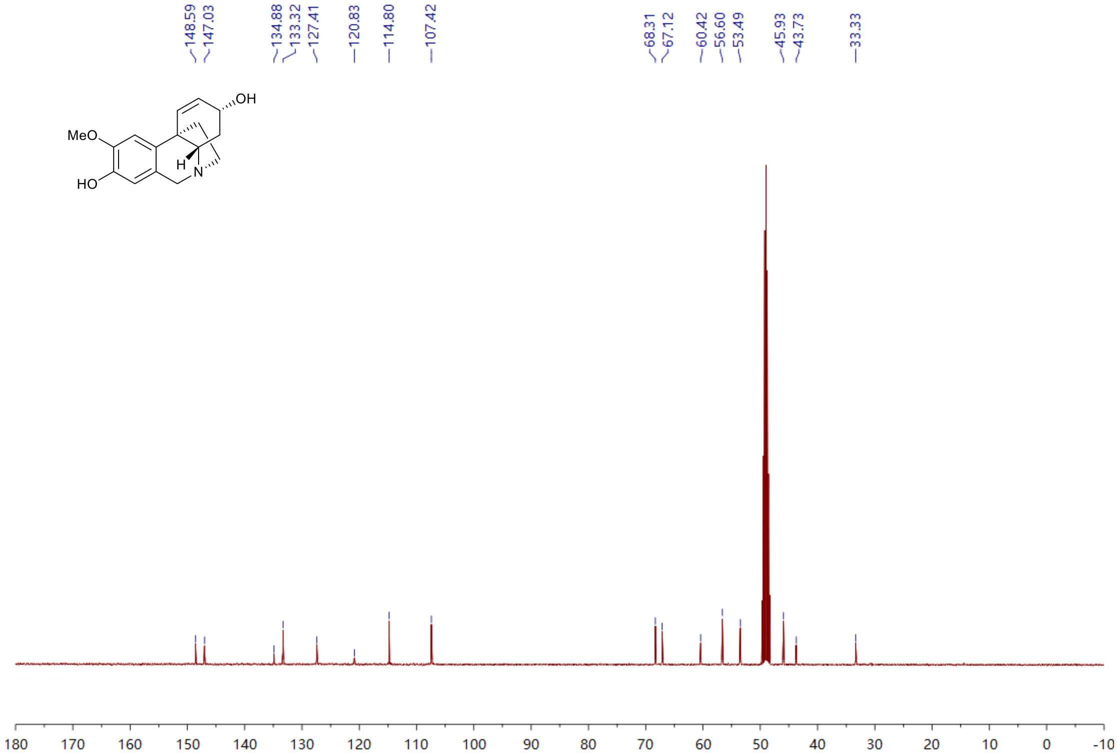

148.59
147.03
134.88
133.32
127.41
120.83
114.80
107.42
68.31
67.12
60.42
56.60
53.49
45.93
43.73
33.33
MeO
HO
OH
H
N
180
170
160
150
140
130
120
110
100
90
80
70
60
50
40
30
20
10
0
-10

(–)-Buphanisine

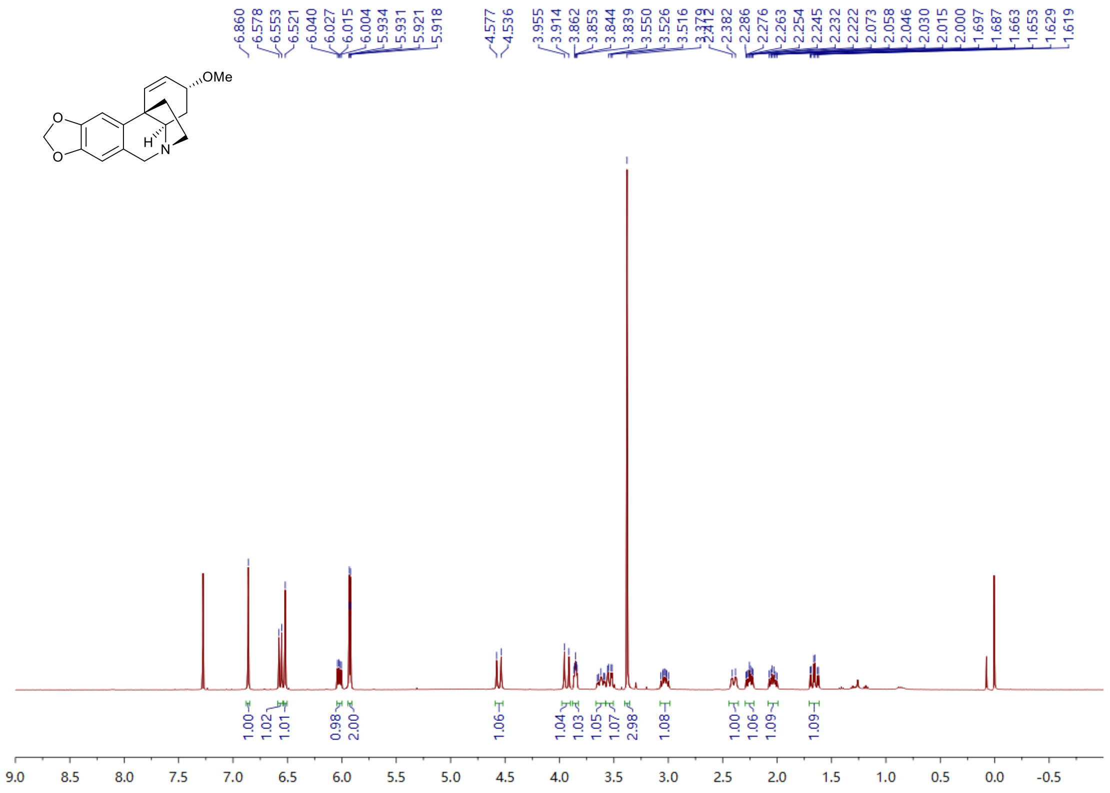

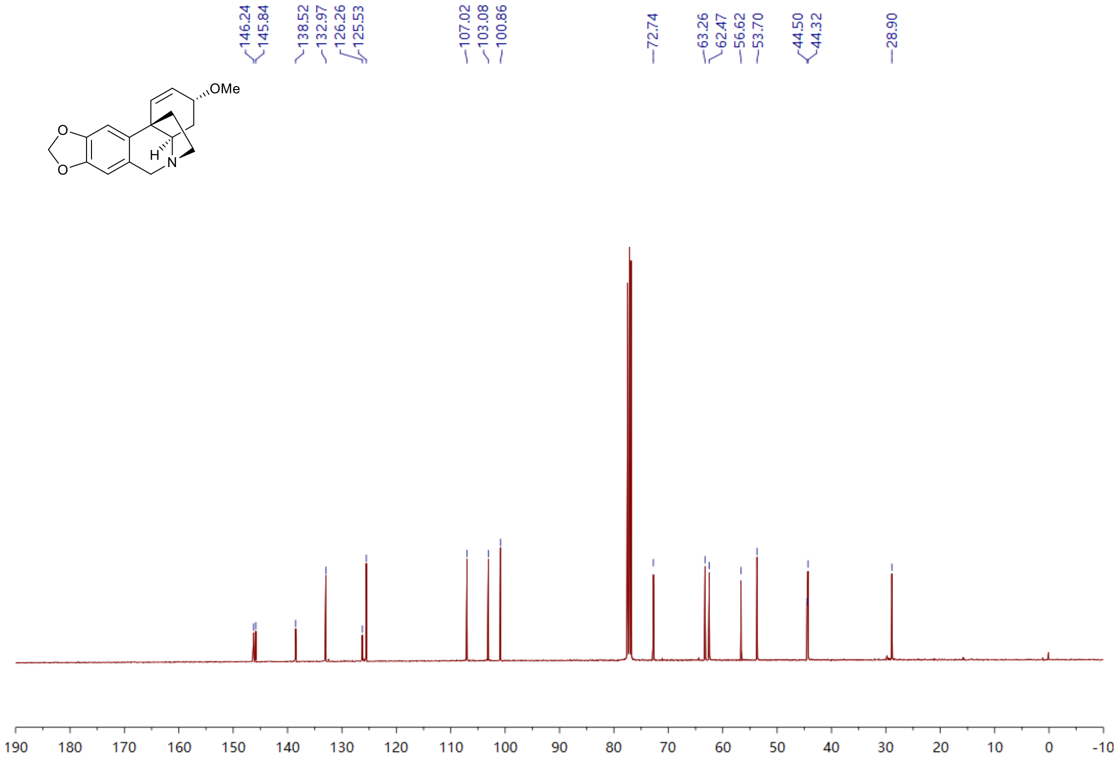

(–)-Buphanidrine

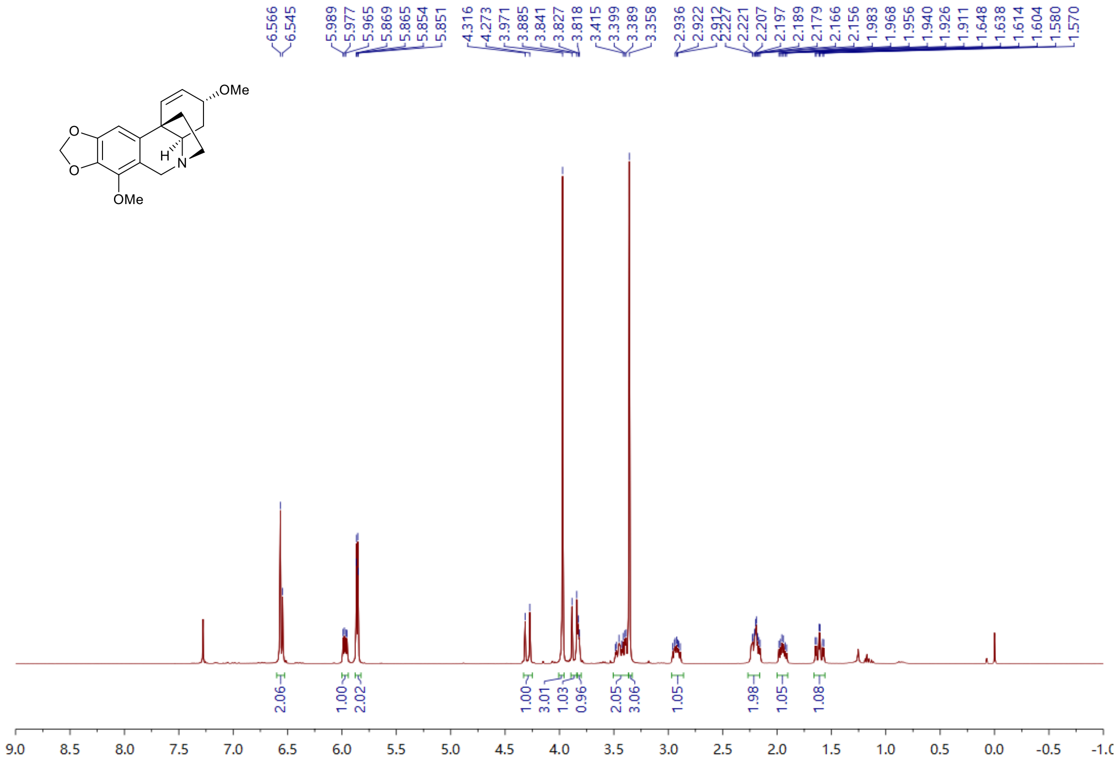

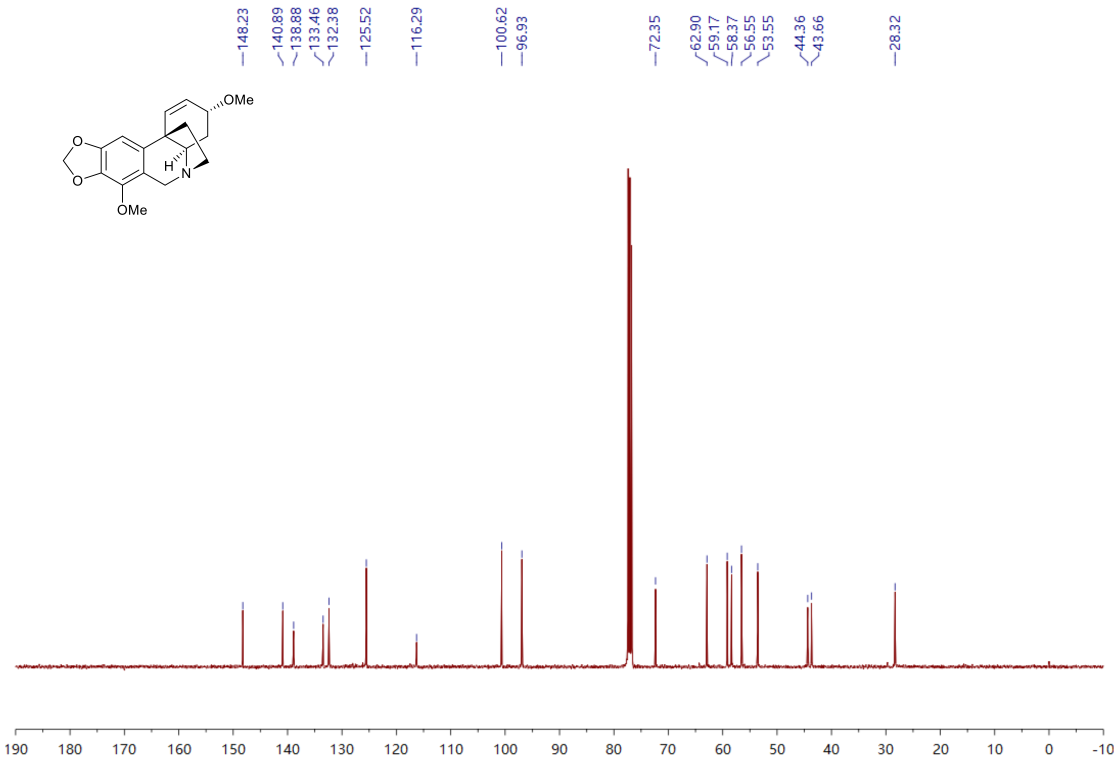

Compound 4

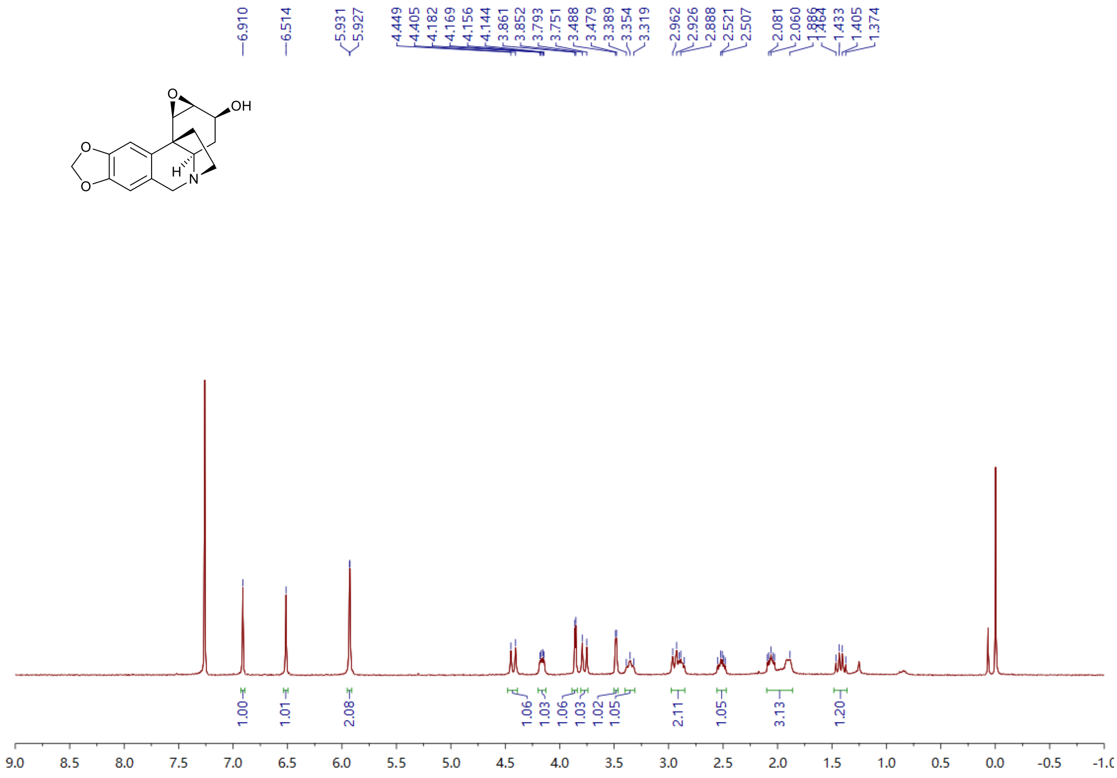

6.910
6.514
5.931
5.927
4.449
4.405
4.182
4.169
4.156
4.144
3.861
3.852
3.793
3.751
3.488
3.479
3.389
3.354
3.319
2.962
2.926
2.888
2.521
2.507
2.081
2.060
1.433
1.405
1.374
OH
O
O
O
H
N
1.00
1.01
2.08
1.06
1.03
1.06
1.03
1.02
1.05
2.11
1.05
3.13
1.20
9.0 8.5 8.0 7.5 7.0 6.5 6.0 5.5 5.0 4.5 4.0 3.5 3.0 2.5 2.0 1.5 1.0 0.5 0.0 -0.5

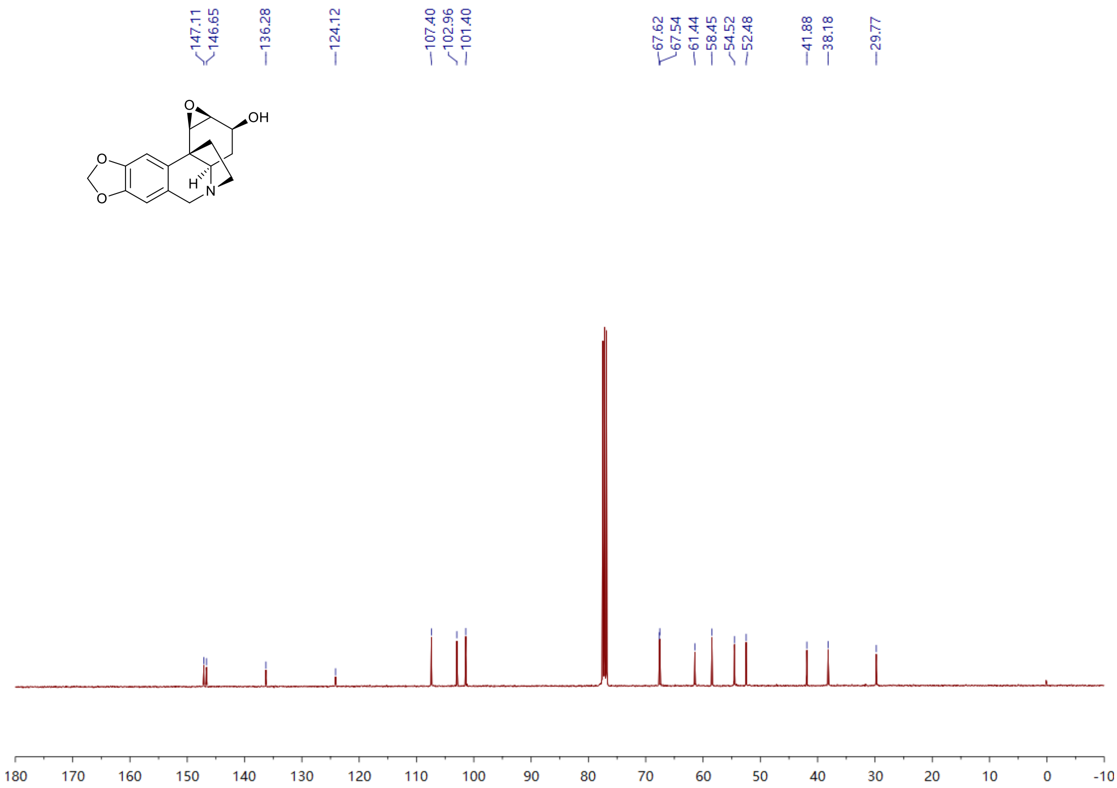

147.11
146.65
136.28
124.12
107.40
102.96
101.40
67.62
67.54
61.44
58.45
54.52
52.48
41.88
38.18
29.77
OH
O
O
O
H
N
180 170 160 150 140 130 120 110 100 90 80 70 60 50 40 30 20 10 0 -10

(–)-Flexinine

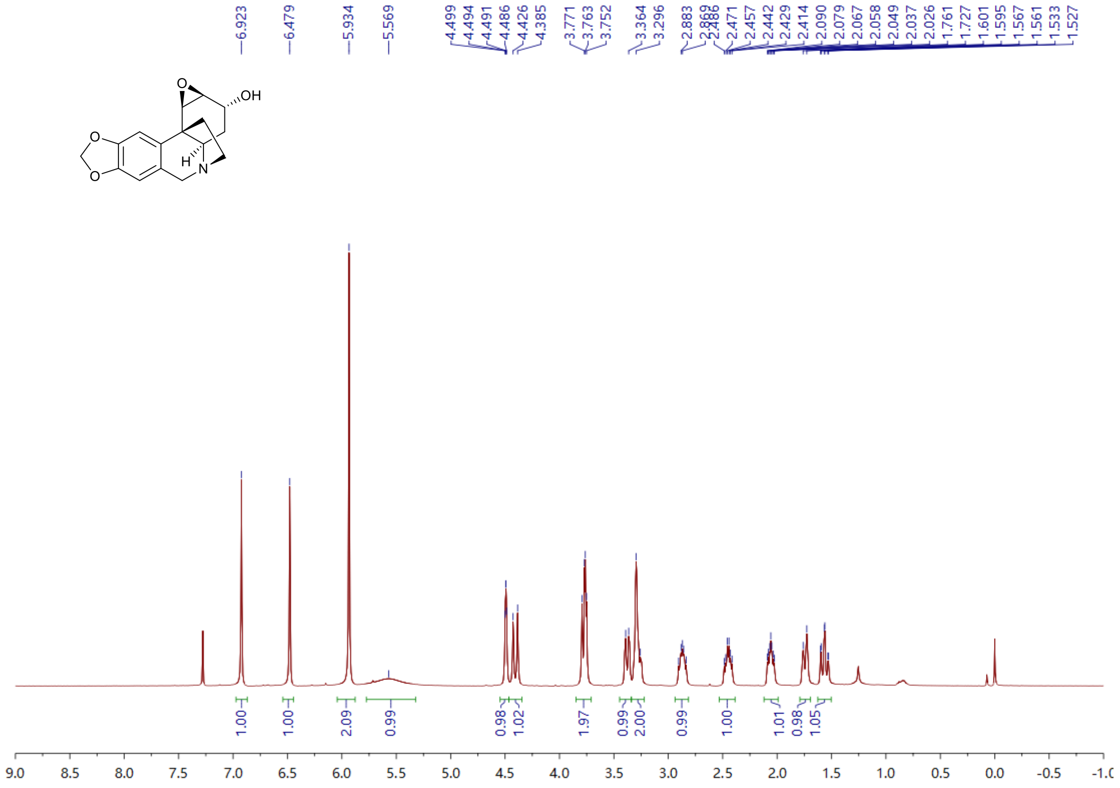

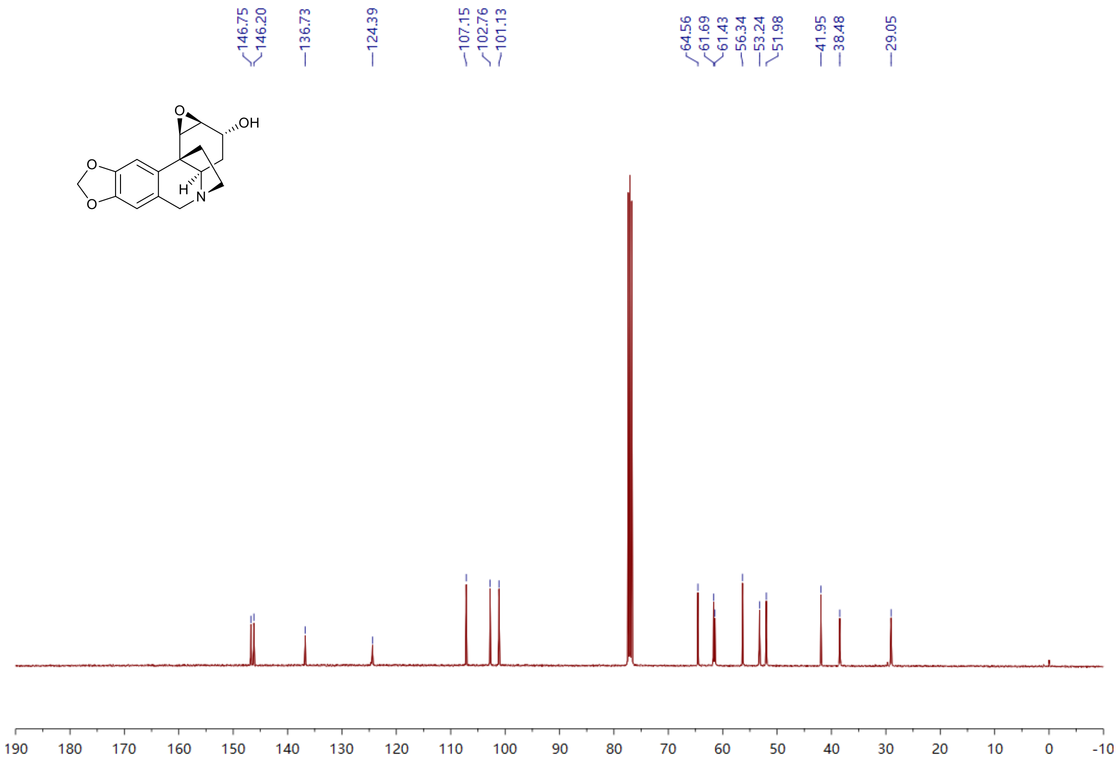

2D NOESY spectrum of (−)-Flexinine

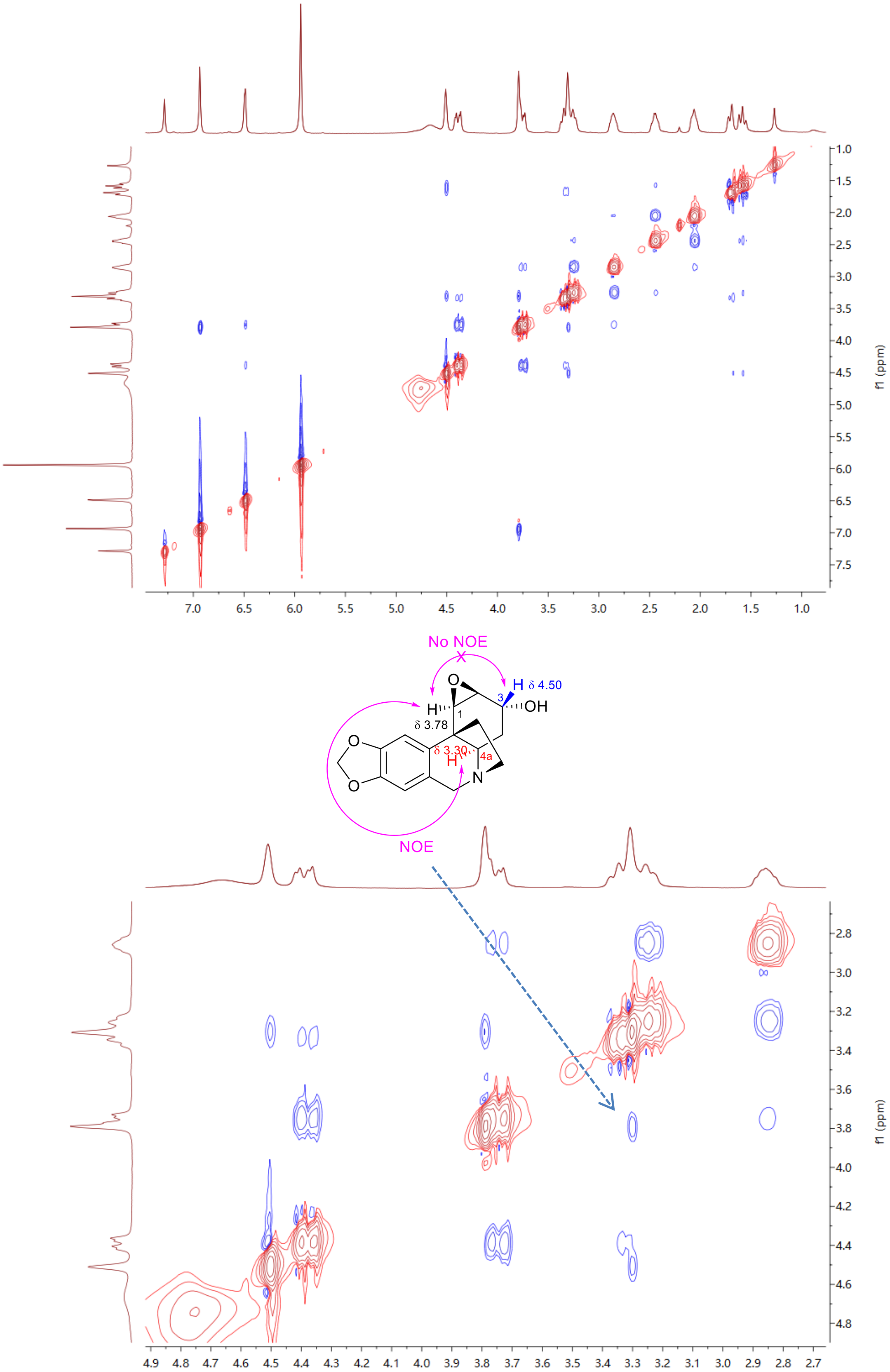

(–)-Augustine

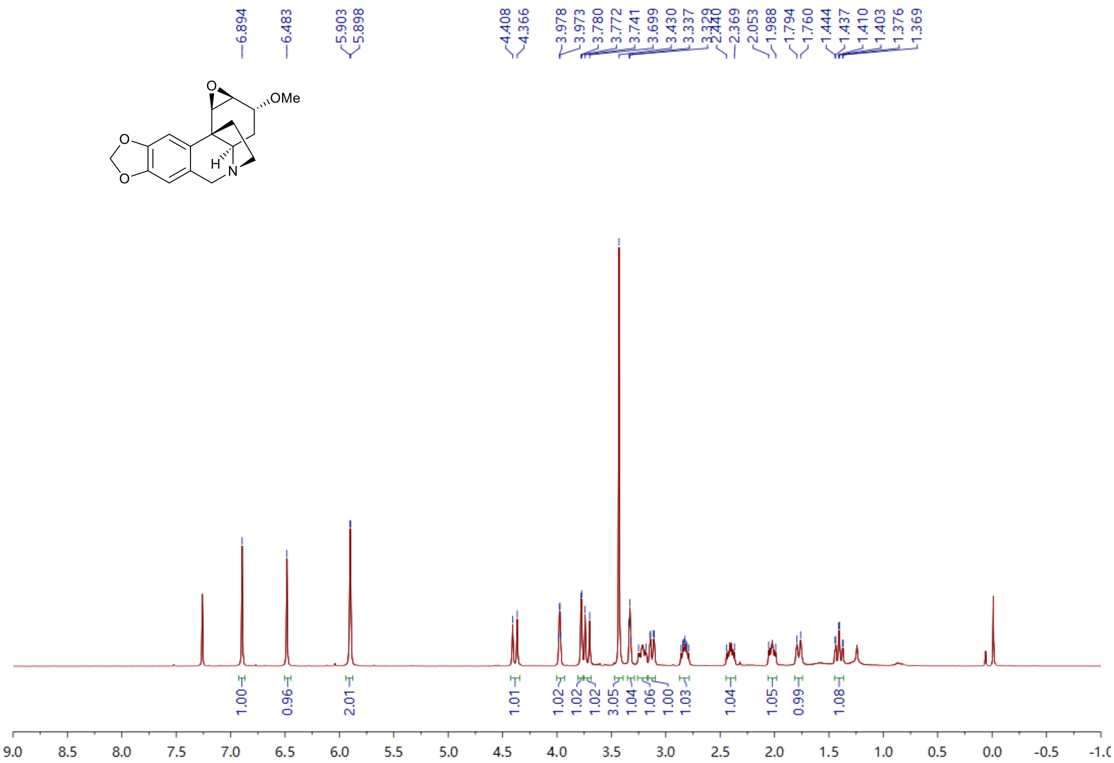

6.894
6.483
5.903
5.898
4.408
4.366
3.978
3.973
3.780
3.772
3.741
3.699
3.430
3.337
2.369
2.053
1.988
1.794
1.760
1.444
1.437
1.410
1.403
1.376
1.369
OMe
H
N
1.00
0.96
2.01
1.01
1.02
1.02
1.02
3.05
1.04
1.06
1.00
1.03
1.04
1.05
0.99
1.08

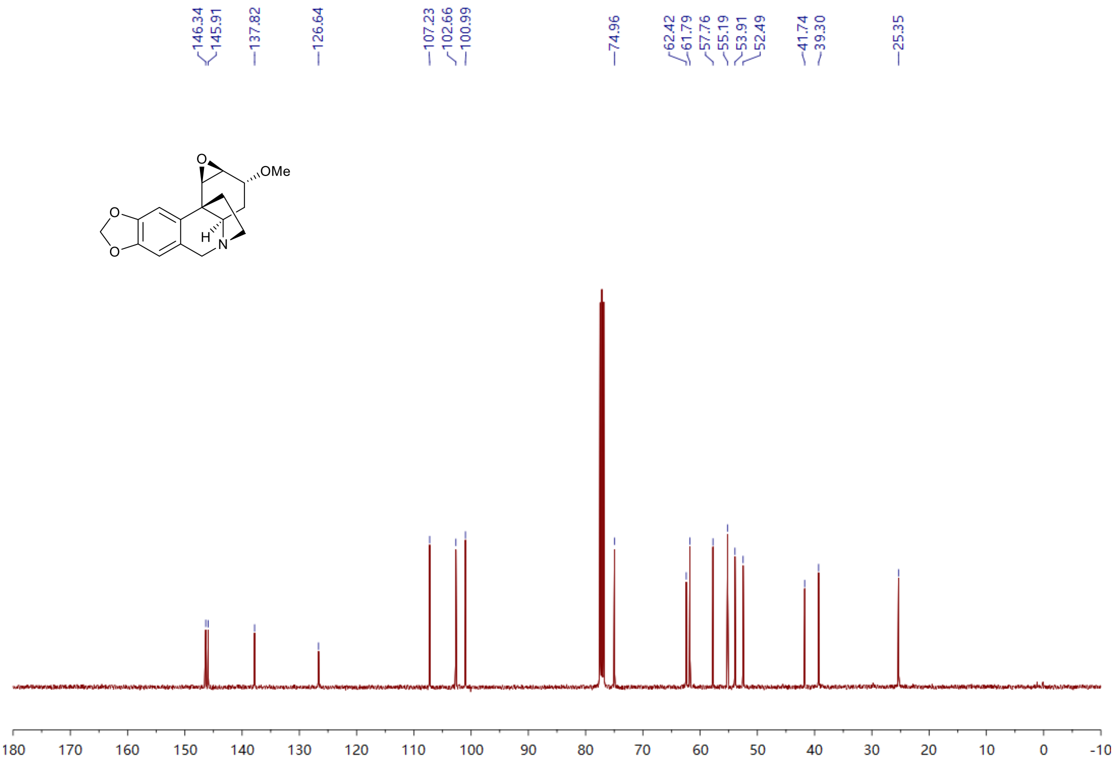

146.34
145.91
137.82
126.64
107.23
102.66
100.99
74.96
62.42
61.79
57.76
55.19
53.91
52.49
41.74
39.30
25.35
OMe
H
N

(H) HPLC Charts for Asymmetric Hydrogenation Products

(-)-crinine ((-)-*cis*-2a) and (+)-vittatine ((+)-*cis*-2a)

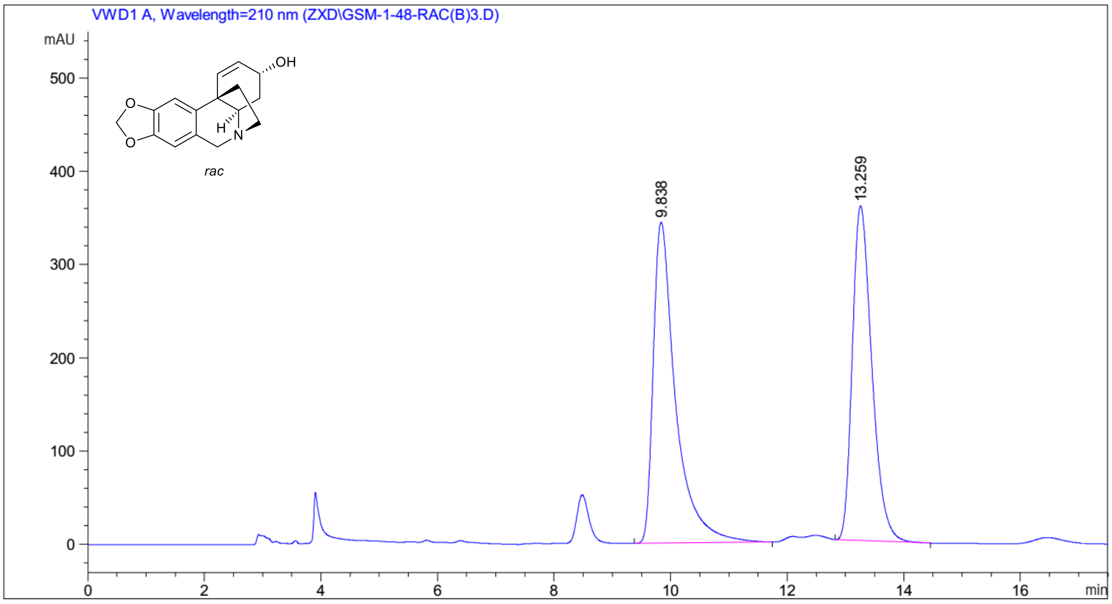

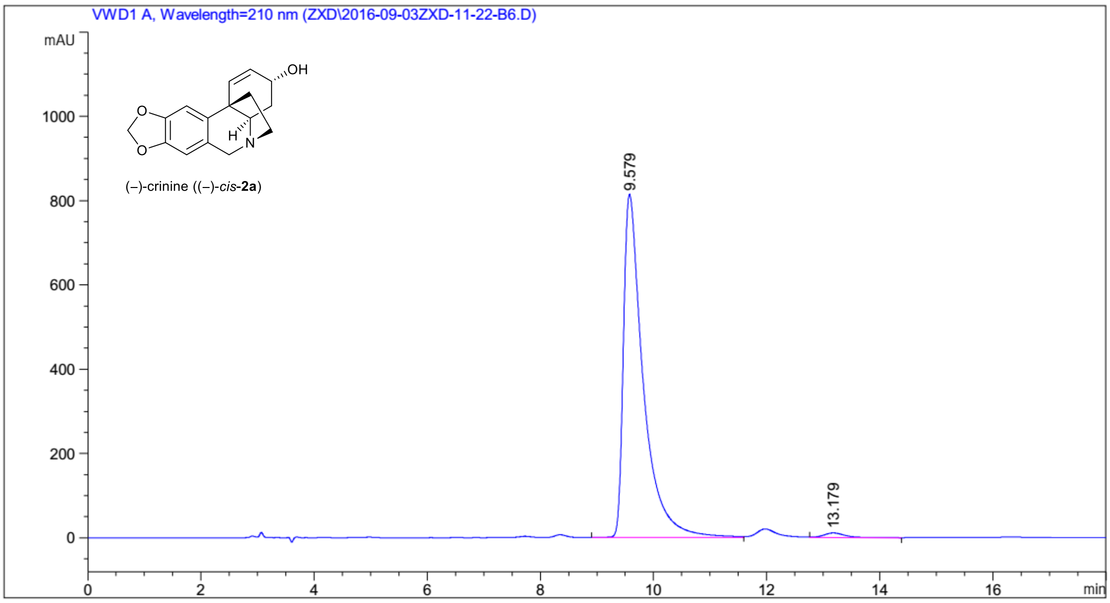

Peak #	RetTime [min]	Type	Width [min]	Area [mAU*s]	Height [mAU]	Area %
1	9.579	BV	0.3529	1.98582e4	813.17267	98.4793
2	13.179	VB	0.4120	306.65115	11.18066	1.5207

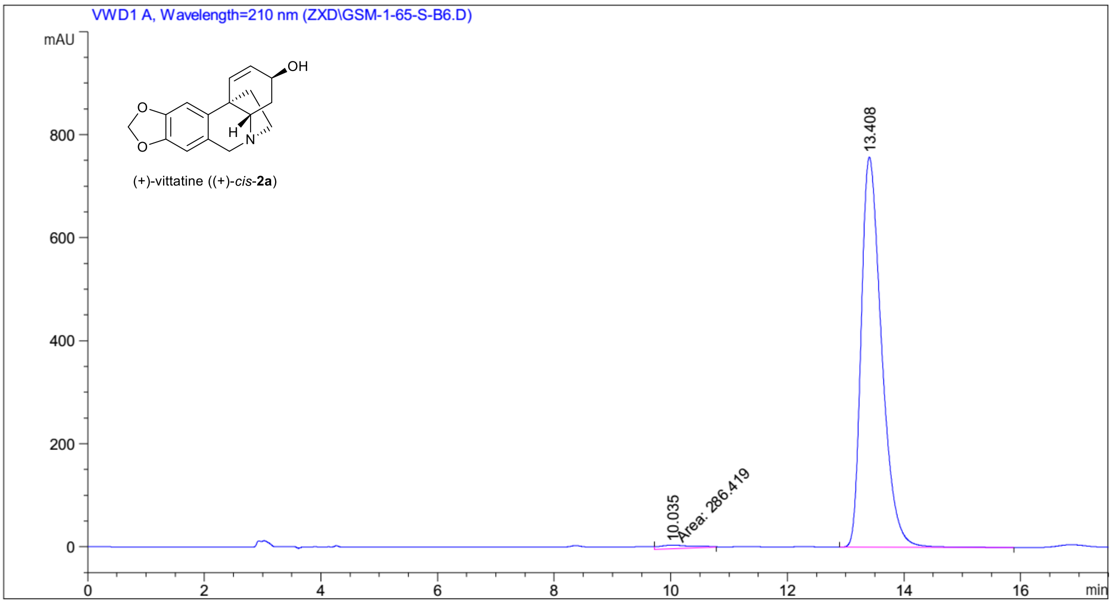

Peak #	RetTime [min]	Type	Width [min]	Area [mAU*s]	Height [mAU]	Area %
1	10.035	MM	0.6620	286.41934	7.21067	1.5341
2	13.408	VB	0.3712	1.83832e4	757.21887	98.4659

(+)-epivittatine ((+)-*trans*-2a) and (-)-epicrinine ((-)-*trans*-2a)

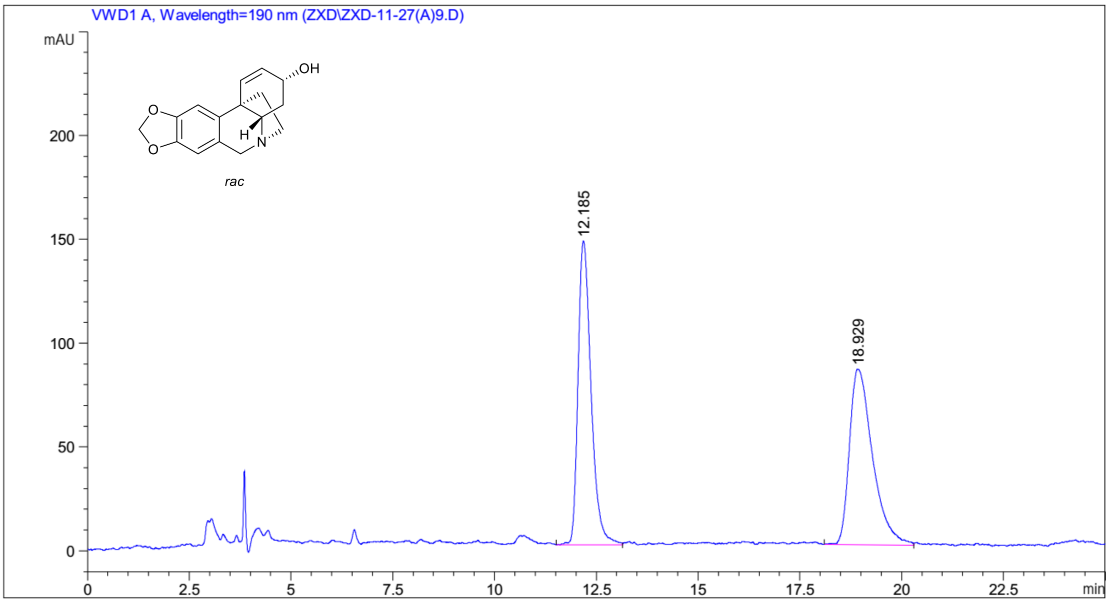

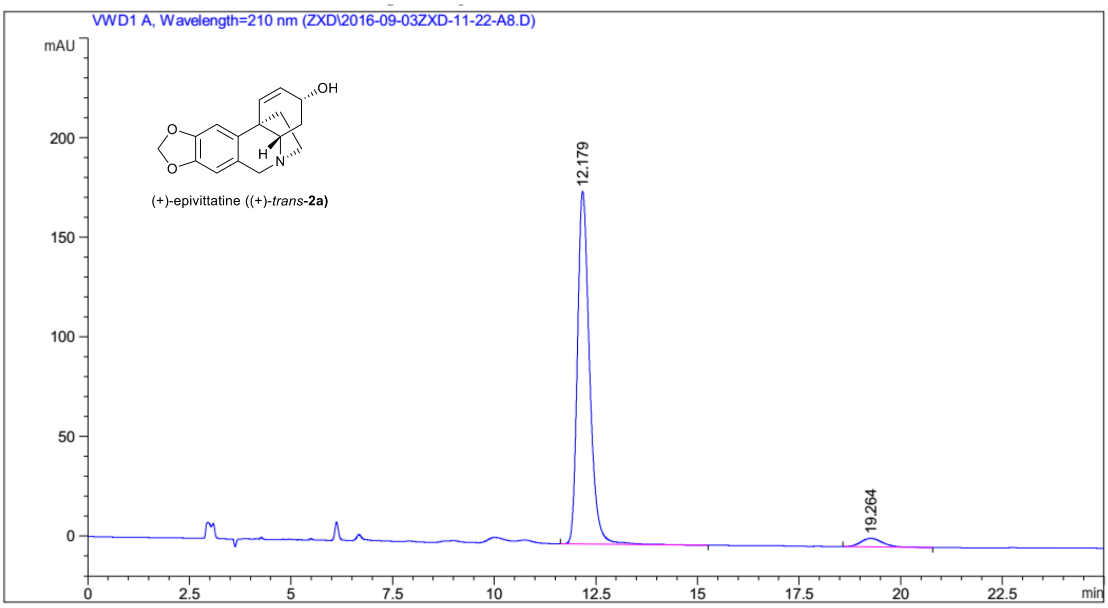

Peak #	RetTime [min]	Type	Width [min]	Area [mAU*s]	Height [mAU]	Area %
1	12.179	BV	0.3183	3676.11865	176.98756	95.8359
2	19.264	BB	0.5811	159.72794	4.26344	4.1641

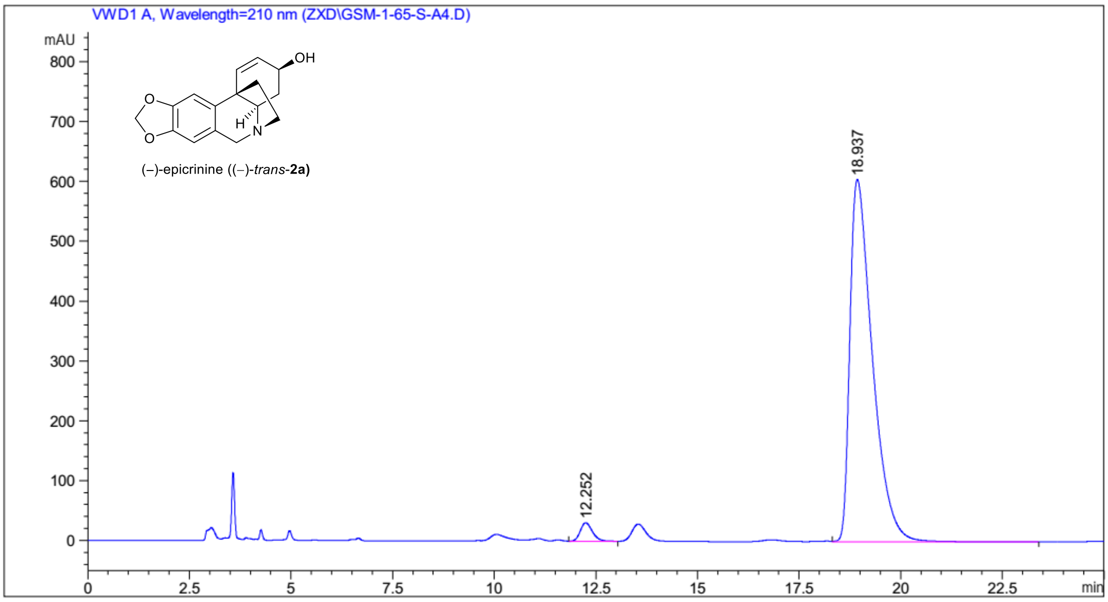

Peak #	RetTime [min]	Type	Width [min]	Area [mAU*s]	Height [mAU]	Area %
1	12.252	BV	0.3233	645.18146	30.93937	2.6958
2	18.937	VB	0.5808	2.32877e4	605.39636	97.3042

(-)-maritidine ((-)-*cis*-2b) and (+)-maritidine((+)-*cis*-2b)

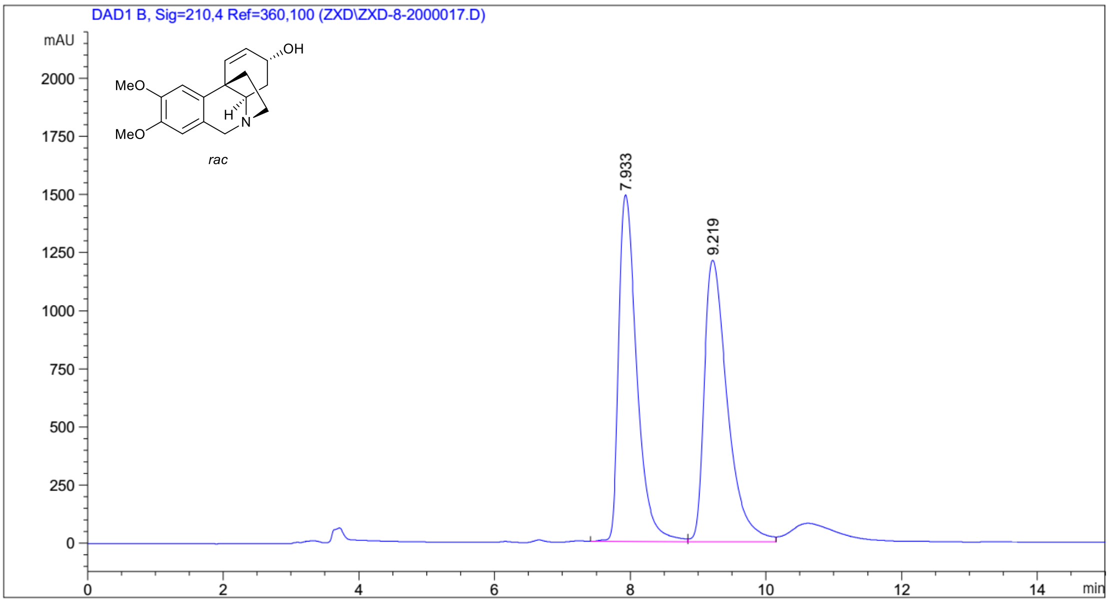

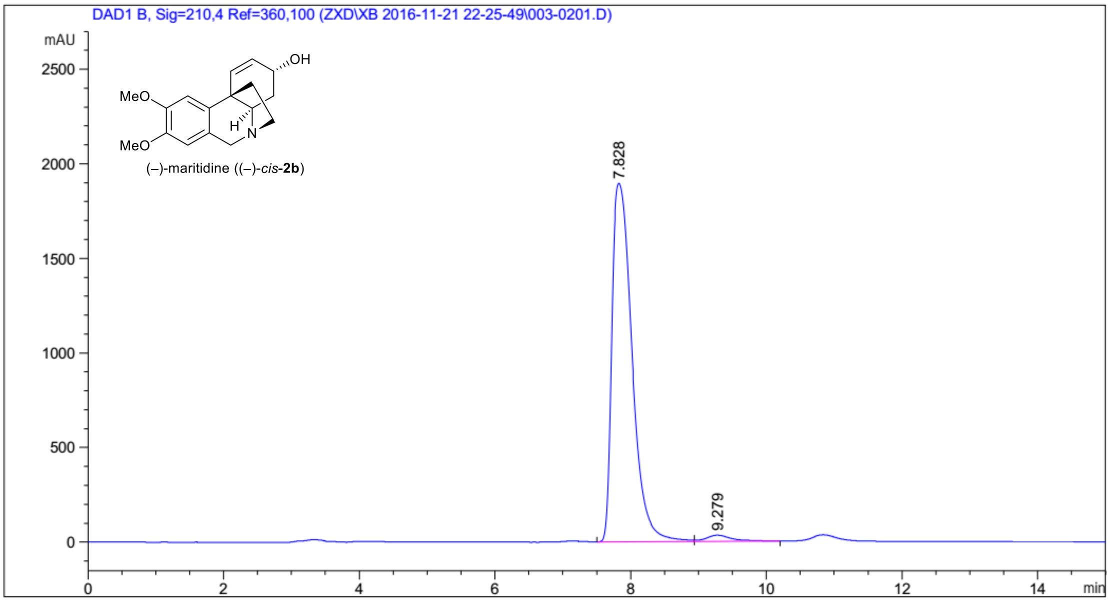

Peak #	RetTime [min]	Type	Width [min]	Area [mAU*s]	Height [mAU]	Area %
1	7.828	BV	0.3223	3.90479e4	1895.90833	97.7862
2	9.279	VB	0.3965	884.00391	33.00588	2.2138

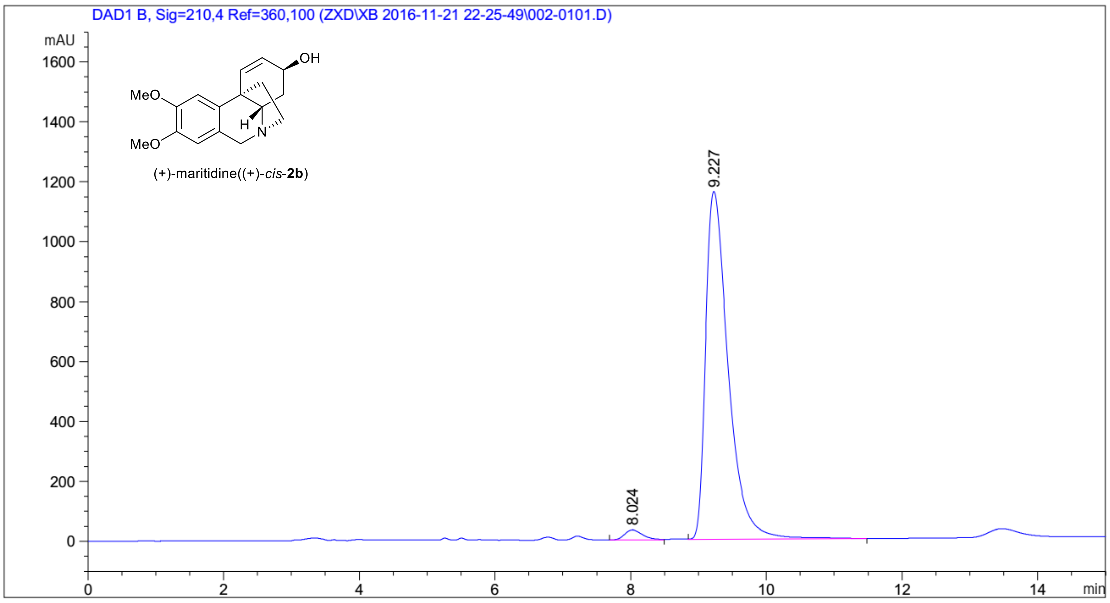

Peak #	RetTime [min]	Type	Width [min]	Area [mAU*s]	Height [mAU]	Area %
1	8.024	BV	0.2821	606.55884	33.36724	2.2038
2	9.227	VB	0.3524	2.69170e4	1161.54883	97.7962

(+)-epimaritidine ((+)-*trans*-2b) and (-)-epimaritidine ((-)-*trans*-2b)

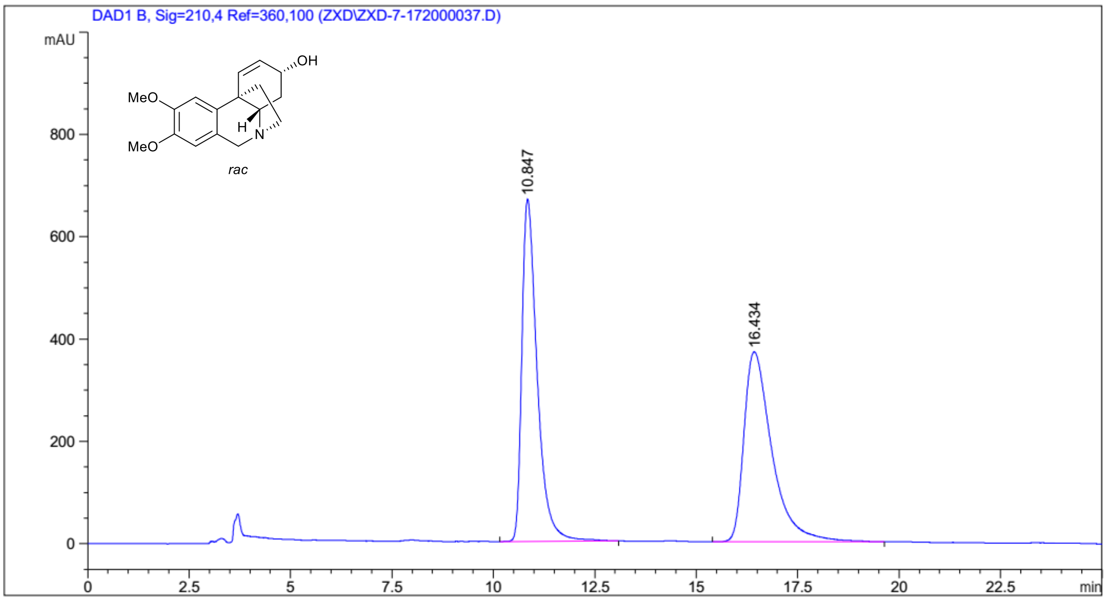

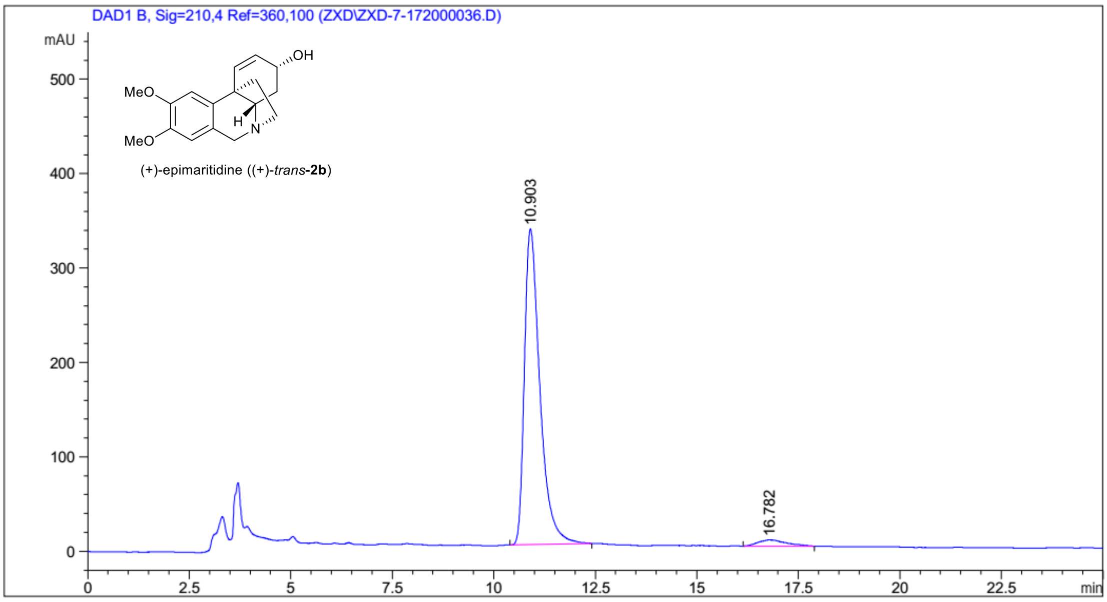

Peak #	RetTime [min]	Type	Width [min]	Area [mAU*s]	Height [mAU]	Area %
1	10.903	BB	0.4016	8873.45801	334.37155	96.5033
2	16.782	BB	0.5815	321.51794	6.56398	3.4967

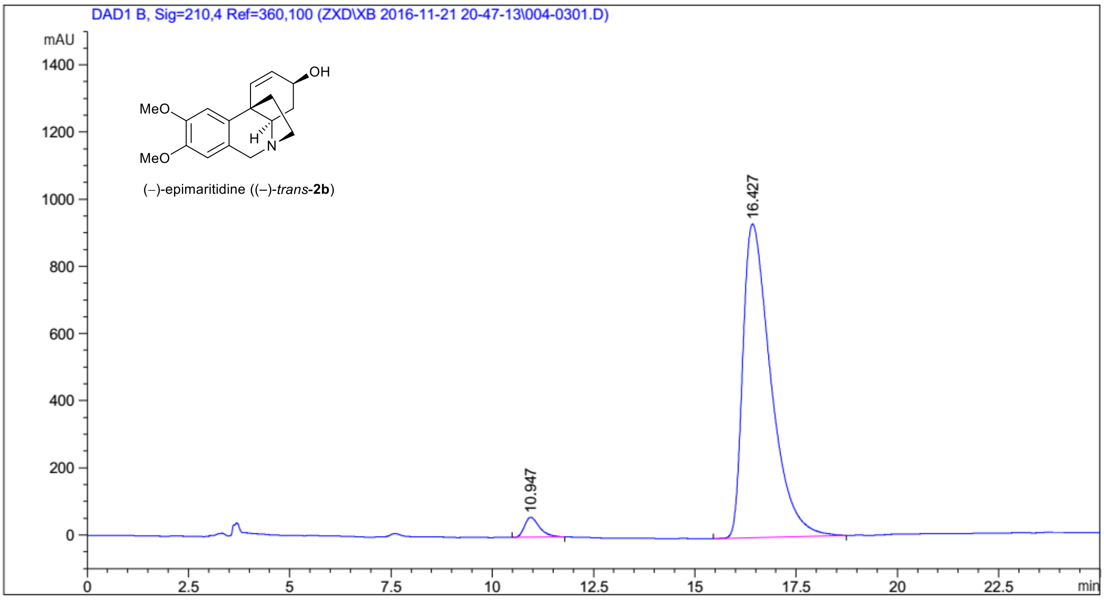

Peak #	RetTime [min]	Type	Width [min]	Area [mAU*s]	Height [mAU]	Area %
1	10.947	BB	0.3911	1541.03845	59.70542	3.3233
2	16.427	BB	0.7274	4.48295e4	934.92566	96.6767

(-)-*cis*-2c and (+)-*cis*-2c

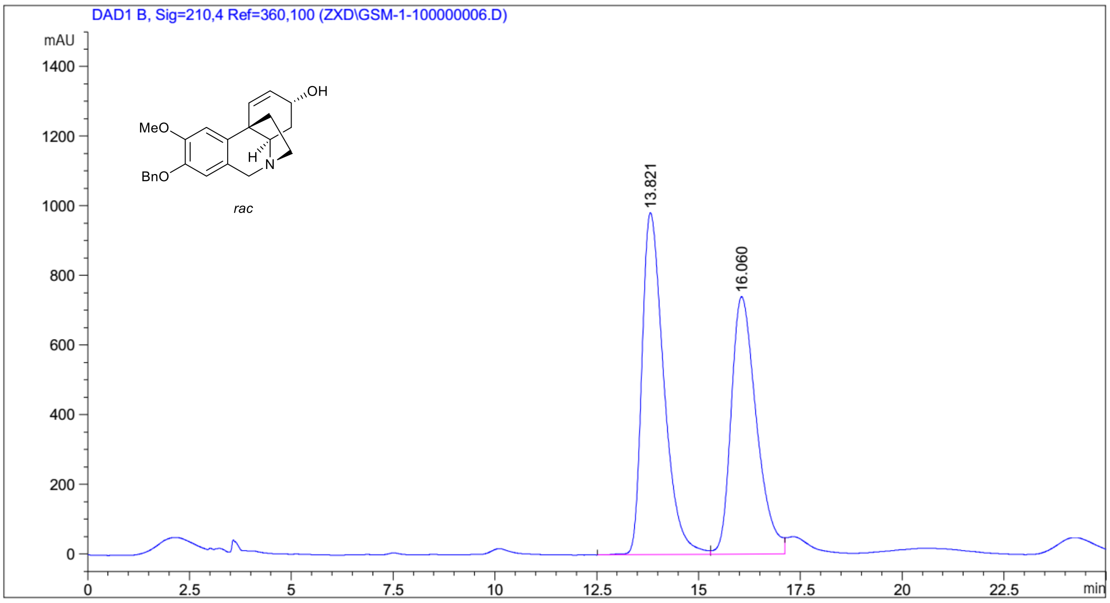

[illegible]

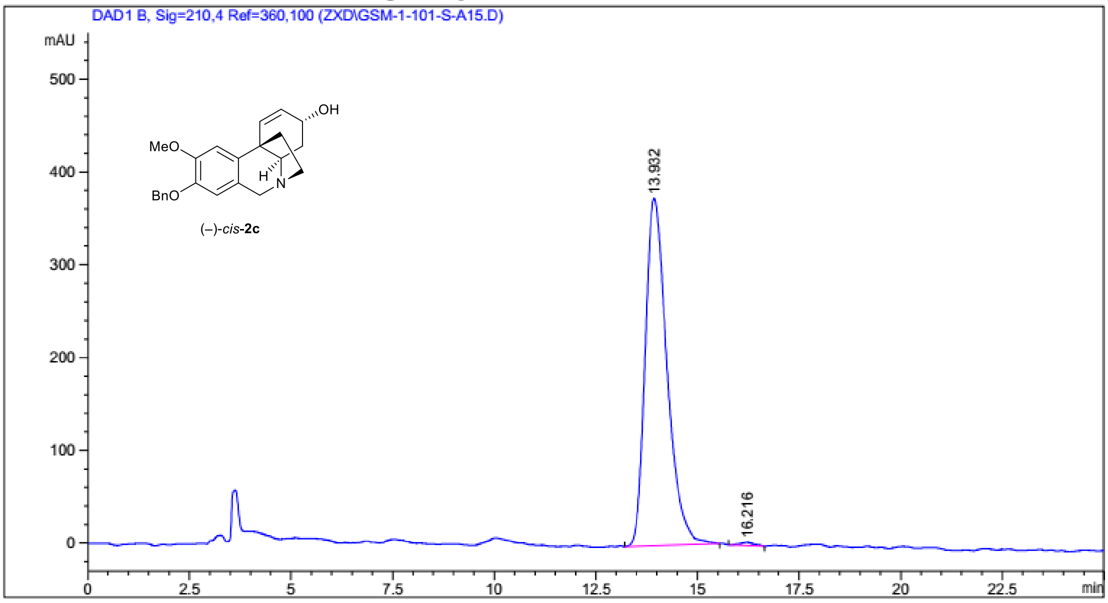

Peak #	RetTime [min]	Type	Width [min]	Area [mAU*s]	Height [mAU]	Area %
1	13.932	BB	0.5676	1.39697e4	374.11530	99.3815
2	16.216	BB	0.3204	86.93555	3.64293	0.6185

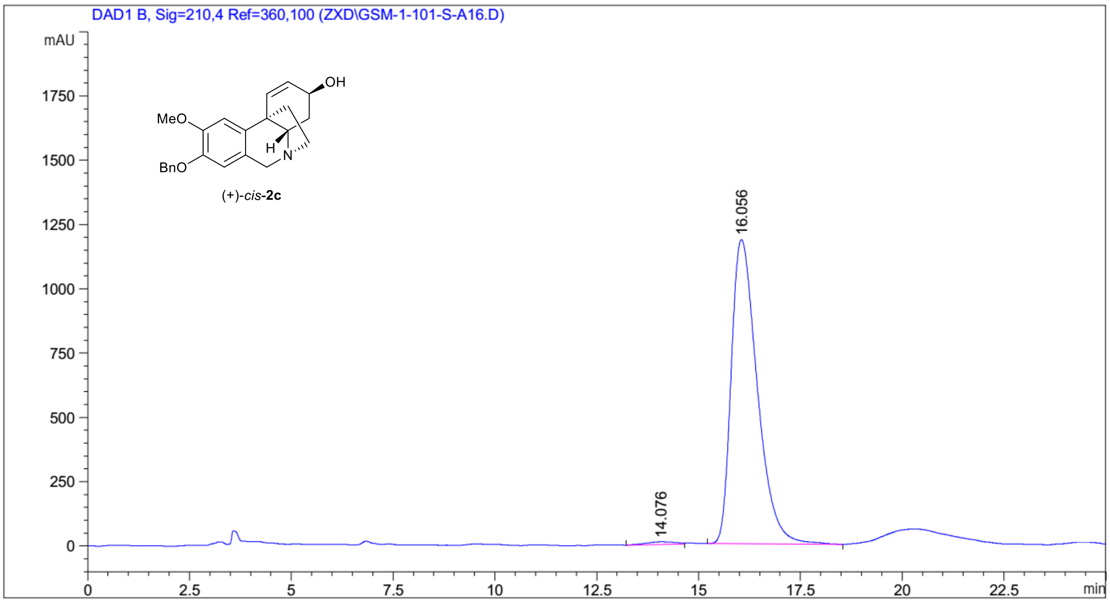

Peak #	RetTime [min]	Type	Width [min]	Area [mAU*s]	Height [mAU]	Area %
1	14.076	BV	0.6054	511.00635	10.36039	0.9614
2	16.056	BB	0.6811	5.26387e4	1183.00659	99.0386

(+)-*trans*-2c and (–)-*trans*-2c

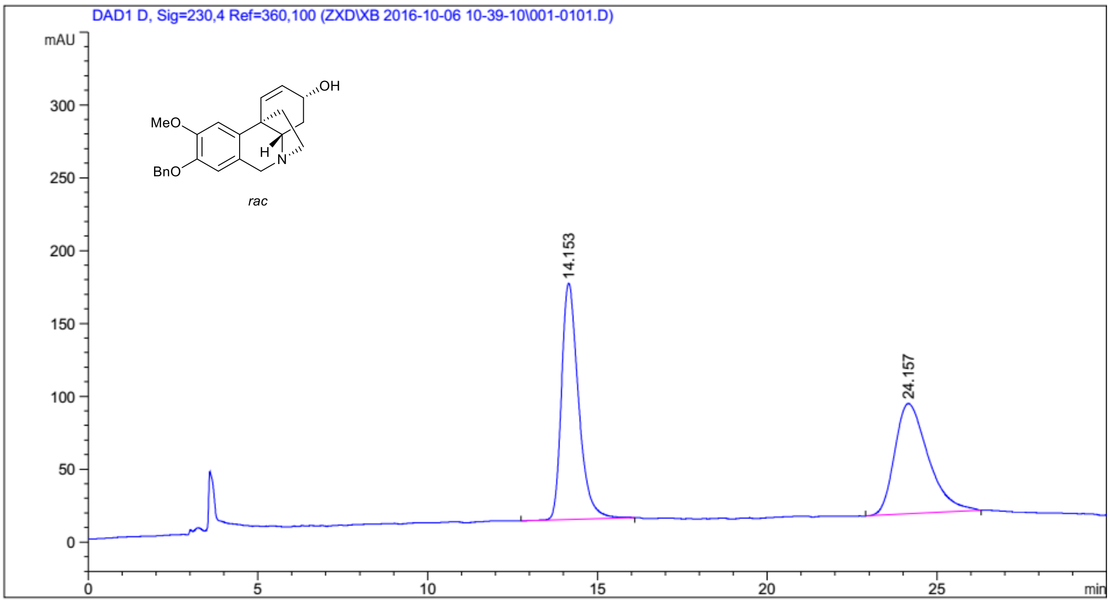

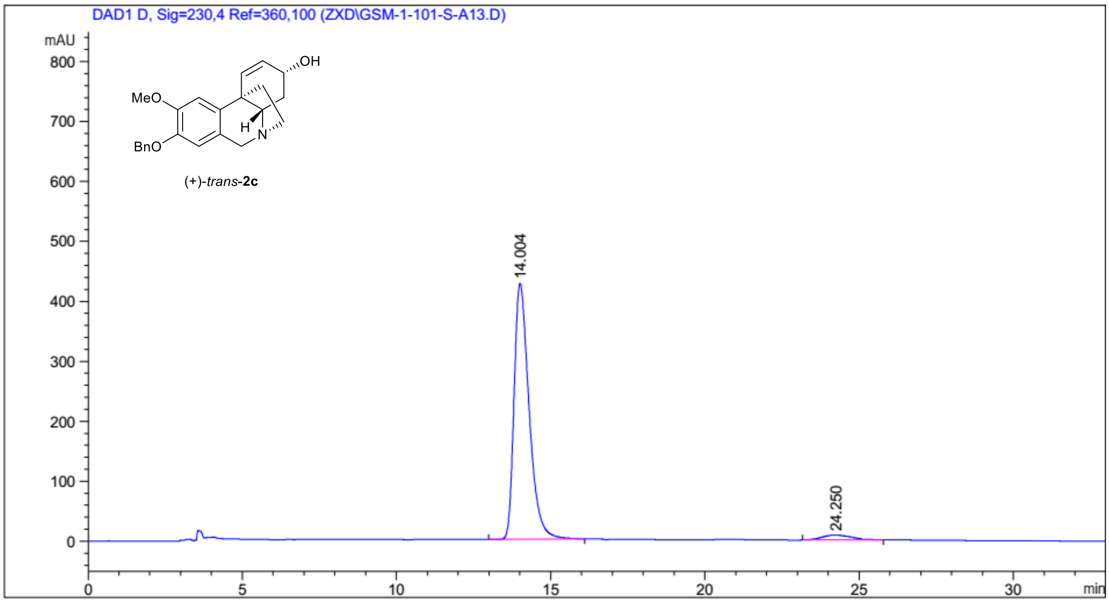

Peak #	RetTime [min]	Type	Width [min]	Area [mAU*s]	Height [mAU]	Area %
1	14.004	BB	0.5245	2.87670e4	842.11963	96.4060
2	24.246	BB	0.7918	1072.42151	16.33762	3.5940

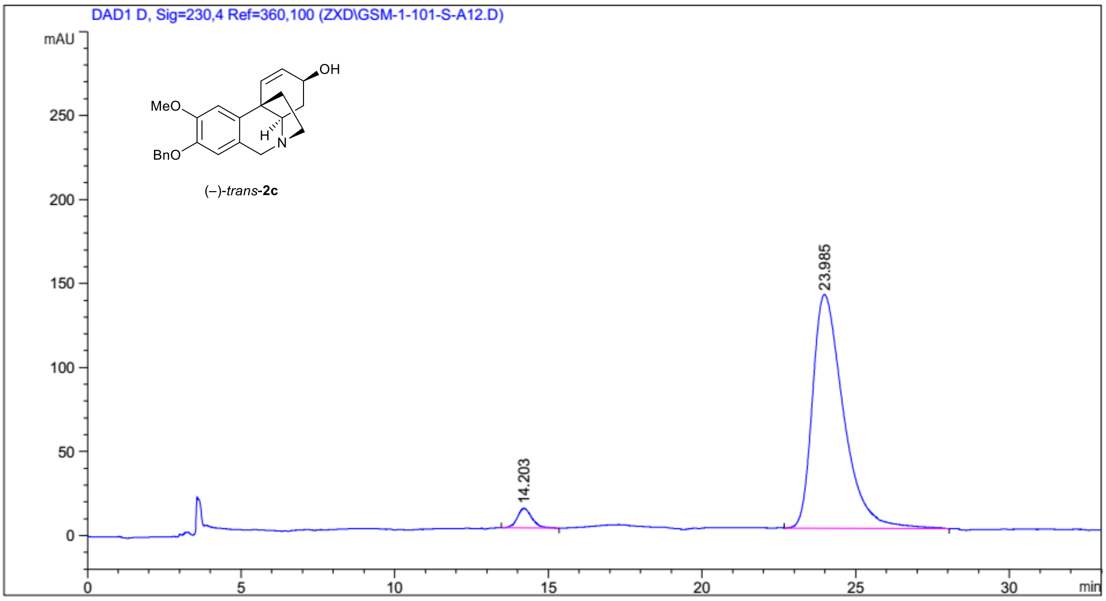

Peak #	RetTime [min]	Type	Width [min]	Area [mAU*s]	Height [mAU]	Area %
1	14.212	BB	0.4802	785.21466	23.60025	3.8383
2	23.985	BB	1.0374	1.96724e4	280.76617	96.1617

(-)-powelline ((-)-*cis*-2d) and (+)-powelline ((+)-*cis*-2d)

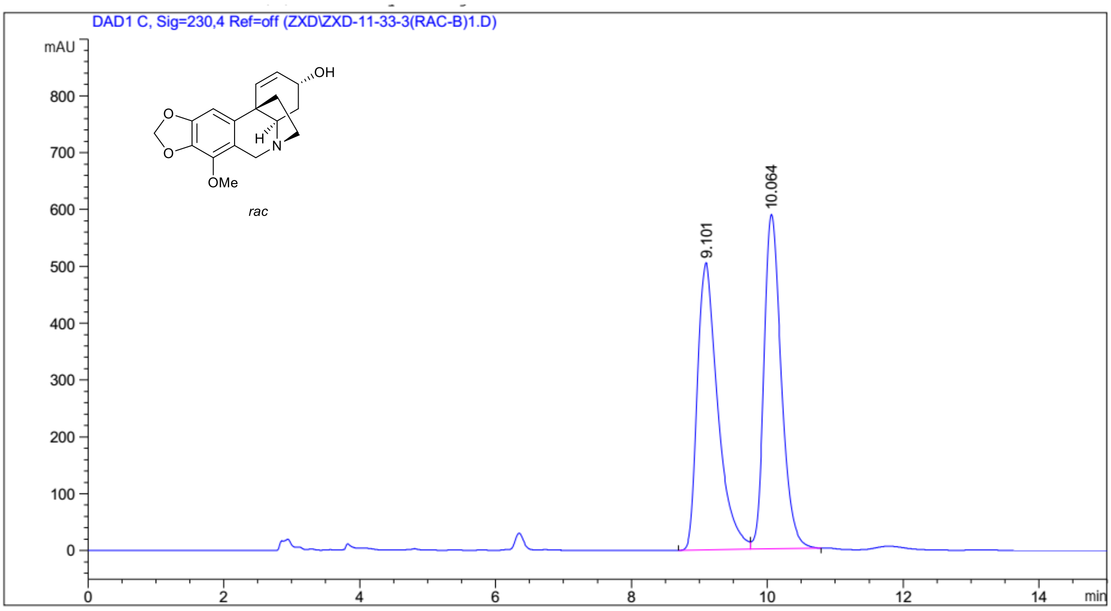

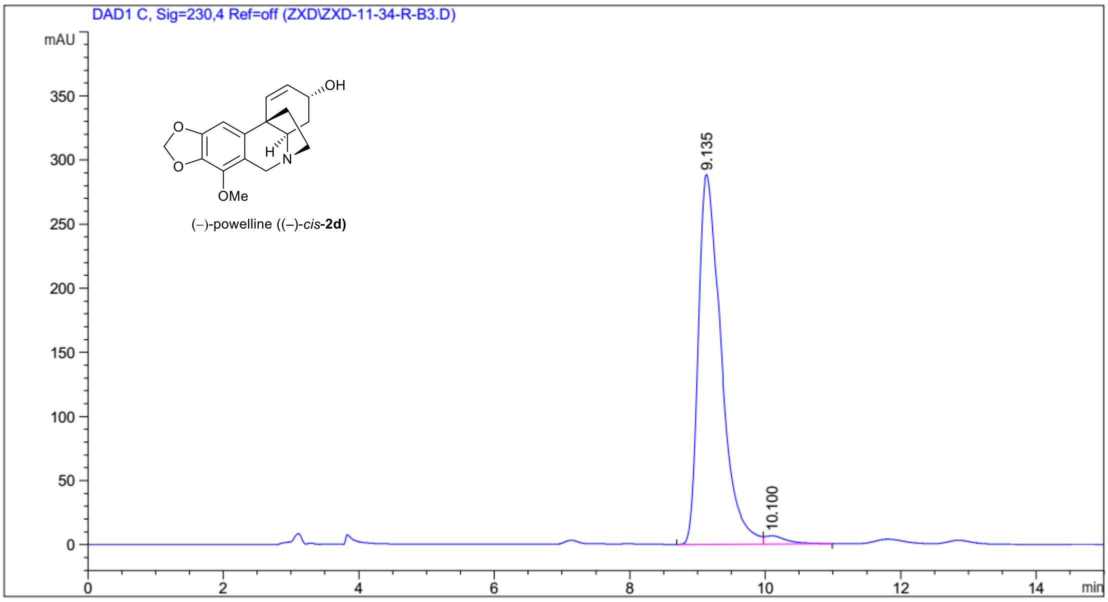

Peak #	RetTime [min]	Type	Width [min]	Area [mAU*s]	Height [mAU]	Area %
1	9.135	BV	0.3408	6742.57178	288.40390	97.9231
2	10.100	VB	0.3219	143.00761	6.32135	2.0769

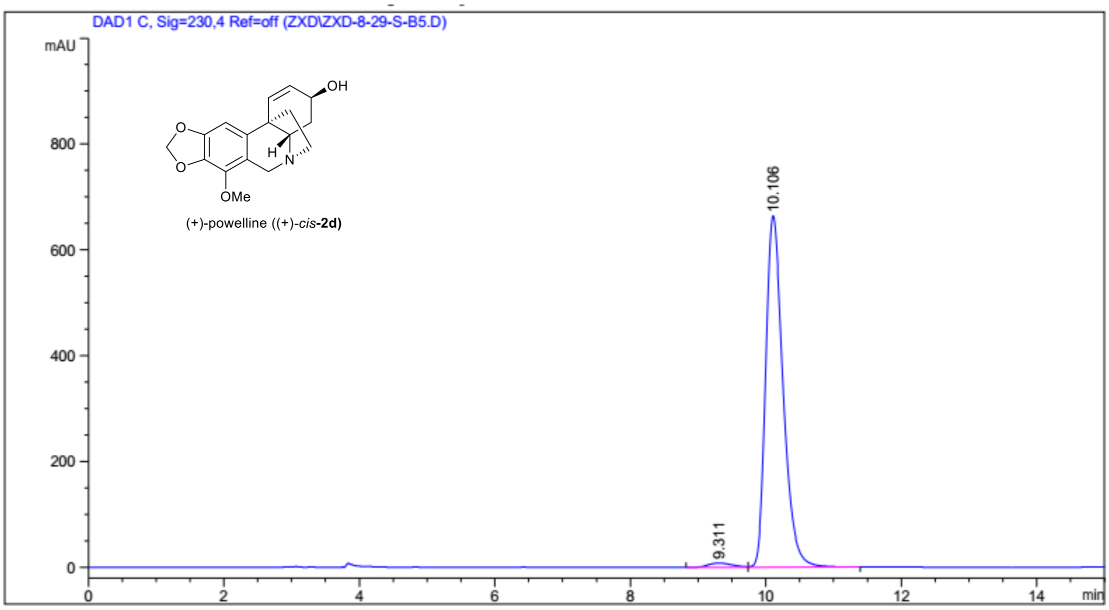

Peak #	RetTime [min]	Type	Width [min]	Area [mAU*s]	Height [mAU]	Area %
1	9.311	BV	0.4028	197.87384	7.83141	1.6447
2	10.106	VB	0.2739	1.18330e4	664.06653	98.3553

(+)-epipowelline ((+)-*trans*-2d) and (-)-epipowelline ((-)-*trans*-2d)

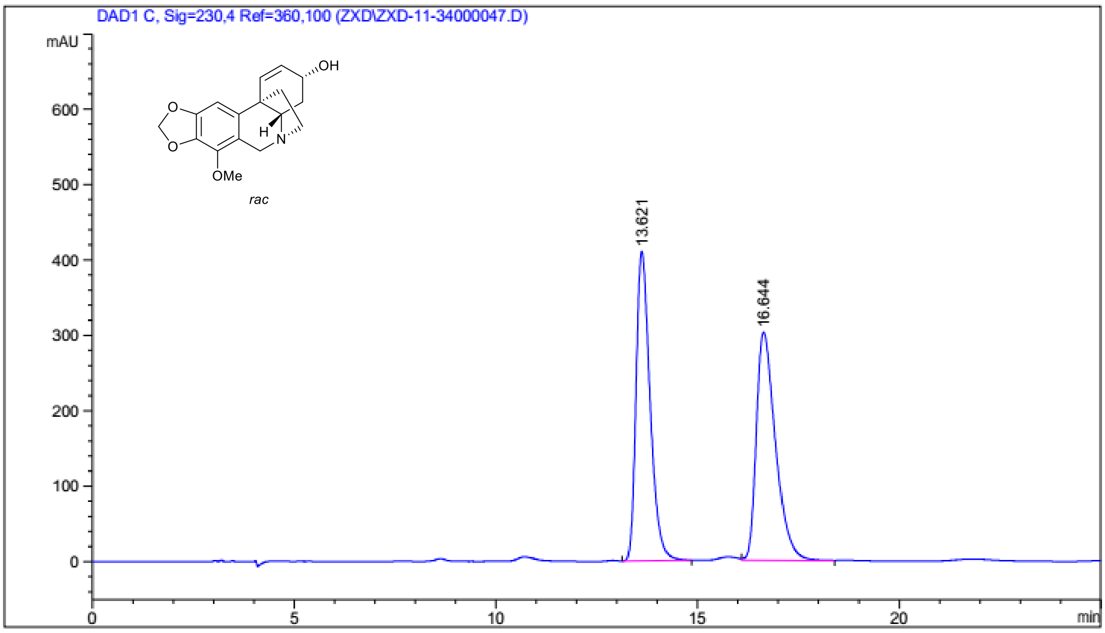

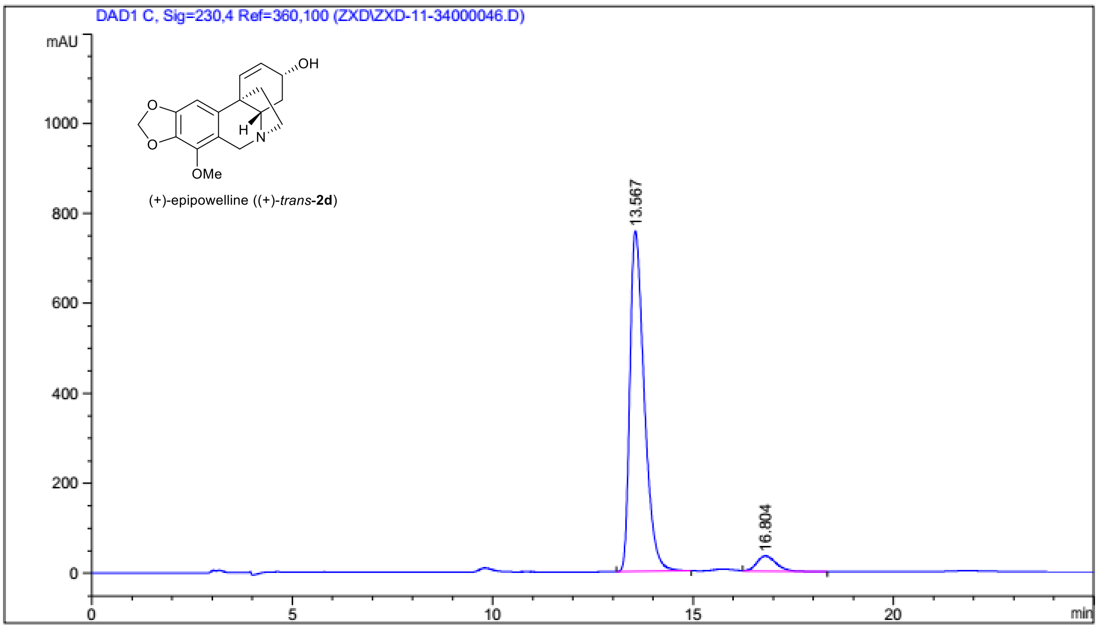

Peak #	RetTime [min]	Type	Width [min]	Area [mAU*s]	Height [mAU]	Area %
1	13.567	BB	0.3845	1.89903e4	757.48267	94.2000
2	16.804	VB	0.5138	1169.26147	34.46783	5.8000

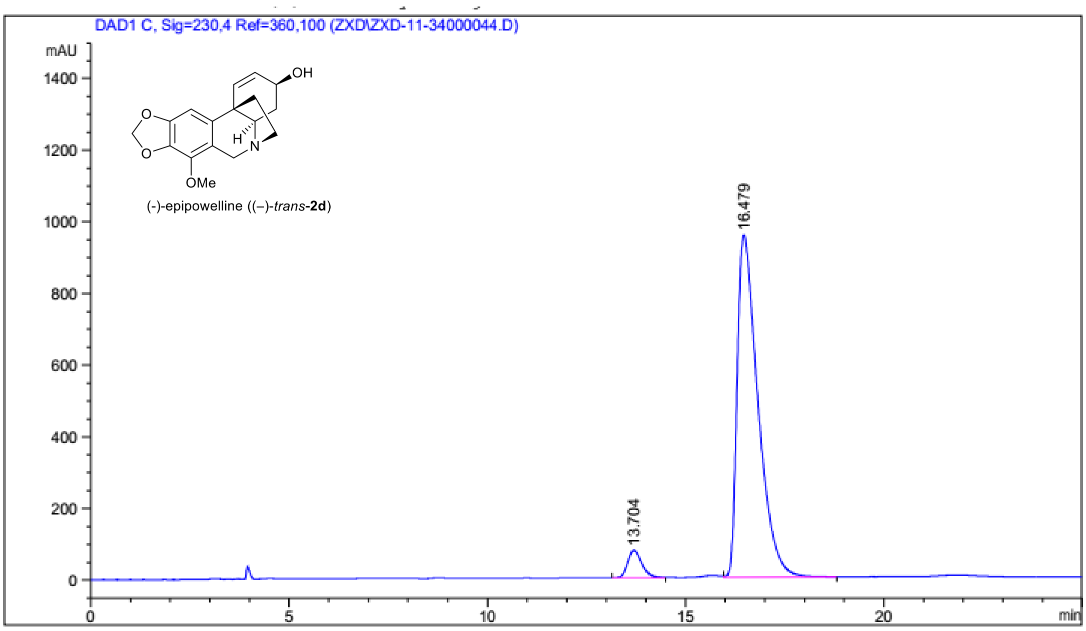

Peak #	RetTime [min]	Type	Width [min]	Area [mAU*s]	Height [mAU]	Area %
1	13.704	BV	0.3852	1913.77881	76.67959	5.2476
2	16.479	VB	0.5420	3.45561e4	955.34027	94.7524

(−)-*cis*-2e and (+)-*cis*-2e

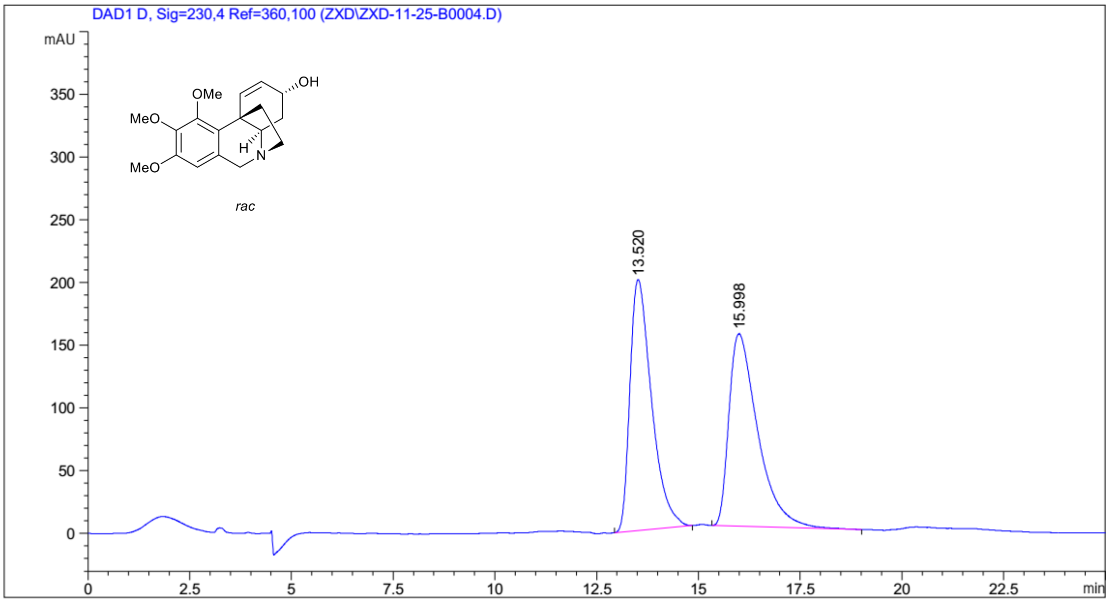

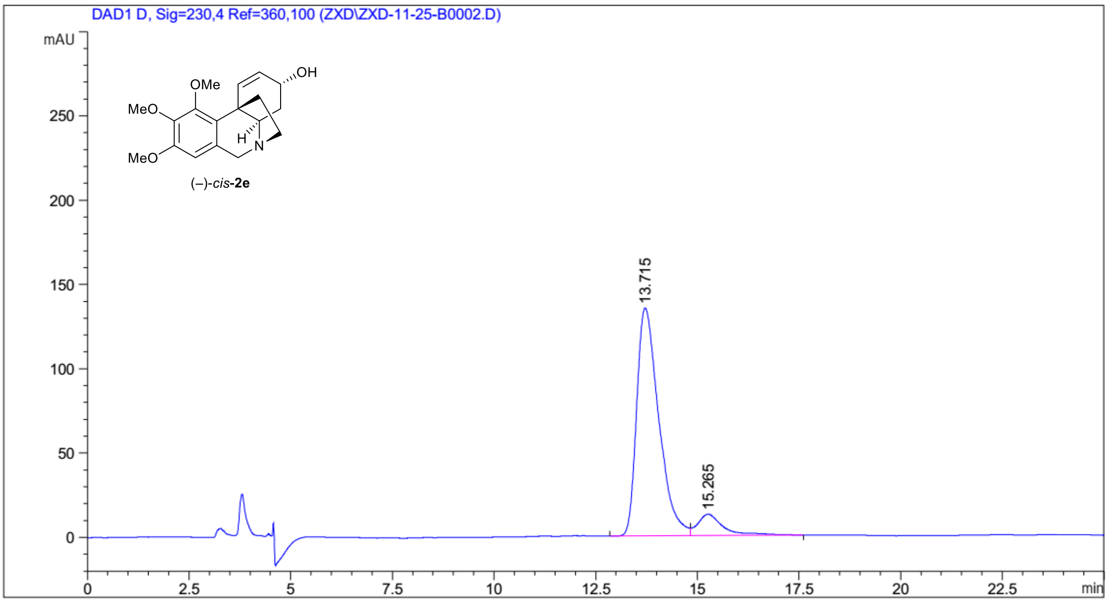

Peak #	RetTime [min]	Type	Width [min]	Area [mAU*s]	Height [mAU]	Area %
1	13.715	BV	0.5700	9876.88574	263.10541	92.8734
2	15.260	VB	0.5311	757.89844	20.80258	7.1266

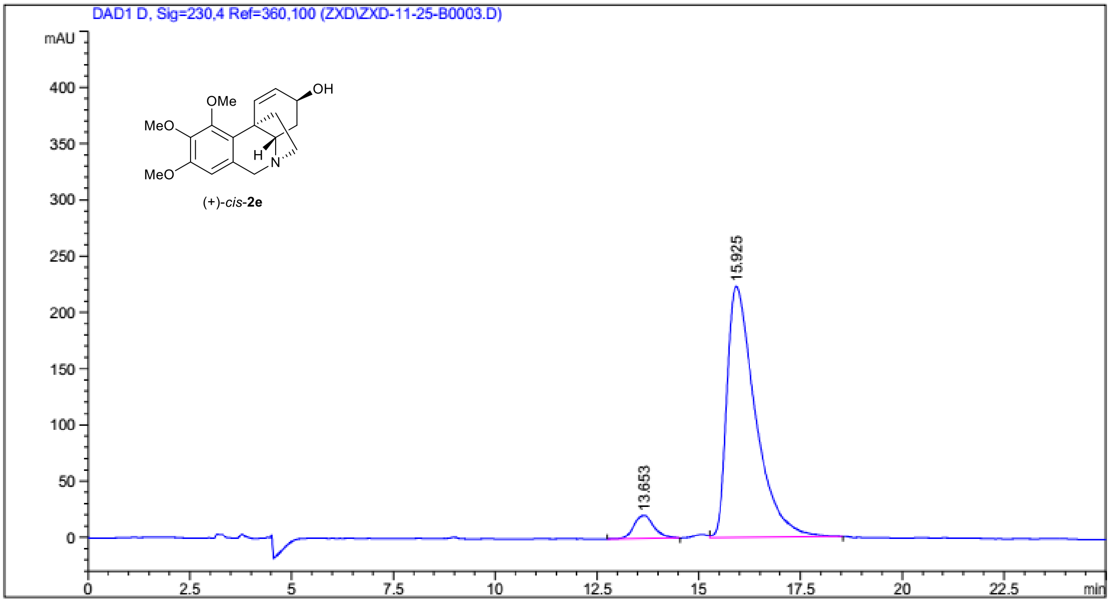

Peak #	RetTime [min]	Type	Width [min]	Area [mAU*s]	Height [mAU]	Area %
1	13.653	BB	0.5184	1399.78394	40.78712	6.2237
2	15.925	VB	0.7310	2.10915e4	434.00046	93.7763

(+)-*trans*-2e and (−)-*trans*-2e

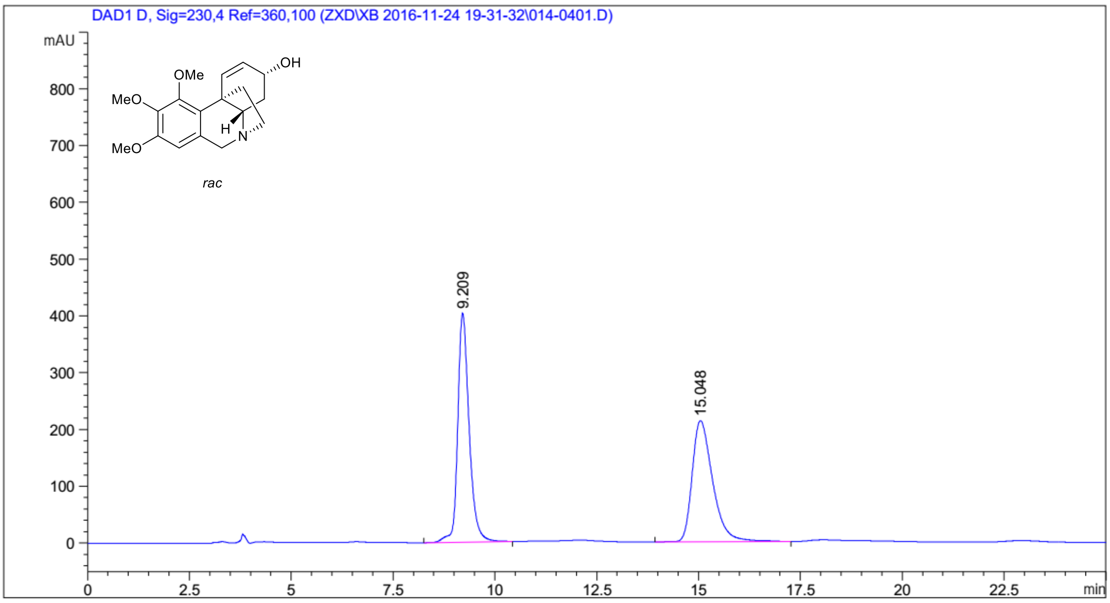

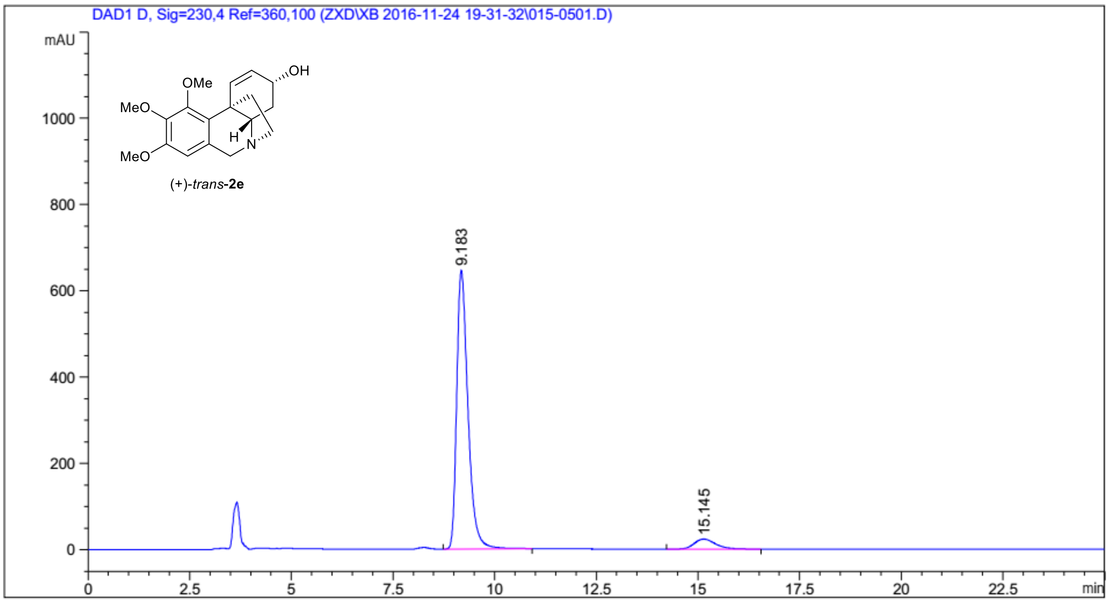

Peak #	RetTime [min]	Type	Width [min]	Area [mAU*s]	Height [mAU]	Area %
1	9.183	BB	0.2995	2.13985e4	1097.15417	93.3541
2	15.147	BB	0.5560	1523.36731	40.76593	6.6459

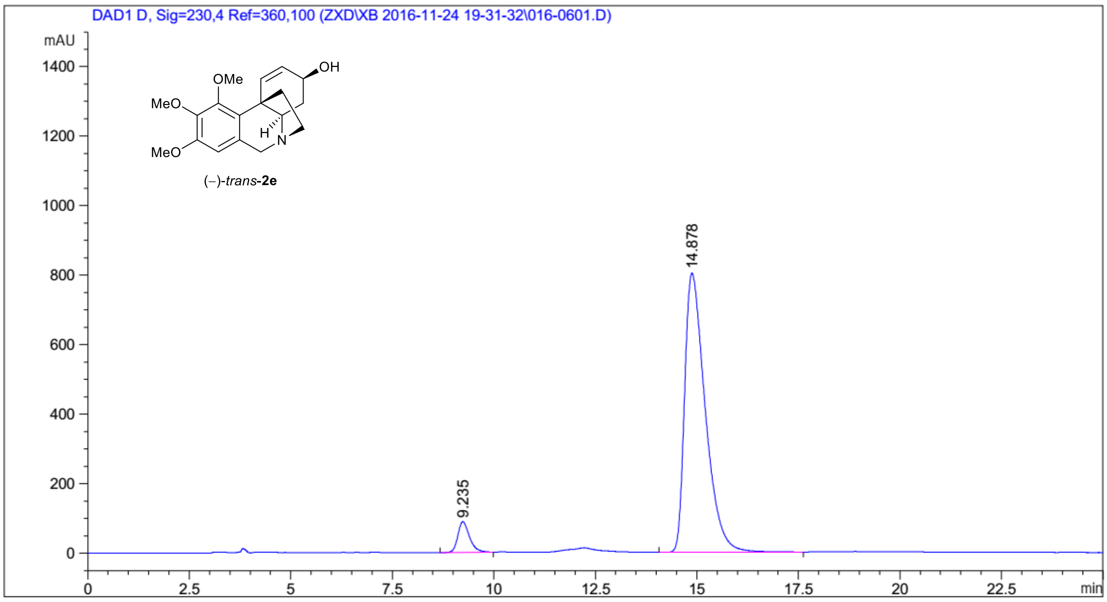

Peak #	RetTime [min]	Type	Width [min]	Area [mAU*s]	Height [mAU]	Area %
1	9.235	BB	0.3054	3162.37329	156.71118	6.1575
2	14.878	BB	0.5535	4.81957e4	1334.14160	93.8425
